# Discovery of Novel Isoxazole-Based Small-Molecule Toll-Like Receptor 8 Antagonists

**DOI:** 10.1021/acs.jmedchem.4c03148

**Published:** 2025-02-14

**Authors:** Troy Matziol, Valerij Talagayev, Tjaša Slokan, Nika Strašek Benedik, Janine Holze, Matej Sova, Gerhard Wolber, Günther Weindl

**Affiliations:** † Pharmaceutical Institute, Pharmacology and Toxicology Section, 200981 University of Bonn, Gerhard-Domagk-Street 3, 53121 Bonn, Germany; ‡ Institute of Pharmacy, Pharmaceutical and Medicinal ChemistryFreie, 9166 Universität Berlin, Königin-Luise-Street 2 + 4, 14195 Berlin, Germany; § Faculty of Pharmacy, the Department of Pharmaceutical Chemistry, 37663 University of Ljubljana, Aškerčeva 7, SI-1000 Ljubljana, Slovenia

## Abstract

Toll-like receptor 8 (TLR8) recognizes viral and bacterial RNA, initiating inflammatory responses that are crucial for innate immunity. Dysregulated TLR8 signaling contributes to autoimmune diseases, including systemic lupus erythematosus and rheumatoid arthritis, driving chronic inflammation and tissue damage. Therefore, targeting TLR8 has gained attention as a promising therapeutic strategy. We report a novel selective TLR8 antagonist scaffold identified through computational modeling and simulation. In silico-guided rational drug design and synthesis led to potent isoxazole-based compounds that were characterized by structure–activity relationships. The most active compounds inhibited TLR8-mediated signaling in cell lines and primary cells, reduced MyD88 recruitment, suppressed NF-κB- and IRF-dependent signaling, and decreased inflammatory responses. In silico and pharmacological analyses demonstrated competitive binding to the pocket of chemical ligands within the TLR8 dimerization interface. These highly selective and potent TLR8 antagonists possess favorable physicochemical properties, representing potential clinical candidates for TLR8-targeted therapy.

## Graphical abstract

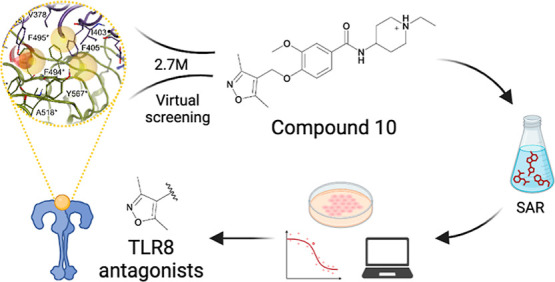

## Introduction

Toll-like receptors (TLRs) are germline-encoded pattern recognition receptors (PRRs) that initiate immune responses by recognizing pathogen-associated molecular patterns (PAMPs) and damage-associated molecular patterns (DAMPs) derived from pathogens or damaged cells. As critical components of the innate immune system, TLRs play a key role in immune defense.^1,2^ The human TLR family consists of TLR1-10, with TLR3/7/8/9 localized on endosomal membranes.^3^ Among the endosomal TLRs, TLR8 has emerged as a pivotal regulator in various immune-mediated disorders due to its distinct signaling pathways and biological functions.^4,5^ Primarily expressed in innate immune cells, such as monocytes, macrophages, and dendritic cells, TLR8 recognizes single-stranded RNA molecules from pathogens or damaged host cells.^6–8^ This recognition leads to the recruitment of adaptor protein MyD88 and the activation of downstream signaling pathways involving nuclear factor-κB (NF-κB) and interferon regulatory factors (IRFs). Dysregulated TLR8 signaling has been implicated in the pathogenesis of autoimmune diseases, including systemic lupus erythematosus (SLE) and rheumatoid arthritis (RA), where persistent TLR8 activation contributes to chronic inflammation and tissue damage.^9–11^ TLR8 has also been recognized to play a significant role in the regulation of viral infections.^12,13^

The therapeutic potential of targeting TLR8 has recently generated significant interest, particularly in the development of TLR8 antagonists.^6,14–17^ Selective TLR8 antagonists are designed to modulate aberrant TLR8 signaling and restore immune homeostasis in diseases characterized by TLR8 hyperactivity. For example, small-molecule TLR8 antagonists have demonstrated potential for modulating cytokine levels in autoimmune diseases such as SLE and RA.^14,18^ Several TLR7/8 ligands, including small molecules and antibodies, have been identified and are currently in preclinical and clinical trials.^19,20^ Enpatoran (M5049), a synthetic small-molecule TLR7/8 antagonist, is currently being investigated as a potential treatment for SLE.^21^ However, selective TLR8 antagonists have yet to be fully explored in clinical studies.

No naturally occurring antagonists of TLR8 have been identified to date. A recent preprint suggests that endogenous 2′-*O*-methyl guanosine RNA fragments might act as antagonists of TLR7/8.^22^ Unlike agonists, which benefit from a synergistic effect that enhances the affinity of uridine, a common RNA-building block found in animals and viruses, similar synergistic mechanisms for antagonist recognition have yet to be identified. Nonetheless, advances in understanding TLR8 pharmacology have significantly facilitated the development of TLR8 antagonists. Structural studies of the inactivated TLR8 dimer bound to antagonists have offered valuable insights into how TLR8 activity can be regulated. Despite this progress, research into the inhibitory mechanisms of TLR8 remain in its early stages.^14,18^ By targeting specific regions within the TLR8 receptor, novel antagonists can block ligand recognition and disrupt downstream signaling events.^23^ Previously, we have identified selective TLR8 antagonists through structure-based virtual screening approaches, which inhibited TLR8-mediated responses in the low μM range,^16,17^ and potent TLR8 antagonists have been described as tool compounds in the literature.^14,15^

In this study, we identified novel, highly potent small-molecule TLR8 antagonists through a structure–activity relationship (SAR)-based approach. Extensive biological characterization of the active virtual hits revealed compound **10** as a potential clinical candidate. Compound **10** demonstrated high selectivity, potency, and bioavailability as a competitive antagonist of TLR8 with no observed cytotoxic effects.

## Results

### Virtual Screening Identifies Novel Isoxazole Scaffold

To investigate the interactions between known TLR8 antagonists and the receptor, we analyzed all recently published crystal structures containing cocrystallized TLR8 (RCSB PDB: 6KYA, 6TY5, 6V9U, 6ZJZ, 7R52, 7R53, 7R54, 7RC9)^21,24–27^ (Figure S1). These structures consistently revealed that the antagonists bind at the uridine-binding site,^15,28,29^ located at the interface of the TLR8 homodimer (Figure 1A). Within this binding site, all cocrystallized antagonists establish a key hydrogen bond acceptor interaction with the backbone amide of G351, a central feature of their binding mode.^18,21,24–27^ Surrounding G351, residues such as F494, F495, Y348*, and V378* (where asterisks denote chain B residues) contribute additional hydrophobic contacts through their lipophilic side chains. Ionic interactions between E427 and the aliphatic amine groups of several antagonists (as observed in 6TY5, 6 V9U, 6ZJZ, 7R53, 7R54, and 7RC9)^21,24–27^ further stabilize binding (Figure 1B). Using LigandScout,^30^ we developed a 3D pharmacophore^31–33^ to represent the common binding features of these antagonists, using the coordinate frame of PDB entry 7RC9 ^30^ as a reference. The pharmacophore highlights essential interactions within the binding pocket including a hydrogen bond acceptor near G351, a positively ionizable feature adjacent to E427, and three hydrophobic features. These hydrophobic features align with key residues: the first near Y348, K350, and F494*; the second near V378 and F405; and the third close to I403, F494*, and A518* (Figure 1C,D). Together, these features encapsulate the shared interaction profile of TLR8 antagonists, providing a basis for a 3D pharmacophore virtual screening campaign.

**Figure 1:**
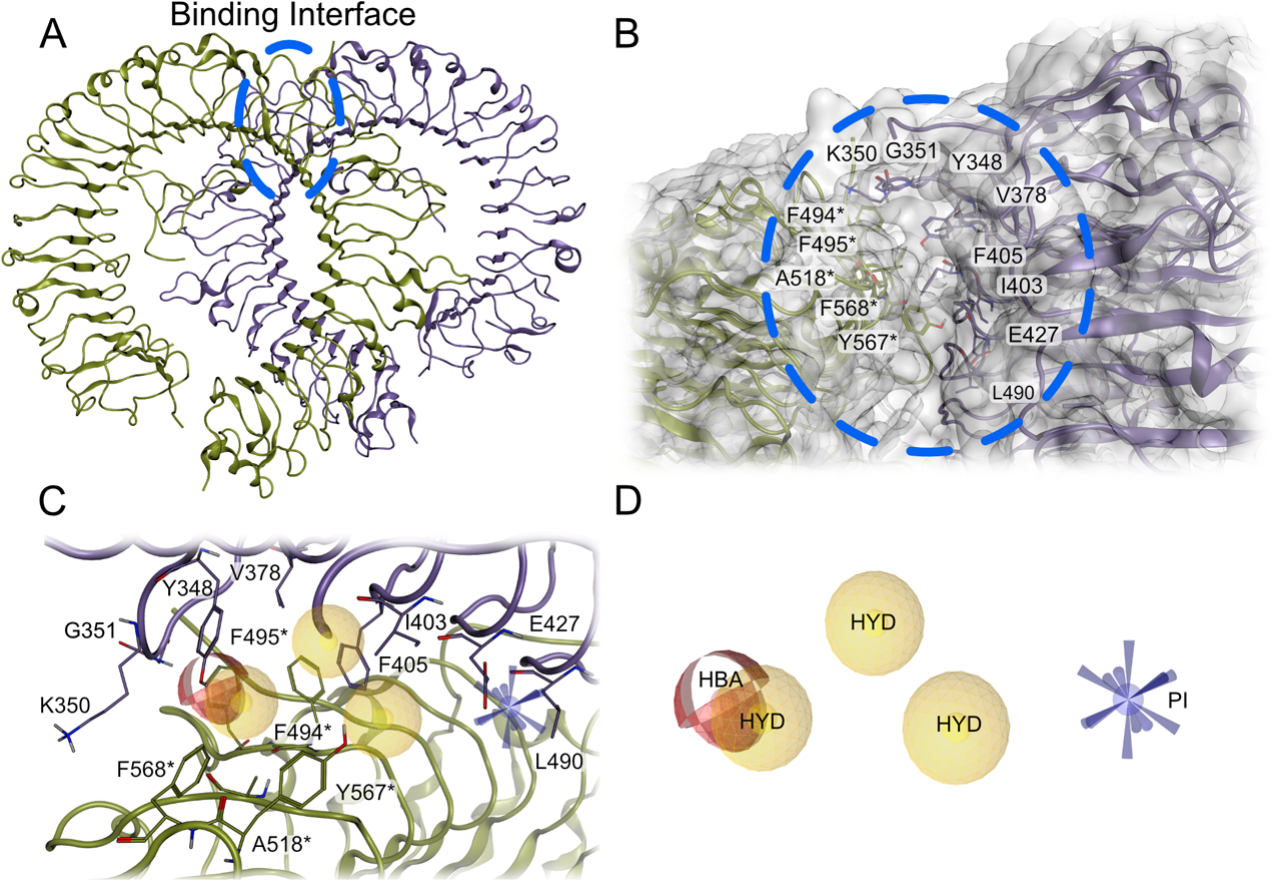
Structure of TLR8 binding interface with virtual screening 3D pharmacophore. (A) Structure of TLR8 with the binding interface of the homodimers. (B) Zoom view of the TLR8 antagonist binding site. (C) Binding site of TLR8 antagonists with the generated 3D pharmacophore. (D) 3D pharmacophore,^31–33^ which was used for virtual screening. The asterisk denotes the chain B. Color code: yellow spheres, hydrophobic contacts (HYD); red spheres, hydrogen bond acceptor (HBA); blue sphere with cones, positive ionizable (PI); purple and green ribbons and atoms, TLR8 homodimers; gray surface, TLR8 protein surface.

To understand how the 3D pharmacophore would perform in discriminating between active and inactive compounds, we statistically validated the 3D pharmacophore using two sets of compounds: the first, Set A, consisted of 158 known active molecules retrieved from ChEMBL^34–36^ (IC_50_ < 1000 nM), in addition to 231 known inactive compounds from ChEMBL (labeled as inactive or IC_50_ > 1000 nM), as well as 8406 decoys with physicochemical properties similar to the active molecules. The validation with both active and inactive compounds yielded only true positives, showing that all the active compounds were correctly recognized as active, while the validation of active molecules with diverse molecules with physicochemical properties similar to those of the active compounds yielded 58 false positives, showing that the pharmacophore recognizes diverse molecules with similar physicochemical properties as active molecules. To test the ability of the 3D pharmacophore to identify compounds that have low IC_50_ values, a second validation of Set B was performed using compounds with IC_50_ values below 50 nM. Thus, 47 compounds with IC_50_ values below 50 nM were labeled as actives and were validated with the previously mentioned 231 inactive compounds and 2538 molecules with physicochemical properties similar to the active compounds from Set B, generated from the active compounds of set B. This validation showed that against the inactive compounds, 19 out of 47 true positive compounds of the set B were retrieved with no false positive virtual screening hits, while the validation with the molecules with physicochemical properties similar to the active compounds from set B resulted in 29 false positive hits (Figure S2A–D), showing overall good performance in retrospective model validation.

Following the retrospective validation of the 3D pharmacophore model, we performed a prospective virtual screening using a commercial library of 2.7 million compounds to obtain potentially potent compounds, yielding 18310 virtual hits. These were then subjected to a two-step consensus molecular docking protocol using the crystal structure with the highest resolution (PDB entry 7RC9)^30^ (see Experimental Section: Molecular Docking). This molecular docking protocol resulted in 42 compounds with 5 compounds containing an already known dimethoxyphenyl scaffold,^37^ 22 compounds containing an already known pyridine scaffold,^37^ and 12 compounds containing a novel isoxazole scaffold. We selected the isoxazole scaffold compounds for further experimental testing due to their novelty (Table S1).

### Biological Characterization of Hit Compounds

To experimentally confirm our in silico predictions, the 12 hit compounds were tested for their activity and cell viability in hTLR8-HEK293 reporter cells. None of the compounds showed agonistic effects (Figure S3A). Four of the 12 compounds, representing a hit rate of 33%, showed no cytotoxic effects (Figure S3B) and over 75% inhibition of NF-κB at 10 μM (Figure S3C and Table S2) when stimulated with the TLR8 agonist TL8-506, and therefore were selected for IC_50_ testing (Figure S4). Compounds **10** and **12** demonstrated IC_50_ values below 0.5 μM (Table 1) and were comparable to Enpatoran, a TLR7/8 dual antagonist currently under clinical investigation for SLE and cutaneous lupus erythematosus (CLE).^38^

**Table 1:**
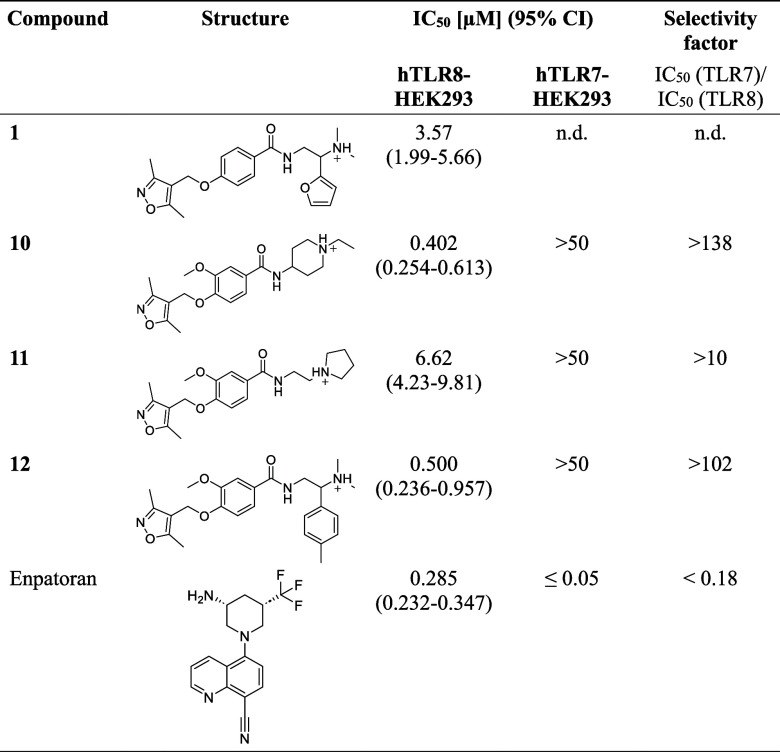
Potencies for Inhibition of NF-κB Activity in hTLR8- and hTLR7-HEK293 Reporter Cells^a^

a IC_50_ values were calculated from concentration–response curves (Figures S4 and S5). n.d. = not determined.

Given the strong homology between TLR7 and TLR8 and their many common ligands,^28^ achieving high selectivity for TLR8 is challenging. To verify the selectivity, we tested compounds **10**, **11**, and **12** for IC_50_ in hTLR7-HEK293 reporter cells. Compounds **10** and **12** demonstrated >100-fold selectivity, indicating a strong preference for TLR8 over TLR7 (Table 1 and Figure S5). TLR8 preference was confirmed for compound **11**, a close analogue of compound **10** but with lower potency at TLR8. In contrast, Enpatoran preferentially inhibited TLR7-mediated responses with a TLR7/TLR8 selectivity factor below 0.05.

### Structure–Activity Relationship of Isoxazole-Based TLR8 Antagonists

The promising inhibitory effects of compounds with an isoxazole scaffold prompted us to explore the chemical space by in silico-guided chemical synthesis to decipher the binding mode and structural basis of TLR8 inhibition. An in silico SAR evaluation was performed to analyze the binding modes of the derivatives and identify the differences in the interactions of those with the protein. For this, we performed molecular docking studies. The main differences in the derivatives consisted of the presence or absence of a methoxy group on the phenyl ring in addition to the difference of the substituent in the para position of the phenyl ring. The length of the substituent played an important role in the SAR as shown by compounds **34** and **35**. These compounds mainly differ in the substituent on the piperidine ring, with compound **34** having a methyl group, while **35** contains an isopropyl moiety (Figure 2A). The length of the isopropyl moiety allows **35** to form an additional hydrophobic interaction with Leu490, facilitated by the closer proximity of the isopropyl group compared to the shorter methyl group of **38**. Thus, the main difference in these compounds is the hydrophobic interaction with Leu490, which affects the activity of the compound. Another important aspect of the SAR analysis was the presence or absence of the methoxy group. Examples of this analysis are compounds **31** and **38**. Compound **31**, which contains the methoxy group, loses the hydrophobic contact with Ala518* that is present in compound **38**; thus, the absence of the methoxy group increases the potency of **31** (Figure 2B). These results highlight the importance of the substituent length and the methoxy group on the potency of the compounds.

**Figure 2:**
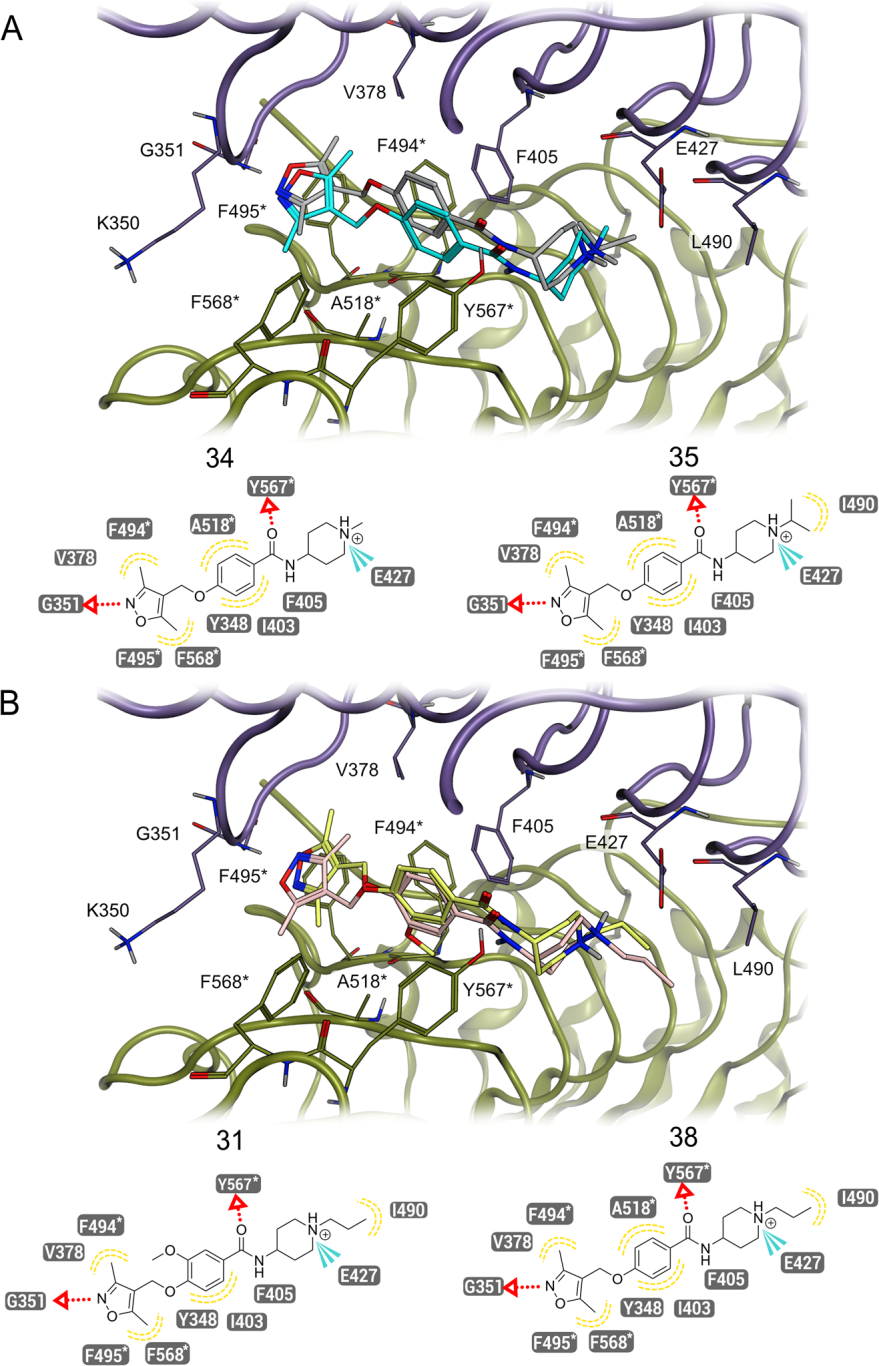
In silico SAR study of compounds **34**, **35**, **31**, and **38**. (A) Compound **34** has a methyl group at the piperidine ring and thus is not able to perform a hydrophobic interaction with Leu40, while compound **35** has an isopropyl group at the piperidine, which leads to an additional hydrophobic interaction with L490. (B) Compound **31** has a methoxy group at the phenyl ring, which leads to the loss of the hydrophobic interaction with Ala518*. Compound **38**, which lacks the methoxy group at the phenyl ring, shows a hydrophobic interaction with Ala518*. The asterisk denotes the chain B. Color code: purple and green ribbons atoms, TLR8 homodimers; turquoise atoms, compound **34**; gray atoms, compound **35**; yellow atoms, compound **31**; pink atoms, compound **38**.

The synthesis began with methyl 4-hydroxybenzoate or methyl 4-hydroxy-3-methoxybenzoate, which was alkylated with 4-(chloromethyl)-3,5-dimethylisoxazole (Scheme 1). In the second step, the esters **13** and **14** were hydrolyzed with 1 M NaOH and the obtained carboxylic acids **15** and **16** were coupled with the appropriate amine using HATU as a coupling reagent to give amides **17**-**39**. 4-((3,5-Dimethylisoxazol-4-yl)­methoxy)­benzoic acid was also coupled with *tert*-butyl 4-aminopiperidine-1-carboxylate to give the amide **42**, and in the next step, the Boc protecting group was removed with trifluoroacetic acid. The obtained 4-((3,5-dimethylisoxazol-4-yl)­methoxy)-*N*-(piperidin-4-yl)­benzamide (**43**) was then alkylated with butyl or allyl bromide to give amides **40** and **41**, respectively.

**Scheme 1:**
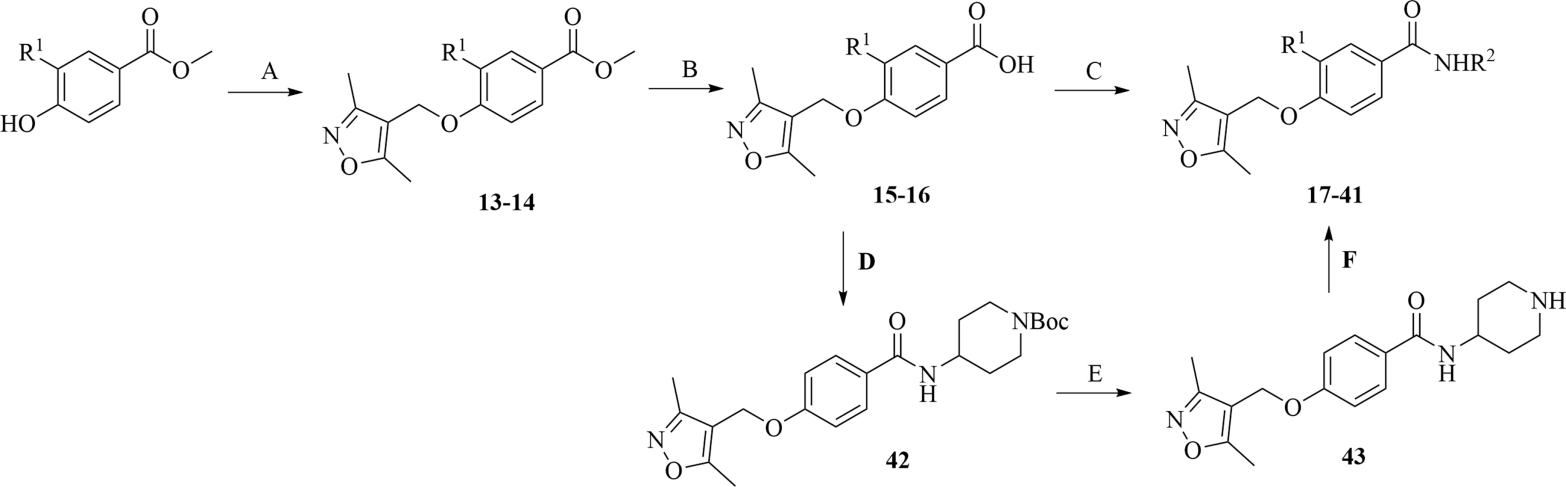
Synthesis of Isoxazole-Based Compounds.^a^

a Reagents and conditions:(A) K_2_CO_3_, 4-(chloromethyl)-3,5-dimethylisoxazole, KI, acetone, 50 °C, 24 h; (B) 1 M NaOH, 1,4-dioxane, rt, 24h.(C) HATU, amine (R^2^NH_2_), DIPEA, THF, rt, 24h;(D) HATU, tert-butyl 4-aminopiperidine-1-carboxylate, DIPEA, THF, rt, 24 h; (E) TFA, DCM, rt, 24 h; (F) alkyl bromide, K_2_CO_3_, KI, acetonitrile, reflux, 2 h

To optimize and evaluate the scaffold and in silico predictions, these synthesized compounds were screened as described above (Figure S6). Thirteen of 26 compounds showed over 80% inhibition at 10 μM (Table S3), and 12 were selected for IC_50_ testing (Figure S7). However, none of the compounds demonstrated IC_50_ values lower than those of compounds **10** and **12** from the initial screening (Table 2). Therefore, we proceeded with further biological characterization of these compounds. Nevertheless, we have obtained relevant information about the SAR. Among the synthesized compounds, the tertiary amines with piperidine ring (e.g., **26**, **31**, **34**, **35**, **38–41**; Tables S3 and S4) are the most potent TLR8 antagonists with IC_50_ values around 1 μM. As already mentioned above, the nature of the substituent on the piperidine ring is important since the compounds with a longer (e.g., **31**, **38**–**41**) or branched (e.g., **28** and **35**) substituent on the piperidine ring exhibited higher potency compared to the methyl-substituted or unsubstituted piperidine derivatives (e.g., **33**, **34**, **43**). The piperidine ring has to be alkylated since compound **43** with unsubstituted piperidine showed only 32% inhibition of NF-κB at 10 μM. The most optimal substituent on the piperidine ring is ethyl, which is present in compound **10** or allyl in the case of compounds without a methoxy group. In compounds **24** and **26**, the piperidine ring is inverted and bonded directly to the carbonyl group, resulting in the distance that is one atom shorter. The bound pyrrolidine and piperidine in the para position of the piperidine enable the activity of these compounds. In this case, the methoxy group on the phenyl ring is beneficial since derivative without the methoxy group (e.g., **21**) is less potent. Likewise, the methoxy derivative **10** is more potent than compound **39**, which does not possess a methoxy group. Furthermore, replacing the piperidine with pyrrolidine results in the less potent isoxazole derivatives **19**, **27**, and **29**. Piperazine derivatives showed only moderate (compounds **17**, **22**, and **23**) or low inhibition (compounds **30** and **36**) of NF-κB at 10 μM.

**Table 2:**
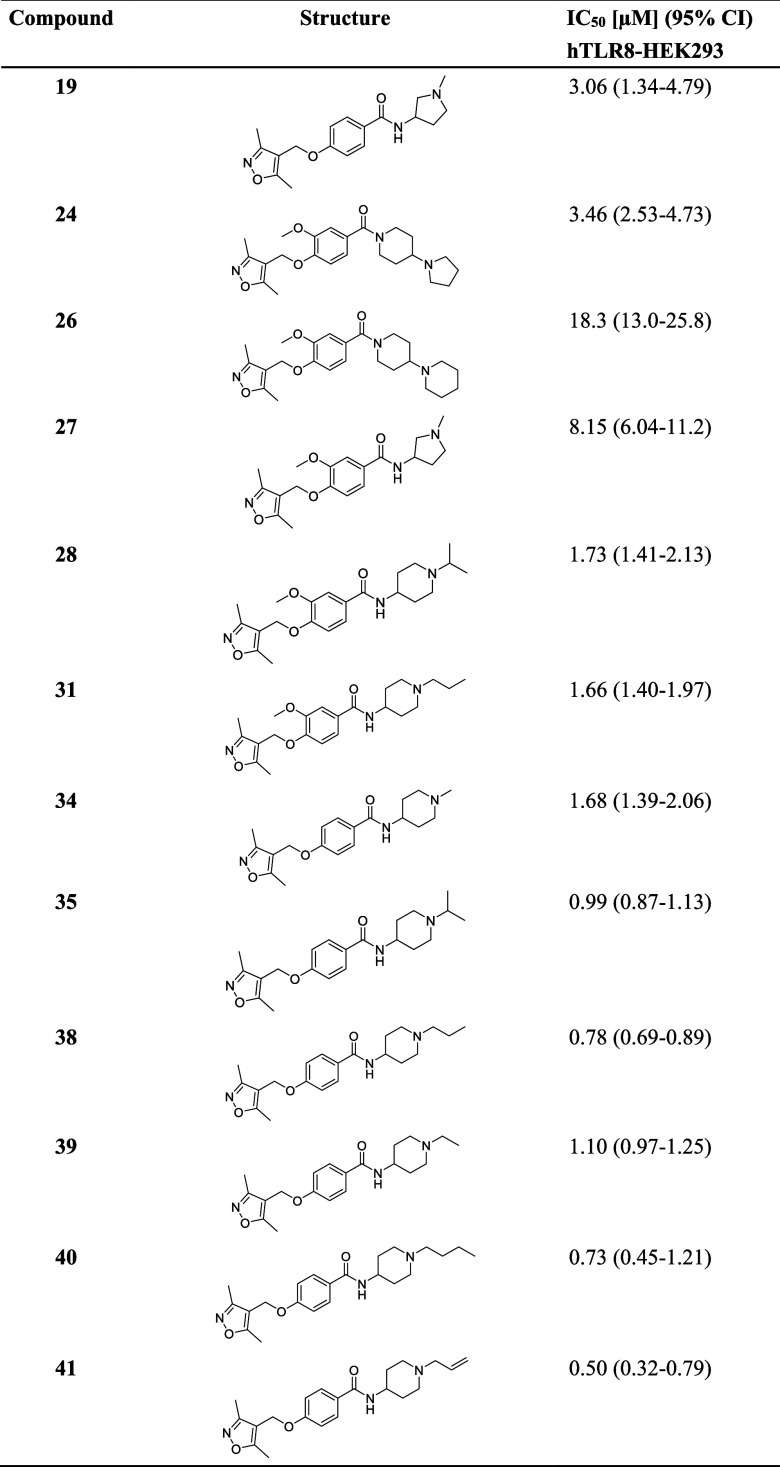
Potencies for Inhibition of NF-κB Activity in hTLR8-HEK293 Reporter Cells^a^

a IC_50_ values were calculated from concentration–response curves (Figure S7).

### Compounds **10** and **12** Selectively Inhibit TLR8-Mediated Inflammation and Signaling

Next, we examined the inhibitory effects of the two lead compounds across various assays, cell lines, and primary cells. Activation or inhibition of TLR8 modulates downstream signaling pathways, significantly influencing cytokine production.^39^ In THP-1 macrophages, compounds **10** and **12** demonstrated potent TLR8-mediated TNF inhibition with IC_50_ values of 0.037 and 0.12 μM, respectively (Figure 3A and Table 3). Cell viability remained unaffected up to 100 μM (Figure S8A). TNF production was selectively inhibited at 10 μM in response to the TLR8 agonists TL8-506 or CL075, without influencing responses to TLR2 or TLR4 agonists (Figure 3B). To reflect more physiologically relevant conditions, we tested peripheral mononuclear blood cells (PBMCs), which express various TLRs. Both compounds **10** (IC_50_ = 1.02 μM) and **12** (IC_50_ = 1.15 μM) showed lower potency than Enpatoran (IC_50_ = 0.074 μM) (Figure 3C and Table 3) without affecting cell viability up to 100 μM (Figure S8B).

**Figure 3:**
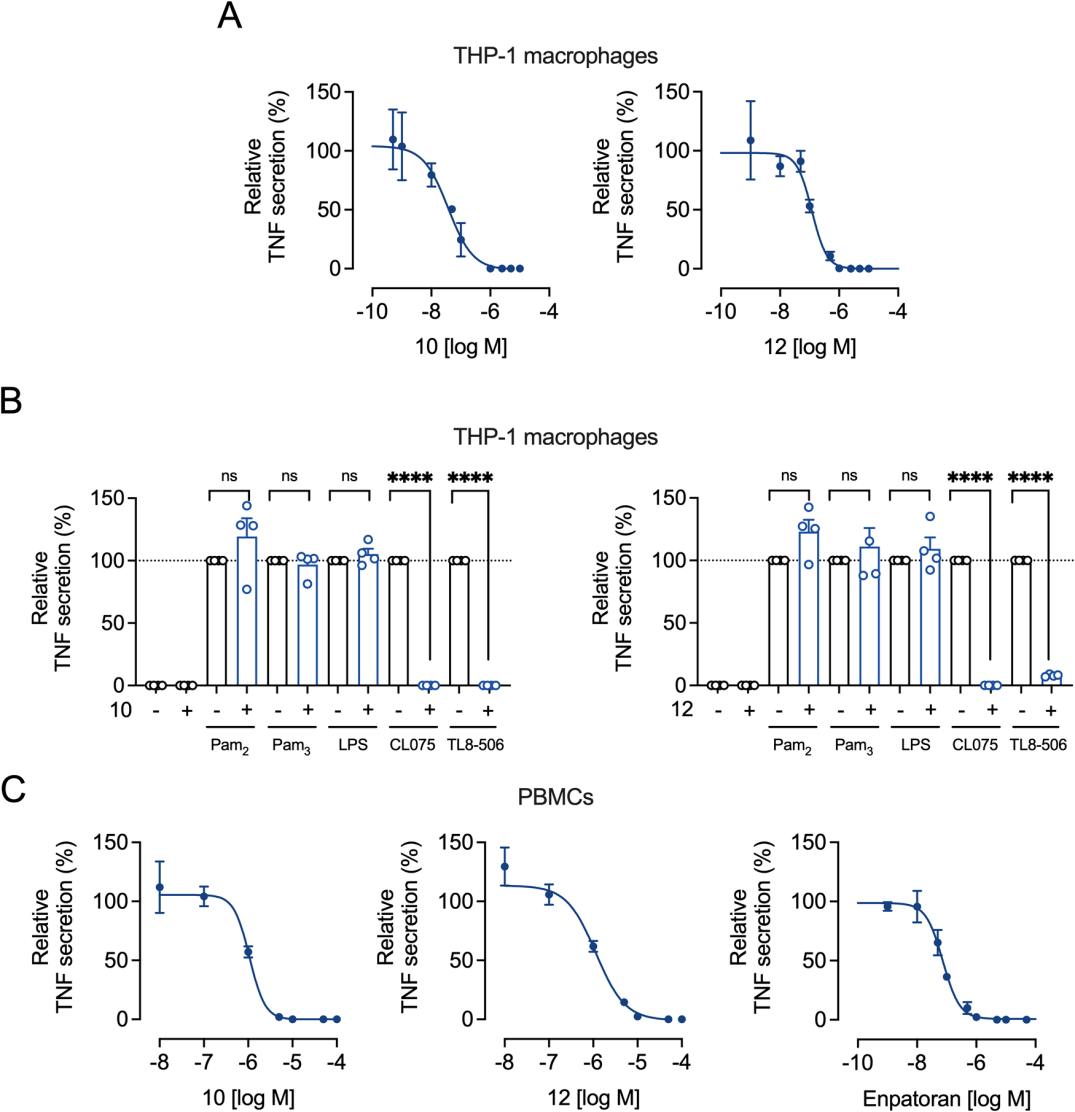
Potency and selectivity of compounds **10** and **12** in THP-1 macrophages and PBMCs. (A) THP-1 macrophages or (C) PBMCs were preincubated with increasing concentrations of the test compounds for 1 h and then stimulated with TL8-506 (0.6 μM) for 4 h. TNF release in the cell culture supernatants was determined by ELISA. For the calculation of concentration–response curves, nonlinear regression with variable slope (four parameters) was used. IC_50_ values are shown in Table 3. Mean ± SEM (*n* = 3). (B) THP-1 macrophages were preincubated with 10 μM of compound **10** or **12** for 1 h and then stimulated with TLR ligands Pam_3_CSK_4_ (100 ng/mL), Pam_2_CSK_4_ (10 ng/mL), LPS (10 ng/mL), CL075 (4 μM), and TL8-506 (3 μM) for 24 h. TNF release in the cell culture supernatants was determined by ELISA. Mean + SEM (*n* = 4). One-sample *t*-test, ns ≥0.05, *P ≤ 0.05, **P ≤ 0.01 ***P ≤ 0.001, ****P ≤ 0.0001.

**Table 3:** Potencies for Inhibition of TL8-506-Stimulated TNF Release in THP-1 Macrophages and PBMCs^a^

compound	IC_50_ [μM] (95% CI)	
	THP-1 macrophages	PBMCs
**10**	0.037 (0.020–0.066)	1.07 (0.76–1.53)
**12**	0.120 (0.090–0.172)	1.15 (0.71–1.76)
Enpatoran	n.d.	0.074 (0.058–0.096)

a IC_50_ values were calculated from concentration–response curves (Figure 3A, C). n.d. = not determined.

Since compound **10** showed improved potency over compound **12** in all pharmacological parameters, we decided to further characterize this compound. To evaluate TLR specificity, compound **10** was tested against ligands for TLR2/1, TLR2/6, TLR3, TLR4, TLR5, TLR7, TLR8, and TLR9 using HEK293 reporter cells. The results demonstrated selective inhibition of TLR8, with no significant activity against other TLRs, confirming its high selectivity (Figure 4A). To confirm cytokine inhibition at the gene level, we analyzed mRNA expression levels of *TNF* and *IL1B* in TL8-506-stimulated THP-1 macrophages, which were significantly inhibited in the presence of compound **10** (Figure 4B). In general, IL-1β is released after priming and activation of macrophages.^40^ TLR8 priming and subsequent NLRP3 activation by nigericin or ATP triggered IL-1β release, which was blocked by compound **10** (Figure 4C). Cell death was induced by the inflammasome activators in both primed and unprimed cells to the same extent and not reduced by the TLR8 antagonist (Figure 4D). Further investigation of upstream proteins involved in the NF-κB pathway demonstrated that compound **10** reduced p-NF-κB p65 and increased IκBα levels following TLR8 activation with TL8-506 (Figure 4E). When stimulated, respectively, with TLR8 and TLR4 agonists in THP1-Dual TLR4/MD-2/CD14 cells (hereafter referred to as THP-1 Dual cells), compound **10** showed selective and potent inhibition of TLR8-but not TLR4-mediated activation of the NF-κB and interferon regulatory factor (IRF) pathways (Figure 4F). Given that NF-κB and IRF activation in TLR8 occurs through the MyD88 pathway, we sought to determine whether compound **10** reduces the level of recruitment of MyD88 to the TLR8 receptor. While it is increasingly clear that TLR-induced myddosomes are long-lived and highly dynamic,^41^ coimmunoprecipitation analysis in THP-1 macrophages demonstrated reduced MyD88 recruitment to TLR8 in the presence of compound **10** (Figure 4G). To complement the traditional single-end point assays, we used optical biosensor technology to measure whole TLR-mediated cell responses in a label-free environment.^42^ In THP-1 Dual cells, no DMR signal was detected in the presence of compound **10** alone (Figure S9A), whereas TL8-506 and LPS induced a robust DMR signal during the recording period (Figure S9B,C). DMR signals were fully inhibited by high concentrations of compound **10** in TL8-506-stimulated cells (Figure S9B), while no inhibition was observed in the presence of LPS (Figure S9C). The concentration–effect curve for DMR signals at 250 min (Figure 4H) showed an IC_50_ of 0.855 μM (95% CI: 0.459–1.59 μM) for compound **10** in TLR8-activated cells.

**Figure 4:**
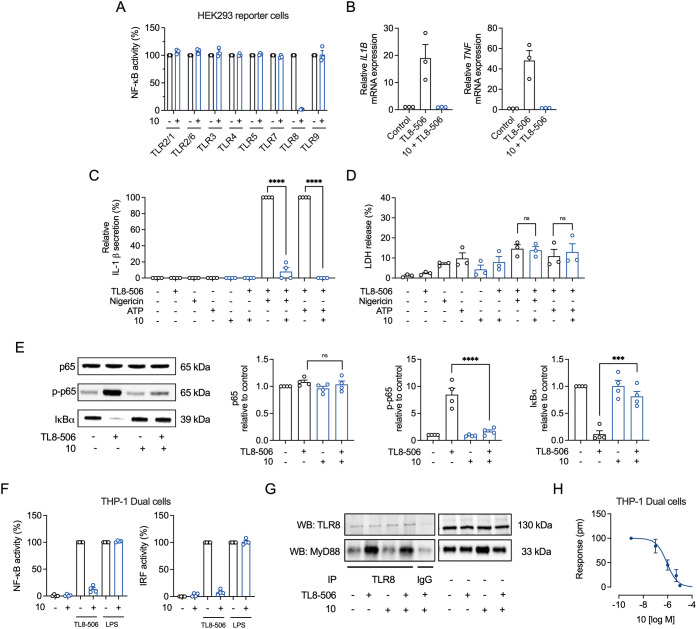
Compound **10** inhibits TLR8-mediated inflammation and signaling. (A) hTLR-HEK293 reporter cells were preincubated with or without compound **10** (10 μM) for 1 h and then stimulated with ligands for TLR2/1 (Pam_3_CSK_4_, 10 ng/mL), TLR2/6 (Pam_2_CSK_4_, 1 ng/mL), TLR3 (poly­(I:C) HMW, 10 μg/mL), TLR4 (LPS *E. coli*, 10 ng/mL), TLR5 (flagellin *B. subtilits*, 100 ng/mL), TLR7 (CL307, 0.8 μM), TLR8 (TL8-506, 1.5 μM), or TLR9 (ODN2006, 5 μM). Supernatants were analyzed for TLR-mediated NF-κB activation by the SEAP reporter assay using QuantiBlue (OD_620_) and normalized to the respective TLR agonist alone. Mean + SEM (*n* = 3). (B) THP-1 macrophages were preincubated with 10 μM of compound **10** for 1 h and then stimulated with TL8-506 (6 μM) for 1 h. *IL1B* and *TNF* gene expression was normalized to the housekeeping gene *GAPDH*, and values were compared to control (designated with a value of 1). Bar graphs show mean + SEM (*n* = 3). (C,D) THP-1 macrophages were primed with TL8-506 (0.6 μM) for 3 h and then stimulated for 3 h with ATP (5 mM). Compound **10** (10 μM) was added after priming for 1 h before stimulation. (C) IL-1β release in the cell culture supernatants was determined by ELISA. (D) LDH release was determined in cell culture supernatants. Results are shown as percentage of the maximum LDH release. Mean + SEM (*n* = 4). (E) THP-1 macrophages were incubated with compound **10** (10 μM) for 1 h and stimulated with TL8-506 (0.6 μM) for 15 min. Whole-cell lysates were used for WB. Bar graphs were obtained by densitometric analysis of Western blot data. Uncropped Western blots are shown in Figure S10A. Mean + SEM (*n* = 4). One-way ANOVA followed by Tukey’s post-test, ns ≥0.05, *P ≤ 0.05, **P ≤ 0.01 ***P ≤ 0.001, ****P ≤ 0.0001. (F) THP1-Dual TLR4/MD-2/CD14 cells (THP-1 Dual cells) were preincubated with 10 μM of compound **10** for 1 h and then stimulated with TL8-506 (6 μM) or LPS from *E. coli* (10 ng/mL) for 24 h. Supernatants were analyzed for NF-κB activation by the SEAP reporter assay using QuantiBlue (OD_620_) or for IRF activation by lucia luciferase using QuantiLuc. (G) THP-1 macrophages were incubated with compound **10** (10 μM) for 1 h, followed by TL8-506 (0.6 μM) stimulation for 30 min. Co-IP was performed using anti-hTLR8 antibodies and is depicted as Western blots (WB) for hTLR8 and MyD88. Uncropped Western blots are shown in Figure S10B. Mean + SEM (*n* = 4). (H) Inhibitory concentration–response curves resulting from DMR traces. THP1-Dual TLR4/MD-2/CD14 cells (THP-1 Dual cells) were preincubated with increasing concentration of compound **10** and afterward stimulated with 6 μM TL8-506. The concentration–response curve was derived from DMR signals recorded at 250 min shown in Figure S9B. Mean ± SEM (*n* = 3).

### Binding Mode Characterization of Compound **10**

We performed in silico analysis of the binding mode of compound **10** to gain insight into the interactions between **10** and TLR8. We found that compound **10** shows a hydrogen bond interaction of the isoxazole with the backbone amide of G351 and a charge interaction of the protonated amine with the side chain of E427. The methyl groups of **10** form hydrophobic contacts with the side chains of Y348, K350, V378, F494*, F495*, and F568*. The phenyl ring forms hydrophobic contacts with the side chains of I403, F405, A518*, and Y567*, while the ethyl moiety establishes a hydrophobic contact with L490 (Figure 5A).

**Figure 5:**
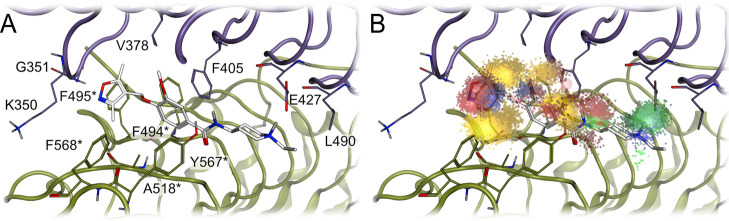
Toll-like receptor 8 binding interface with proposed binding mode of compound **10**. (A) TLR8 binding site interface with the hypothetical binding pose of **10**. (B) TLR8 binding site interface with the surmised binding pose of **10** and the Dynophore clouds representing the interactions between **10** and the TLR8 homodimer. The asterisk denotes the chain B. Color code: yellow clouds, hydrophobic contact; red clouds, hydrogen bond acceptor; green clouds, hydrogen bond donor; dark blue clouds, aromatic interaction; light blue cloud, ionic interaction; white atoms, compound **10**; purple and dark green ribbons and atoms, TLR8 homodimers.

To further characterize these interactions, we performed molecular dynamics (MD) simulations and analyzed the frequency of the interactions using our recently developed method Dynophores.^43–47^ This analysis method allowed us to evaluate the stability of the interactions throughout the simulation. The Dynophore analysis revealed that the isoxazole nitrogen maintains a hydrogen bond with the backbone amide of G351 for 80% of the simulation time. The methyl groups of the isoxazole show hydrophobic contacts with residues F261, Y348, V378, I403, F494*, F495*, A518*, and V520* during 100% of the simulation. The isoxazole ring shows π-interactions with Y348 and F495* during 44% of the simulation time. The phenyl ring shows hydrophobic contacts throughout the entire simulation duration with residues F261, I403, F405, F494*, A518*, and Y567*, while the charge interaction between the amine of **10** and E427 is formed during 92% of the simulation length (Figure 5B and Table S5).

To further evaluate the MD simulation results and to explore the binding cavity of TLR8, we conducted mutation studies. The starting point was an in silico inspection of the binding site of the cocrystallized ligand of PDB ID: 5WYZ.^48^ This structure was selected because of the size of the ligand and its suitability in the binding site. Additionally, it was a crystal structure that was not used in generating the 3D pharmacophore for the virtual screening campaign. We analyzed the binding site to identify potential residues that could be modified to affect the activity of compound **10**. The residues G351, V378, F495, and A518 were selected for in silico mutation studies based on their interactions with **10**. Among those residues, G351 is notable for forming a hydrogen bond acceptor interaction with compound **10**. Introducing a G351P mutation would disrupt the hydrogen bond interaction with **10** and allow us to evaluate the importance of this hydrogen bond interaction for TLR8 inhibition. V378 and A518 have hydrophobic contacts with the methyl groups and the phenyl ring, respectively; therefore, their modification to amino acids with longer chains, such as methionine and leucine, would sterically affect the binding site and the binding mode of compound **10**. F495 is a residue that has hydrophobic contacts as well as aromatic interactions with **10**, so changing it to valine or leucine would prevent aromatic interactions and induce steric changes in the binding site.

To verify the in silico studies, plasmids with mutations in glycine (G351P), valine (V378M), and phenylalanine (F495L) were transfected into HEK293 cells. TLR8-mediated activity was observed only in the F495L mutation, while other mutations, as well as dual or triple combinations, showed no activity when stimulated with the agonist TL8-506 (Figure 6A). This indicates that G351 and V378 are critical amino acids for TLR8 ligand binding. Next, we focused on the F495L mutation to determine whether compound **10** could fit into the TLR8 binding cavity. TLR8 activation, triggered by the well-characterized agonists CL075 and TL8-506, was reduced by compound **10** and Enpatoran (Figure 6B). Both agonists are known to bind to TLR8 receptor binding site, as demonstrated by crystal structures.^49^ Since Enpatoran is also characterized by a crystal structure,^21^ this suggests that compound **10** binds to the same pocket in TLR8 as the two agonists and Enpatoran. This, together with our predicted modeling studies, indicates that TL8-506 and compound **10** compete for binding to the same receptor binding site. To further determine the binding mode, Schild analysis of both compounds in hTLR8-HEK293 reporter cells was performed. Compound **10** was able to shift the concentration–response curve parallel to the right (Figure 6C). The corresponding Schild plot was linear (Figure 6D). This is supported by significantly higher EC_50_ values and unchanged *E*_max_ values of TL8-506 in the presence of compound **10** (Table S6), indicating competitive antagonism.

**Figure 6:**
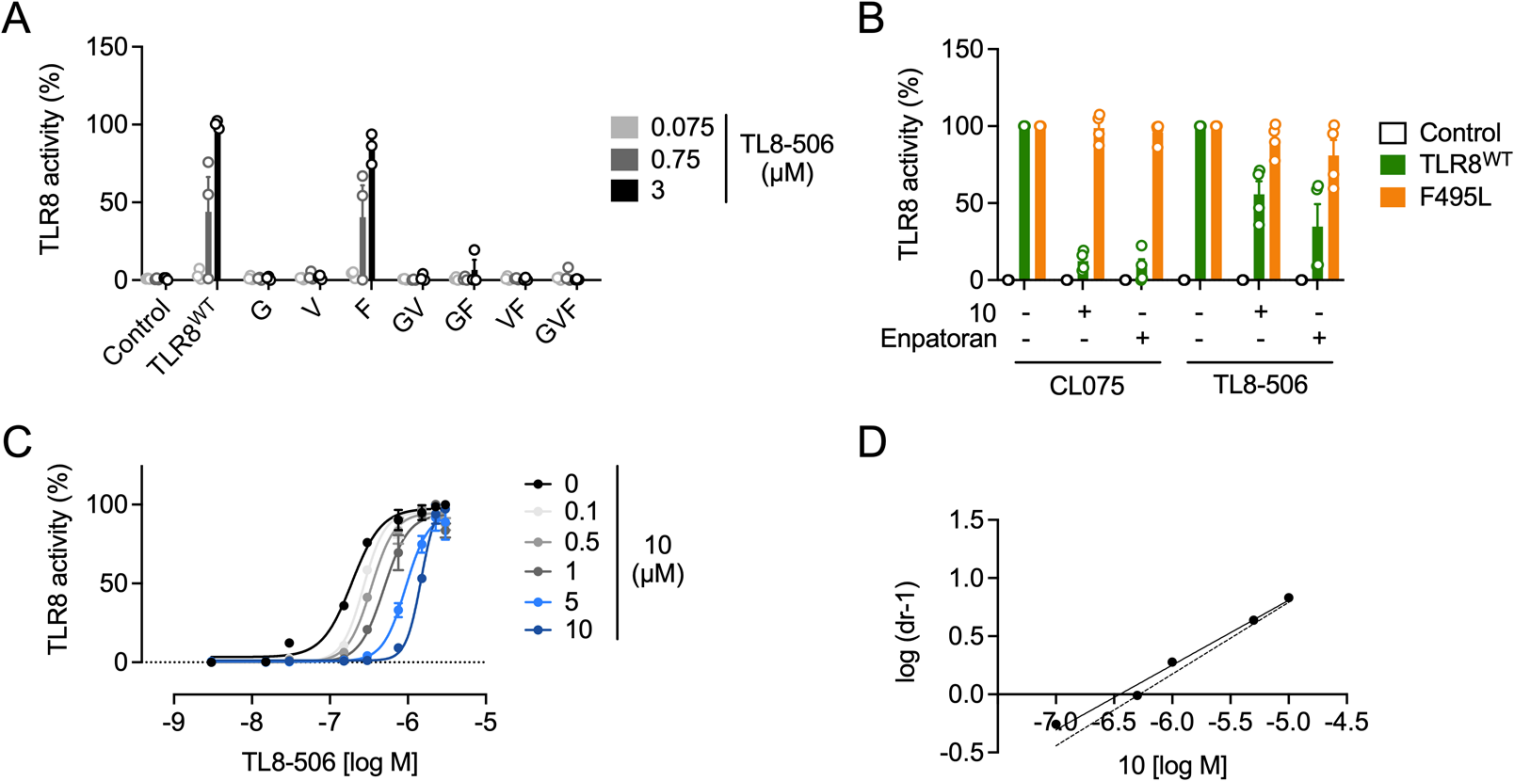
Compound **10** is a competitive antagonist of TL8-506. (A) HEK293 reporter control cells were transfected with mutant (G = Glycin351, V = Valin378, F = Phenylalanine495; GV, GF, VF, and GVF = dual or triple mutations of amino acids) or wildtype TLR8 plasmid (TLR8^WT^). Cells were stimulated with indicated concentrations of TL8-506 for 24 h. Supernatants were analyzed for TLR8-mediated NF-κB activation by SEAP reporter assay using QuantiBlue (OD_620_) and normalized to TL8-506. Mean + SEM (*n* = 3). (B) HEK293 reporter control cells were transfected with mutant or wildtype TLR8 plasmid. Cells were preincubated with either compound **10** (2 μM) or Enpatoran (1 μM) for 1 h and then stimulated with TL8-506 (3 μM) or CL075 (4 μM) for 24 h. Supernatants were analyzed for TLR8-mediated NF-κB activation by the SEAP reporter assay using QuantiBlue (OD_620_) and normalized to TL8-506 or CL075, respectively. Mean + SEM (*n* = 4). (C) hTLR8-HEK293 reporter cells were preincubated with indicated concentrations of compound **10** for 1 h and stimulated with increasing concentrations of TL8-506 for 24 h. Supernatants were analyzed for TLR8-mediated NF-κB activation by the SEAP reporter assay using QuantiBlue (OD_620_). Calculated pharmacological parameters of the concentration–effect curves are depicted in Table S6. (D) Schild plot of (C). The lines show linear regression, with Schild slopes unconstrained (solid) or constrained to unity (dashed) (*n* = 3).

Additional in silico studies were performed with compound **10** to explain the change of activity with the mutated HEK293 cells. The three main residues G351, V378, and F495* were separately mutated, and molecular docking studies were performed (Figure 7A). The G351P affects the backbone of residue 351. This leads to P351 not being able to establish a hydrogen bond with compound **10**. Consequently, we hypothesized that compound **10** would show no activity against the G351P mutant (Figure 7B,C). The V378 M mutation increases the size of the side chain of residue 378, potentially obstructing compound **10** from assessing the TLR8 binding site. Thus, we hypothesized that compound **10** would be inactive in the presence of this mutation (Figure 7D,E). Conversely, the mutation F495L decreases the size of residue 495, similarly sterically affecting the TLR8 binding site as observed with the V378 M mutation. However, the mutation to the residue L495* does not hinder compound **10** from binding in the molecular docking experiment, allowing for a binding pose of compound **10** to be identified with the F495L mutation (Figure 7F,G).

**Figure 7:**
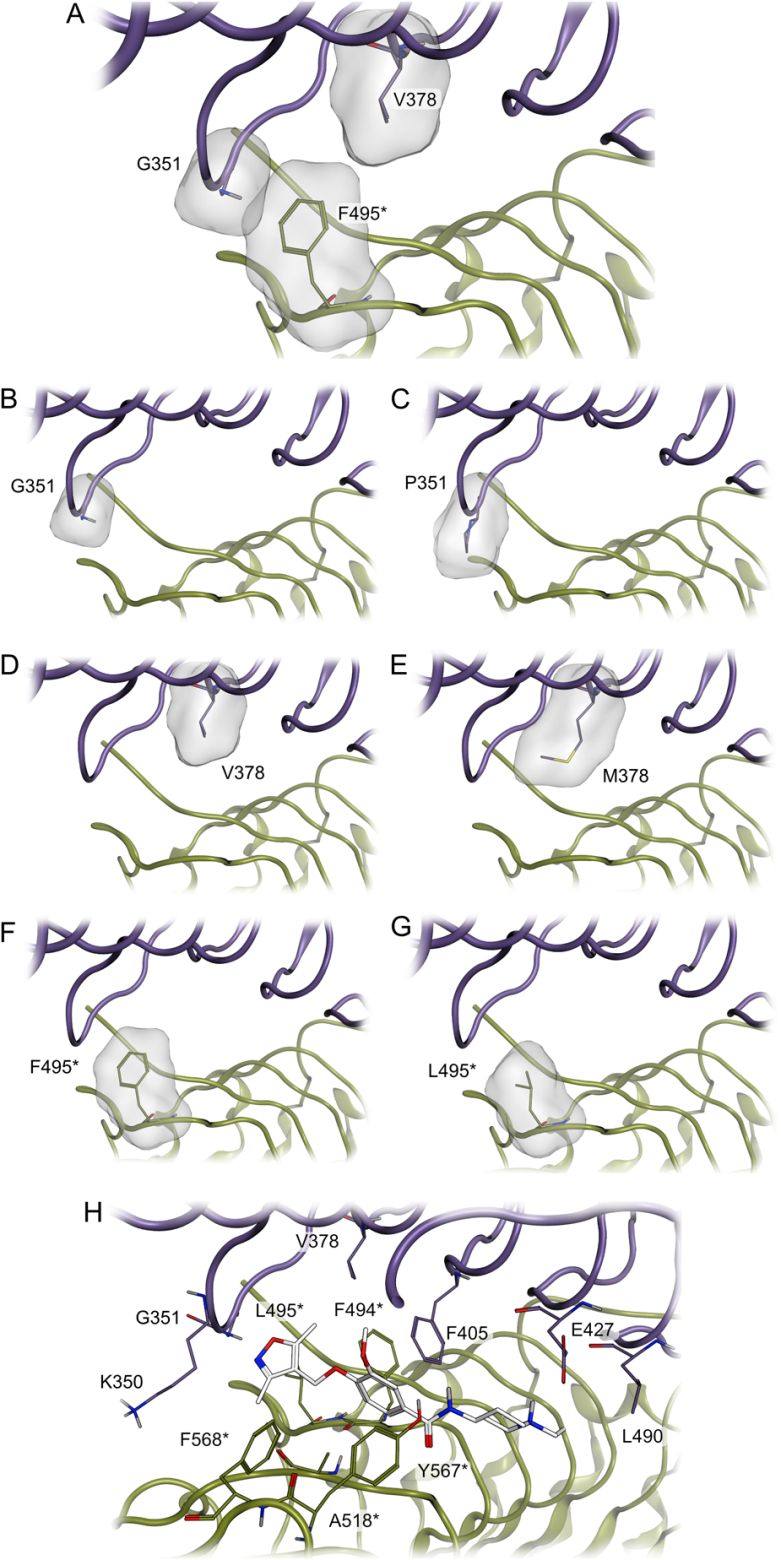
Illustration of the mutation studies of TLR8 and compound **10** binding mode with F495L mutation. TLR8 binding site interface with the highlighted surface shape of (A) residues G351, V378, and F495*; (B) residue G351 and (C) mutated residue P351; (D) residue V378 and (E) mutated residue M378; (F) residue F495* and (G) mutated residue L495*. (H) TLR8 binding site interface with the hypothetical binding pose of compound **10** with the F495L mutation. The asterisk denotes the chain B. Color code: white atoms, compound **10**; purple and dark green ribbons, TLR8 homodimers; gray surface, TLR8 protein surface.

The docking pose of compound **10** with TLR8 mutation F495L shows a hydrogen bond interaction with G351 in addition to an ionic interaction with E427. The hydrophobic interactions are identical to the interactions present in the wild-type TLR8 with the methyl groups displaying hydrophobic interactions with Y348, K350, V378, F494*, L495*, and F568*. The phenyl ring shows hydrophobic contacts with the side chains of I403, F405, A518*, and Y567*. The change of F495* to leucine disrupts an aromatic interaction between compound **10** and residue 495* (Figure 7H).

## Discussion and Conclusions

Since the identification of the first small-molecule TLR8 antagonist,^15^ little progress has been made for the exploration of selective antagonists. Given the crucial role of TLR8 in inflammatory and autoimmune diseases, the development of selective and bioavailable antagonists holds significant therapeutic potential. However, this poses a challenge, as there is currently no model to adequately address TLR8 signaling in mice, where the receptor appears to be nonfunctional.^50^

In this study, we aimed to discover and characterize novel, potent, and selective TLR8 antagonists, with solubility and bioactivity profiles that have not been previously documented.

We performed a virtual screening campaign that involved generating a 3D pharmacophore based on available crystal structures. This pharmacophore was then used for a primary virtual screening, followed by molecular docking, which led to the identification of novel compounds. These compounds contain an isoxazole scaffold that forms hydrogen bonds with the backbone of Gly351, ultimately inhibiting TLR8. The compounds also contain an aliphatic amine, which is responsible for a key ionic interaction with the Glu427 residue. The SAR studies revealed that the length of the substitute at the para position of the phenyl ring influences the activity of the compounds, which is mainly due to the hydrophobic interaction with the Leu490 residue, which is shown to be present in the docking poses of compounds that display higher activity. The most optimal substituent is *N*-ethylpiperidine, attached to the phenyl ring via an amide group. It was also observed that the presence of a methoxy group at the phenyl ring reduces the activity of some compounds. This reduction was attributed to the methoxy group pushing the phenyl ring further from the Ala518 residue, thereby losing the hydrophobic interaction between the phenyl ring and Ala518, which in turn affects the activity of the compounds. Consequently, removing the methoxy group is preferred for higher potency in *N*-alkylated piperidines. Additionally, the size of the ring is important, with piperidine being favored over pyrrolidine.

Among 12 isoxazole analogues selected based on SAR studies and biologically screened, along with an additional 26 analogues synthesized to explore the chemical space within the TLR8 binding cavity, two compounds showed significant, concentration-dependent, and highly selective inhibition of TLR8-mediated signaling in HEK293 reporter cells overexpressing TLR8, with IC_50_ values in the nanomolar range. Compound **10** and **12** showed significant reduction of cytokines in various cell lines and human primary cells without affecting responses of other TLRs, particularly TLR7.

To demonstrate that compound **10** effectively inhibits TLR8-mediated pathways in the innate immune system, we evaluated its effect on TLR8 signaling and key signaling proteins using a panel of end point assays and real-time analysis with label-free optical biosensor technology. Co-immunoprecipitation confirmed that compound **10** inhibited TLR8 activation by reducing MyD88 recruitment and inhibited the activation of NF-κB and IRF pathways. Furthermore, our findings suggest that TL8-506 as TLR8 activator can be used for priming in the activation of the inflammasome pathway. We demonstrate that compound **10** reduces IL-1β secretion when incubated together with a TLR8 agonist and either nigericin or ATP. This finding highlights the possibility in treating autoimmune diseases such as RA,^51^ which warrants further investigation.

Since the exploration of TLR8, the existence of at least two highly conserved binding sites for this receptor has been reported, one for ssRNA and small chemical ligands like TL8-506 or CL075, while the second site binds preferably to dinucleotide UG.^6,49^ An additional binding site has been proposed, which is only formed during the resting state of TLR8 homodimer.^15^ We hypothesized that our antagonist binds to this specific binding pocket. A SAR-based binding approach, combined with mutations in the TLR8 binding cavity, confirmed the ligand–receptor interaction within this pocket. We further addressed the mechanism of antagonism of compound **10**. CuCPT9a, highly potent TLR8 antagonist, has been reported to bind to the TLR8 ectodomain, as demonstrated by crystal structure analysis and ITC; however, the type of antagonism has not been experimentally confirmed.^15^ In the Schild analysis, a parallel shift for compound **10** was observed, indicating that the analyzed parameters are most consistent with competitive antagonism. Although our study did not employ a direct binding assay, a potential limitation, our comprehensive methodological approach, combining pharmacological and in silico binding exploration, suggests that compound **10** binds within the TLR8 binding cavity.

Our findings provide strong evidence that compound **10** is a potent and highly bioavailable antagonist for TLR8. Overall, our study significantly advances our understanding of the mechanisms involved in designing and evolving new TLR8 antagonists that can regulate cytokine secretion and serve as therapeutic candidates for clinical applications.

## Experimental Section

### Pharmacophore Generation

The crystal structures of known TLR8 antagonists^18,21,24–27^ were each separately loaded into LigandScout 4.4.^30^ A structure-based 3D pharmacophore^31–33^ for one of each cocrystallized ligand was generated. The 3D pharmacophores were overlapped with the reference pharmacophore of PDB ID: 7RC9 ^52^ and the features, which were present in more than four of the overlapped 3D pharmacophores, were retained.

### Pharmacophore Validation

TLR8 antagonists were retrieved from ChEMBL^34–36^ and categorized into actives consisting of compounds with IC_50_ values below 1 μM and inactives for pharmacophore validation in addition to a set of decoys based upon actives generated in the DUD-E Decoys webpage.^52^ The second validation set contained compounds with an IC_50_ value below 50 nM as actives with the previously used set of inactives. DUD-E Decoys were generated for the compounds with an IC_50_ value below 50 nM. The validation of the pharmacophore model and ROC (receiver operating characteristic) curves generation was performed in Ligandscout 4.4.^30^

### Pharmacophore Virtual Screening

The 3D pharmacophore was used for a virtual screening campaign of the Enamine Screening collection database (version 2022, retrieved from enamine.com) consisting of 2.7 million compounds. The compounds protonation states were directly taken over from the Enamine screening collection and the salt remover module from RDKit^53^ was applied to the compounds. The library was prepared using idbgen implemented in Ligandscout 4.4 ^30^ with the settings generating 25 conformations for each ligand with a minimal surface accessibility threshold for a hydrophobicity score of 0.25. The virtual screening was performed using iScreen of LigandScout 4.4 with the default settings that consist of a minimum number of required features of 3 with 0 allowed features to omit. The scoring function used in iScreen was the absolute scoring function.

### Protein Structure Preparation

The protein structure of the human TLR8 with a cocrystallized ligand fulfilling the 3D pharmacophore used for virtual screening with the highest resolution of 2.76 Å (PDB ID: 7RC9)^27^ was prepared using MOE 2022 (Chemical Computing Group, Montreal, Canada). The crystallographic waters and buffer additives were removed, while the ligand was retained. The missing side chain modeling and capping was performed using the Structure Preparation utility. The protein was protonated using the Protonate 3D function^54^ at pH 7 and temperature 300 K.

### Molecular Docking

The obtained hits were docked into the prepared crystal structure of 7RC9 ^27^ using GOLD 5.8.2 ^48^ with the binding site being defined with the radius of 6 Å around the cocrystallized ligand. For the two-step consensus molecular docking protocol, 25 genetic algorithm runs per molecule were conducted using the ChemPLP scoring function.^55^ The docking poses were selected by fulfillment of the interaction points in the respective 3D pharmacophore, consisting of a hydrogen bond acceptor, three hydrophobic features, and a positive ionizable. The selected docking poses were minimized with the Merck molecular force field 94 (MMFF94).^56^ Compounds were selected from the minimized docking poses by visual inspection according to the fulfillment of the interaction points in the respective 3D pharmacophores in addition to conformational and structural sanity, resulting in 92 selected compounds. Subsequently, the selected compounds underwent a consecutive docking with GoldScore scoring function^37^ and ChemScore scoring function.^48^ Finally, all docking poses underwent the docking pose selection, docking pose minimization, and visual inspection with the selection criteria described above.

For the SAR in silico studies, the 25 genetic algorithm runs per molecule were conducted using the ChemPLP scoring function.^55^ All docking poses underwent the docking pose selection, docking pose minimization, and visual inspection with the selection criteria described above.

### Mutation Studies

For the mutation studies the crystal structure PDB ID: 5WYZ^48^ was used. Point mutations G351P, V378L, F495V, and A518L were generated with Protein builder tool in MOE 2022 (Chemical Computing Group, Montreal, Canada) with the selection of the mutation residue followed by a side chain minimization with backbone tethering with the cocrystallized ligand being inactive, thus not affecting the mutation. The cocrystallized ligand was redocked into the crystal structure with the mutations with GOLD 5.8.2.^48^ The binding site was defined with the radius of 6 Å around the cocrystallized ligand in the crystal structure with the mutation. For the molecular docking, 15 genetic algorithm runs per molecule were conducted using the GoldScore scoring function.^37^ The docking poses were minimized with the Merck molecular force field 94 (MMFF94).^56^ The final docking pose was selected by visual inspection according to fulfillment of conformational sanity.

The crystal structure PDB ID: 7RC9 ^30^ was used for the molecular docking studies with compound **10**. The mutations G351P, V378L, and F495L were generated with Protein builder tool in MOE 2022 (Chemical Computing Group, Montreal, Canada) with the selection of the mutation residue followed by a side chain minimization with backbone tethering with the cocrystallized ligand being inactive, thus not affecting the mutation. Compound **10** was docked into the crystal structure with the mutations with GOLD 5.8.2.^48^ The binding site was defined with the radius of 10 Å around the cocrystallized ligand in the crystal structure with the mutation. For the molecular docking, 25 genetic algorithm runs per molecule were conducted using the ChemPLP scoring function.^55^ The docking poses were minimized with the Merck molecular force field 94 (MMFF94).^56^ The final docking pose was selected by visual inspection according to the fulfillment of conformational sanity.

### Molecular Dynamics Simulations

The molecular dynamics (MD) simulations were performed on RTX4090 and RTX3090 graphics processing units (NVIDIA Corporation, Santa Clara). The simulations for the TLR8 complex with **10** were prepared for molecular dynamics (MD) simulations using Maestro 11.7 (Schrödinger, LLC, New York, USA). The hydrogen bond network in both systems was optimized at pH 7.0 and temperature 300 K. The complex was placed in a TIP3P^57^ water box with a 10 Å padding distance to the protein surface. The system was isotonized with 0.15 M NaCl. The system was parametrized using the OPLS 2005 force field^58^ and relaxed using the default Desmond protocol. MD simulations were carried out with a constant number of particles, pressure, and temperature (*NPT* ensemble). During the main MD simulation, the constant temperature of 300 K was held using the Nose–Hoover thermostat.^59,60^ The constant pressure of 1.01325 bar was preserved using the Martyna-Tobias-Klein method.^61^ Three replicas with a duration of 50 ns each were simulated, each generating 1000 frames. The replicas were concatenated using VMD^62^ and the combined trajectory of the protein and ligand was analyzed using the DynophoreApp in LigandScout 4.4.^30^

### Virtual Screening Compound Purity Testing

The purification of virtual screening compounds **1**-**12** with HPLC was performed using an Agilent 1290 Infinity system with a binary pump, autosampler, column compartment, and Agilent 1260 DAD VL + detector. Mass spectrometric detection was performed using an Agilent 6130B Single Quadrupole MS. Compound purification was achieved with an Agilent Poroshell C18 column (100 × 2.1 mm, 2.7 μm particle size) at 30 °C with a flow rate of 0.400 mL/min. The mobile phase consisted of solvent A (water with 0.1% formic acid) and solvent B (acetonitrile with 0.1% formic acid). The gradient for the purification started with 5% solvent B, held for 1 min, and followed by a linear increase to 95% solvent B over 8 min. The gradient was maintained at 95% solvent B for 2 min before returning to 5% solvent B in 0.5 min. The total runtime was 11 min. The injection volume was 0.5 μL. The DAD signals were monitored at 254, 210, and 220 nm, with a scan rate of 40 Hz. MS detection was performed in both positive and negative ion modes, scanning a mass range of *m*/*z* 50–700. All compounds were >95% pure by HPLC analysis (Figure S12). ^1^H NMR spectra for purchased active compounds **1** and **10**-**12** are provided in Figure S13.

### General Methods and Analytical Data for Synthesized Compounds

Reagents and solvents were purchased from commercial sources (e.g., BLDpharm, Sigma-Aldrich, Acros Organics, Apollo Scientific, Fluorochem, Enamine, and TCI). Reactions were monitored by thin-layer chromatography on silica gel plates (Merck DC Fertigplatten Kieselgel 60 GF254) and visualized under UV light or stained with the appropriate staining reagents. Flash column chromatography was performed on silica gel 60 (mesh size 70–230; Merck) using the indicated solvents. Yields are for the purified products and were not optimized. 1H and 13C NMR spectra were recorded at 295 K in CDCl_3_, DMSO-*d*_6_ or acetone*-d*_6_ (Avance III NMR spectrometer; Bruker, MA, USA) using a decoupling inverse 1H probe (Broadband). The coupling constants (*J*) are given in Hz, and the splitting patterns are designated as follows: s, singlet; bs, broad singlet; d, doublet; t, triplet; q, quartet; dd, doublet of doublets; dt, doublet of triplets; m, multiplet. Mass spectra (Expression CMS mass spectrometer; Advion, NY, USA) and high-resolution mass measurements (Exactive Plus Orbitrap mass spectrometer; Thermo Fischer Scientific, MA, USA) were performed at the Faculty of Pharmacy, University of Ljubljana, Slovenia. HPLC analyses were performed on the Thermo Scientific UltiMate 3000 modular system (Thermo Fisher Scientific Inc.) equipped with a quaternary pump and a multiple wavelength detector. An ACQUITY UPLC HSS C18 column (2.1 × 50 mm; 1.8 μm), thermostated at 40 °C, was used with a flow rate of 0.4 mL/min; detection at 254 nm; and an eluent system of: A, 0.1% aqueous trifluoroacetic acid; B, acetonitrile. The following gradient was applied: 0–7 min, 5–95% B; 7–8 min, 95% B. All compounds were >95% pure by HPLC analysis. ^1^H NMR, ^13^C NMR, and HPLC traces for the synthesized active compounds are provided in Figure S14.

### General Procedure A

Methyl 4-hydroxy-3-methoxybenzoate or methyl 4-hydroxybenzoate (1 equiv) was dissolved in acetone, and then K_2_CO_3_ (1.1 equiv), 4-(chloromethyl)-3,5-dimethylisoxazole (1 equiv), and KI (cat.) were added. The reaction mixture was stirred overnight at 50 °C. The solvent was evaporated and EtOAc was added to the residue. The organic phase was washed with water (2×) and brine, dried over anhydrous Na_2_SO_4_, filtered, and evaporated under reduced pressure. The product was used in the next step without further purification.

#### Methyl 4-((3,5-Dimethylisoxazol-4-yl)­methoxy)­benzoate (**13**)

The compound was synthesized from methyl 4-hydroxybenzoate (32.86 mmol, 5.00 g), K_2_CO_3_ (36.15 mmol, 5.00 g), 4-(chloromethyl)-3,5-dimethylisoxazole (32.86 mmol, 4.78 g), and KI (cat.) according to general procedure A. Yield 53%; yellow solid; *R*_*f*_ = 0.43 (EtOAc/*n*-Hex, 2:1, v/v); ^1^H NMR (400 MHz, DMSO-*d*_6_): δ (ppm) = 2.22 (s, 3H), 2.42 (s, 3H), 3.82 (s, 3H), 5.02 (s, 2H), 6.99–7.23 (m, 2H), 7.75–8.12 (m, 2H).

#### Methyl 4-((3,5-Dimethylisoxazol-4-yl)­methoxy)-3-methoxybenzoate (**14**)

The compound was synthesized from methyl 4-hydroxy-3-methoxybenzoate (36.00 mmol, 6.56 g), K_2_CO_3_ (40 mmol, 5.53 g), 4-(chloromethyl)-3,5-dimethylisoxazole (36.00 mmol, 5.24 g) and KI (cat.) according to general procedure A. Yield 95%; white solid; *R*_*f*_ = 0.60 (EtOAc/*n*-Hex, 1:1, v/v); ^1^H NMR (400 MHz, DMSO-*d*_6_): δ (ppm) 2.22 (s, 3H), 2.41 (s, 3H), 3.81 (s, 3H), 3.83 (s, 3H), 5.00 (s, 2H), 7.21 (d, *J* = 8.5 Hz, 1H), 7.47 (d, *J* = 2.0 Hz, 1H), 7.61 (dd, *J* = 8.4, 2.0 Hz, 1H).

### General Procedure B

An appropriate benzoate (1 equiv) was dissolved in 1 M NaOH and 1,4-dioxane and stirred overnight at room temperature. Diethyl ether was added to the reaction mixture, and phases were separated. One M HCl was added to the water phase, and the product precipitated.

#### 4-((3,5-Dimethylisoxazol-4-yl)­methoxy)­benzoic Acid (**15**)

The compound was synthesized from methyl 4-((3,5-dimethylisoxazol-4-yl)­methoxy)­benzoate (17.54 mmol, 4.58 g) and 1 M NaOH according to general procedure B. Yield 90%; white solid; *R*_*f*_ = 0.19 (EtOAc/*n*-Hex, 2:1, v/v); ^1^H NMR (400 MHz, DMSO-*d*_6_): δ (ppm) = 2.21 (s, 3H), 2.42 (s, 3H), 5.00 (s, 2H), 6.96–7.23 (m, 2H), 7.71–8.03 (m, 2H), 12.67 (br s, 1H).

#### 4-((3,5-Dimethylisoxazol-4-yl)­methoxy)-3-methoxybenzoic Acid (**16**)

The compound was synthesized from methyl 4-((3,5-dimethylisoxazol-4-yl)­methoxy)-3-methoxybenzoate (36.00 mmol, 10.50 g) and 1 M NaOH according to general procedure B. Yield 78%; white solid; *R*_*f*_ = 0.21 (EtOAc/*n*-Hex, 1:1, v/v); ^1^H NMR (400 MHz, DMSO-*d*_6_): δ (ppm) = 2.22 (s, 3H), 2.40 (s, 3H), 3.80 (s, 3H), 4.99 (s, 2H), 7.18 (d, *J* = 8.5 Hz, 1H), 7.47 (d, *J* = 1.9 Hz, 1H), 7.58 (dd, *J* = 8.4, 1.9 Hz, 1H), 12.75 (br s, 1H).

### General Procedure C

An appropriate acid (1 equiv) was dissolved in anhydrous THF under an argon atmosphere, and then HATU (1.5 equiv) was added. An appropriate amine (1 equiv) and DIPEA (4 equiv) were then added to the stirred solution. The reaction mixture was stirred overnight at room temperature. EtOAc was then added and the obtained solution was washed with saturated NaHCO_3_ solution. The organic phase was dried over anhydrous Na_2_SO_4_ and filtered, and the solvent was removed under reduced pressure. The residue was purified by flash column chromatography.

#### 4-Benzylpiperazin-1-yl­(4-((3,5-Dimethylisoxazol-4-yl)­methoxy)­phenyl)­methanone (**17**)

The compound was synthesized from 4-((3,5-dimethylisoxazol-4-yl)­methoxy)­benzoic acid (0.22 mmol, 0.054 g), HATU (0.33 mmol, 0.126 g), 1-benzylpiperazine (0.22 mmol, 0.039 mL), and DIPEA (0.88 mmol, 0.153 mL) according to general procedure C. The compound was purified by flash column chromatography with EtOAc/*n*-Hex = 4/1 (v/v) as eluent. Yield 34%; white solid; *R*_*f*_ = 0.51 (EtOAc/*n*-Hex, 4:1, v/v); ^1^H NMR (400 MHz, CDCl_3_): δ (ppm) = 2.29 (s, 3H), 2.41 (s, 3H), 2.46 (br s, 4H), 3.51 (br s, 2H), 3.54 (s, 2H), 3.70 (br s, 2H), 4.80 (s, 2H), 6.92–6.96 (m, 2H), 7.27–7.35 (m, 5H), 7.37–7.41 (m, 2H); ^13^C NMR (100 MHz, CDCl_3_): δ (ppm) = 10.16, 11.19, 53.06, 59.57, 62.94, 77.22, 109.96, 114.47, 127.31, 128.35, 128.78, 129.14, 129.22, 137.57, 159.42, 159.69, 167.59, 169.99; HRMS (ESI^+^) *m*/*z* calcd for C_24_H_28_N_3_O_3_ [M + H]^+^ 406.21252; found, 406.21149; HPLC purity 100% at 254 nm (*t*_R_ = 3.373 min).

#### 4-((3,5-Dimethylisoxazol-4-yl)­methoxy)-*N*-(2-(pyrrolidin-1-yl)­ethyl)­benzamide (**18**)

The compound was synthesized from 4-((3,5-dimethylisoxazol-4-yl)­methoxy)­benzoic acid (0.61 mmol, 0.150 g), HATU (0.92 mmol, 0.348 g), 2-(pyrrolidine-1-yl)­ethan-1-amine (0.61 mmol, 0.077 mL), and DIPEA (2.44 mmol, 0.425 mL) according to general procedure C. The compound was purified by flash column chromatography with DCM/MeOH = 9/1 (v/v) as eluent. Yield 14%; yellow oil; *R*_*f*_ = 0.20 (DCM/MeOH, 9:1, v/v); ^1^H NMR (400 MHz, acetone-*d*_6_): δ (ppm) = 2.07–2.13 (m, 4H), 2.24 (s, 3H), 2.43 (s, 3H), 3.45–3.47 (m, 6H), 3.77–3.81 (m, 2H), 5.04 (s, 2H), 7.08–7.12 (m, 2H), 7.89–7.93 (m, 2H), 8.15 (br s, 1H); ^13^C NMR (100 MHz, acetone-*d*_6_): δ (ppm) = 10.06, 10.94, 23.85, 38.01, 55.55, 57.45, 60.38, 111.04, 115.45, 127.19, 130.11, 160.30, 162.33, 168.35, 169.01; HRMS (ESI^+^) *m*/*z* calcd for C_19_H_26_N_3_O_3_ [M + H]^+^ 344.19687; found, 344.19599; HPLC purity 100.00% at 254 nm (*t*_R_ = 3.030 min).

#### 4-((3,5-Dimethylisoxazol-4-yl)­methoxy)-*N*-(1-methylpyrrolidin-3-yl)­benzamide (**19**)

The compound was synthesized from 4-((3,5-dimethylisoxazol-4-yl)­methoxy)­benzoic acid (0.61 mmol, 0.150 g), HATU (0.92 mmol, 0.348 g), 1-methylpyrrolidin-3-amine (0.61 mmol, 0.061 g), and DIPEA (2.44 mmol, 0.425 mL) according to general procedure C. The compound was purified by flash column chromatography with DCM/MeOH = 9/1 (v/v) as eluent. Yield 11%; white solid; *R*_*f*_ = 0.36 (DCM/MeOH, 9:1, v/v); ^1^H NMR (400 MHz, acetone-*d*_6_): δ (ppm) = 1.73–1.82 (m, 1H), 2.24 (s, 3H), 2.26–2.30 (m, 1H), 2.32 (s, 3H), 2.35–2.41 (m, 1H), 2.43 (s, 3H), 2.57 (dd, *J* = 9.6, 4.1 Hz, 1H), 2.67–2.77 (m, 2H), 4.45–4.64 (m, 1H), 5.00 (s, 2H), 7.02–7.06 (m, 2H), 7.69 (d, *J* = 6.2 Hz, 1H), 7.89–7.93 (m, 2H); ^13^C NMR (100 MHz, acetone-*d*_6_): δ (ppm) = 10.06, 10.94, 33.11, 42.01, 50.26, 55.70, 60.27, 63.34, 111.14, 115.13, 128.60, 128.62, 129.90, 160.32, 161.75, 166.24, 166.30, 168.30; HRMS (ESI^+^) *m*/*z* calcd for C_18_H_24_N_3_O_3_ [M + H]^+^ 330.18122; found, 330.18044; HPLC purity 99.38% at 254 nm (*t*_R_ = 2.873 min).

#### 4-((3,5-Dimethylisoxazol-4-yl)­methoxy)-*N*-(4-methylpiperazin-1-yl)­benzamide (**20**)

The compound was synthesized from 4-((3,5-dimethylisoxazol-4-yl)­methoxy)­benzoic acid (0.61 mmol, 0.150 g), HATU (0.92 mmol, 0.348 g), 4-methylpiperazin-1-amine (0.61 mmol, 0.073 mL), and DIPEA (2.44 mmol, 0.425 mL) according to general procedure C. The compound was purified by flash column chromatography with DCM/MeOH = 9/1 (v/v) as eluent. Yield 20%; white solid; *R*_*f*_ = 0.27 (DCM/MeOH, 9:1, v/v); ^1^H NMR (400 MHz, acetone-*d*_6_): δ (ppm) = 2.22 (s, 3H), 2.24 (s, 3H), 2.43 (s, 3H), 2.46 (s, 4H), 2.98 (s, 4H), 5.01 (s, 2H), 7.04 (d, *J* = 8.8 Hz, 2H), 7.82 (d, *J* = 8.5 Hz, 2H), 8.45 (s, 1H); ^13^C NMR (100 MHz, acetone-*d*_6_): δ (ppm) = 10.15, 11.19, 45.70, 54.31, 55.62, 59.60, 109.84, 114.52, 126.64, 129.03, 159.65, 160.97, 164.89, 167.65; HRMS (ESI^+^) *m*/*z* calcd for C_18_H_25_N_4_O_3_ [M + H]^+^ 345.19212; found, 345.19122; HPLC purity 99.67% at 254 nm (*t*_R_ = 2.760 min).

#### (4-((3,5-Dimethylisoxazol-4-yl)­methoxy)­phenyl)­(4-(pyrrolidin-1-yl)­piperidin-1-yl)­methanone (21)

The compound was synthesized from 4-((3,5-dimethylisoxazol-4-yl)­methoxy)­benzoic acid (0.61 mmol, 0.150 g), HATU (0.92 mmol, 0.348 g), 4-(pyrrolidin-1-yl)­piperidine (0.61 mmol, 0.094 g), and DIPEA (2.44 mmol, 0.425 mL) according to general procedure C. The compound was purified by flash column chromatography with DCM/MeOH = 9/1 (v/v) as eluent. Yield 26%; yellow oil; *R*_*f*_ = 0.31 (DCM/MeOH, 9:1, v/v); ^1^H NMR (400 MHz, CDCl_3_): δ (ppm) = 1.50–1.62 (m, 2H), 1.80–1.85 (m, 4H), 2.05 (br s, 4H), 2.28 (s, 3H), 2.34–2.40 (m, 1H), 2.41 (s, 3H), 2.60–2.70 (m, 4H), 2.97 (br s, 2H), 4.81 (s, 2H), 6.93–6.95 (m, 2H), 7.36–7.38 (m, 2H); ^13^C NMR (100 MHz, CDCl_3_): δ (ppm) = 10.28, 11.30, 23.33, 31.41, 51.64, 59.71, 61.82, 110.12, 114.68, 129.05, 129.11, 159.54, 159.83, 167.72, 170.21; HRMS (ESI^+^) *m*/*z* calcd for C_22_H_30_N_3_O_3_ [M + H]^+^ 384.22817; found, 384.22718; HPLC purity 100.00% at 254 nm (*t*_R_ = 3.047 min).

#### (4-Benzylpiperazin-1-yl)­(4-((3,5-dimethylisoxazol-4-yl)­methoxy)-3-methoxyphenyl)­methanone (**22**)

The compound was synthesized from 4-((3,5-dimethylisoxazol-4-yl)­methoxy)-3-methoxybenzoic acid (0.54 mmol, 0.150 g), HATU (0.81 mmol, 0.308 g), 1-benzylpiperazine (0.54 mmol, 0.094 mL), and DIPEA (2.16 mmol, 0.376 mL) according to general procedure C. The compound was purified by flash column chromatography with EtOAc/*n*-Hex = 4/1 (v/v) as eluent. Yield 14%; white solid; *R*_*f*_ = 0.25 (EtOAc/*n*-Hex, 4:1, v/v); ^1^H NMR (400 MHz, CDCl_3_): δ (ppm) = 2.30 (s, 3H), 2.38 (s, 3H), 2.46 (br s, 4H), 3.52 (br s, 2H), 3.54 (s, 2H), 3.74 (br s, 2H), 3.86 (s, 3H), 4.85 (s, 2H), 6.89 (d, *J* = 8.2 Hz, 1H), 6.94 (dd, *J* = 8.1, 1.8 Hz, 1H), 7.00 (d, *J* = 1.8 Hz, 1H), 7.28–7.35 (m, 5H); ^13^C NMR (100 MHz, CDCl_3_): δ (ppm) = 10.14, 11.16, 53.14, 55.95, 61.05, 62.93, 110.13, 111.52, 114.35, 119.81, 127.32, 128.35, 129.13, 129.73, 137.55, 148.71, 150.20, 159.81, 167.68, 169.92; HRMS (ESI^+^) *m*/*z* calcd for C_25_H_30_N_3_O_4_ [M + H]^+^ 436.22308; found, 436.22217; HPLC purity 98.60% at 254 nm (*t*_R_ = 3.333 min).

#### 4-((3,5-Dimethylisoxazol-4-yl)­methoxy)-3-methoxy-*N*-(4-methylpiperazin-1-yl)­benzamide (**23**)

The compound was synthesized from 4-((3,5-dimethylisoxazol-4-yl)­methoxy)-3-methoxybenzoic acid (1.44 mmol, 0.400 g), HATU (2.16 mmol, 0.821 g), 4-methylpiperazin-1-amine (1.44 mmol, 0.121 mL), and DIPEA (5.76 mmol, 1.00 mL) according to general procedure C. The compound was purified by flash column chromatography with DCM/MeOH = 9/1 (v/v) as eluent. Yield 30%; white solid; *R*_*f*_ = 0.34 (DCM/MeOH, 9:1, v/v); ^1^H NMR (400 MHz, CDCl_3_): δ (ppm) = 2.30 (s, 3H), 2.34 (s, 3H), 2.39 (s, 3H), 2.65 (br s, 4H), 2.97 (br s, 4H), 3.90 (s, 3H), 4.88 (s, 2H), 6.70 (br s, 1H), 6.90 (d, *J* = 8.3 Hz, 1H), 7.19–7.24 (m, 1H), 7.39 (br s, 1H); ^13^C NMR (100 MHz, CDCl_3_): δ (ppm) = 10.15, 11.18, 45.75, 54.33, 55.69, 56.07, 60.99, 109.98, 111.52, 114.00, 119.09, 127.79, 150.37, 159.77, 165.04, 167.73; HRMS (ESI^+^) *m*/*z* calcd for C_19_H_27_N_4_O_4_ [M + H]^+^ 375.20268; found, 375.20167; HPLC purity 100.00% at 254 nm (*t*_R_ = 2.727 min).

#### (4-((3,5-Dimethylisoxazol-4-yl)­methoxy)-3-methoxyphenyl)­(4-(pyrrolidin-1-yl)­piperidin-1-yl)­methanone (**24**)

The compound was synthesized from 4-((3,5-dimethylisoxazol-4-yl)­methoxy)-3-methoxybenzoic acid (1.00 mmol, 0.277 g), HATU (1.50 mmol, 0.570 g), 4-(pyrrolidin-1-yl)­piperidine (1.00 mmol, 0.154 g), and DIPEA (4.00 mmol, 0.697 mL) according to general procedure C. The compound was purified by flash column chromatography with DCM/MeOH = 9/1 (v/v) as eluent. Yield 56%; white solid; *R*_*f*_ = 0.33 (DCM/MeOH, 9:1, v/v); ^1^H NMR (400 MHz, CDCl_3_): δ (ppm) = 1.51–1.62 (m, 2H), 1.83–1.87 (m, 4H), 1.97 (br s, 4H), 2.30 (s, 3H), 2.39 (s, 3H), 2.40–2.48 (m, 1H), 2.63–2.75 (m, 4H), 2.98 (br s, 2H), 3.85 (s, 3H), 4.86 (s, 2H), 6.91 (d, *J* = 8.2 Hz, 1H), 6.94 (dd, *J* = 8.1, 1.7 Hz, 1H), 6.98 (d, *J* = 1.7 Hz, 1H); ^13^C NMR (100 MHz, acetone-*d*_6_): δ (ppm) = 10.04, 10.89, 23.96, 31.79, 51.94, 56.23, 61.38, 62.20, 111.40, 112.29, 115.52, 120.58, 131.25, 149.70, 151.00, 160.46, 168.32, 169.95; HRMS (ESI^+^) *m*/*z* calcd for C_23_H_32_N_3_O_4_ [M + H]^+^ 414.23873; found, 414.23751; HPLC purity 100.00% at 254 nm (*t*_R_ = 2.993 min).

#### (4-((3,5-Dimethylisoxazol-4-yl)­methoxy)-3-methoxyphenyl)­(5-methylhexahydropyrrolo­[3,4-*c*]­pyrrol-2­(1*H*)-yl)­methanone (**25**)

The compound was synthesized from 4-((3,5-dimethylisoxazol-4-yl)­methoxy)-3-methoxybenzoic acid (0.500 mmol, 0.139 g), HATU (0.75 mmol, 0.285 g), 2-methyloctahydropyrrolo­[3,4-*c*]­pyrrole (0.500 mmol, 0.063 g), and DIPEA (2.00 mmol, 0.348 mL) according to general procedure C. The compound was purified by flash column chromatography with DCM/MeOH = 9/1 (v/v) as eluent. Yield 49%; yellow oil; *R*_*f*_ = 0.22 (DCM/MeOH, 9:1, v/v); ^1^H NMR (400 MHz, acetone-*d*_6_): δ (ppm) = 2.26 (s, 3H), 2.37 (s, 3H), 2.39 (s, 3H), 2.52–2.69 (m, 4H), 2.85–2.93 (m, 2H), 3.45–3.54 (m, 2H), 3.74–3.82 (m, 2H), 3.83 (s, 3H), 4.97 (s, 2H), 7.08 (d, *J* = 1.1 Hz, 2H), 7.10–7.14 (m, 1H); ^13^C NMR (100 MHz, acetone-*d*_6_): δ (ppm) = 10.04, 10.89, 40.16, 41.74, 49.70, 56.21, 61.34, 62.85, 111.39, 112.64, 115.20, 121.13, 131.94, 149.92, 150.77, 160.46, 168.32, 168.76; HRMS (ESI^+^) *m*/*z* calcd for C_21_H_28_N_3_O_4_ [M + H]^+^ 386.20743; found, 386.20639; HPLC purity 100.00% at 254 nm (*t*_R_ = 2.740 min).

#### [1,4′-Bipiperidin]-1′-yl­(4-((3,5-dimethylisoxazol-4-yl)­methoxy)-3-methoxyphenyl)­methanone (**26**)

The compound was synthesized from 4-((3,5-dimethylisoxazol-4-yl)­methoxy)-3-methoxybenzoic acid (1.44 mmol, 0.400 g), HATU (2.16 mmol, 0.821 g), 1,4′-bipiperidine (1.44 mmol, 0.243 g), and DIPEA (5.76 mmol, 1.00 mL) according to general procedure C. The compound was purified by flash column chromatography with DCM/MeOH = 9/1 (v/v) as eluent. Yield 27%; white solid; *R*_*f*_ = 0.25 (DCM/MeOH, 9:1, v/v); ^1^H NMR (400 MHz, acetone-*d*_6_): δ (ppm) = 1.59–1.64 (m, 2H), 1.71–1.86 (m, 6H), 2.12 (bd, *J* = 12.2 Hz, 2H), 2.26 (s, 3H), 2.39 (s, 3H), 3.13–3.25 (m, 5H), 3.33–3.39 (m, 4H), 3.83 (s, 3H), 4.97 (s, 2H), 6.99 (dd, *J* = 8.1, 1.9 Hz, 1H), 7.03 (d, *J* = 1.9 Hz, 1H), 7.09 (d, *J* = 8.2 Hz, 1H); ^13^C NMR (100 MHz, acetone-*d*_6_): δ (ppm) = 10.02, 10.87, 23.37, 25.08, 27.75, 46.40, 51.04, 56.25, 61.24, 64.29, 111.29, 112.05, 115.38, 120.60, 130.36, 149.86, 150.92, 160.44, 168.31, 170.27; HRMS (ESI^+^) *m*/*z* calcd for C_24_H_34_N_3_O_4_ [M + H]^+^ 428.25438; found, 428.25360; HPLC purity 100.00% at 254 nm (*t*_R_ = 3.127 min).

#### 4-((3,5-Dimethylisoxazol-4-yl)­methoxy)-3-methoxy-*N*-(1-methylpyrrolidin-3-yl)­benzamide (**27**)

The compound was synthesized from 4-((3,5-dimethylisoxazol-4-yl)­methoxy)-3-methoxybenzoic acid (1.08 mmol, 0.300 g), HATU (1.62 mmol, 0.616 g), 1-methylpyrrolidin-3-amine (1.08 mmol, 0.112 mL), and DIPEA (4.32 mmol, 0.753 mL) according to general procedure C. The compound was purified by flash column chromatography with DCM/MeOH = 9/1 (v/v) as eluent. Yield 39%; white solid; *R*_*f*_ = 0.33 (DCM/MeOH, 9:1, v/v); ^1^H NMR (400 MHz, methanol-*d*_4_): δ (ppm) = 1.87–1.95 (m, 1H), 2.28 (s, 3H), 2.35–2.44 (m, 1H), 2.39 (s, 3H), 2.48 (s, 3H), 2.62–2.68 (m, 1H), 2.74 (dd, *J* = 10.4, 4.7 Hz, 1H), 2.91–2.98 (m, 2H), 3.88 (s, 3H), 4.53–4.59 (m, 1H), 4.97 (s, 2H), 7.09–7.11 (m, 1H), 7.45–7.48 (m, 2H), 1H from NH is exchanged; ^13^C NMR (100 MHz, methanol-*d*_4_): δ (ppm) = 9.94, 10.80, 32.55, 42.09, 50.97, 56.05, 56.44, 61.67, 62.82, 111.80, 112.28, 115.57, 121.72, 129.08, 151.32, 151.96, 161.45, 169.41, 169.56; HRMS (ESI^+^) *m*/*z* calcd for C_19_H_26_N_3_O_4_ [M + H]^+^ 360.19178; found, 360.19084; HPLC purity 97.63% at 254 nm (*t*_R_ = 2.850 min).

#### 4-((3,5-Dimethylisoxazol-4-yl)­methoxy)-*N*-(1-isopropylpiperidin-4-yl)-3-methoxybenzamide (**28**)

The compound was synthesized from 4-((3,5-dimethylisoxazol-4-yl)­methoxy)-3-methoxybenzoic acid (0.54 mmol, 0.150 g), HATU (0.81 mmol, 0.308 g), 1-isopropylpiperidin-4-amine (0.54 mmol, 0.077 g), and DIPEA (2.16 mmol, 0.376 mL) according to general procedure C. The compound was purified by flash column chromatography with DCM/MeOH = 9/1 (v/v) as eluent. Yield 33%; white solid; *R*_*f*_ = 0.20 (DCM/MeOH, 9:1, v/v); ^1^H NMR (400 MHz, CDCl_3_): δ (ppm) = 1.06 (d, *J* = 6.6 Hz, 6H), 1.49–1.56 (m, 2H), 2.01–2.15 (m, 2H), 2.30 (s, 3H), 2.34 (dd, *J* = 11.5, 2.2 Hz, 2H), 2.38 (s, 3H), 2.71–2.81 (m, 1H), 2.83–2.93 (m, 2H), 3.91 (s, 3H), 3.92–4.04 (m, 1H), 4.88 (s, 2H), 5.87 (d, *J* = 8.3 Hz, 1H), 6.90 (d, *J* = 8.3 Hz, 1H), 7.19 (dd, *J* = 8.3, 2.0 Hz, 1H), 7.43 (d, *J* = 2.0 Hz, 1H); ^13^C NMR (100 MHz, CDCl_3_): δ (ppm) = 10.15, 11.17, 18.42, 32.69, 47.39, 47.57, 54.58, 56.04, 61.03, 110.02, 111.39, 114.08, 118.76, 128.97, 150.15, 150.25, 159.78, 166.23, 167.73; HRMS (ESI^+^) *m*/*z* calcd for C_22_H_32_N_3_O_4_ [M + H]^+^ 402.23873; found, 402.23742; HPLC purity 98.91% at 254 nm (*t*_R_ = 2.803 min).

#### 4-((3,5-Dimethylisoxazol-4-yl)­methoxy)-*N*-(1-ethylpyrrolidin-3-yl)-3-methoxybenzamide (**29**)

The compound was synthesized from 4-((3,5-dimethylisoxazol-4-yl)­methoxy)-3-methoxybenzoic acid (0.54 mmol, 0.150 g), HATU (0.81 mmol, 0.308 g), 1-ethylpyrrolidin-3-amine dihydrochloride (0.54 mmol, 0.101 g), and DIPEA (2.16 mmol, 0.376 mL) according to general procedure C. The compound was purified by flash column chromatography with DCM/MeOH = 9/1 (v/v) as eluent. Yield 10%; colorless oil; *R*_*f*_ = 0.22 (DCM/MeOH, 9:1, v/v); ^1^H NMR (400 MHz, CDCl_3_): δ (ppm) = 1.32 (t, *J* = 7.3 Hz, 3H), 1.97–2.11 (m, 1H), 2.30 (s, 3H), 2.38 (s, 3H), 2.55–2.65 (m, 1H), 2.75–2.82 (m, 1H), 2.78 (q, *J* = 7.3 Hz, 2H), 3.03–3.11 (m, 1H), 3.28–3.34 (m, 1H), 3.46–3.54 (m, 1H), 3.90 (s, 3H), 4.63–4.72 (m, 1H), 4.88 (s, 2H), 6.93 (d, *J* = 8.4 Hz, 1H), 7.18–7.26 (m, 1H), 7.24 (br s, 3H), 7.37 (dd, *J* = 8.3, 2.1 Hz, 1H), 7.41 (d, *J* = 2.0 Hz, 1H); ^13^C NMR (100 MHz, CDCl_3_): δ (ppm) = 10.15, 11.17, 12.60, 31.30, 49.10, 49.51, 53.47, 56.03, 59.96, 60.89, 109.95, 110.86, 113.88, 119.93, 127.13, 150.07, 150.71, 159.81, 167.68, 167.80; HRMS (ESI^+^) *m*/*z* calcd for C_20_H_28_N_3_O_4_ [M + H]^+^ 374.20743; found, 374.20677; HPLC purity 98.44% at 254 nm (*t*_R_ = 2.733 min).

#### (4-((3,5-Dimethylisoxazol-4-yl)­methoxy)-3-methoxyphenyl)­(4-(2-hydroxyethyl)­piperazin-1-yl)­methanone (**30**)

The compound was synthesized from 4-((3,5-dimethylisoxazol-4-yl)­methoxy)-3-methoxybenzoic acid (1.08 mmol, 0.300 g), HATU (1.62 mmol, 0.616 g), 2-(piperazin-1-yl)­ethan-1-ol (1.08 mmol, 0.133 mL), and DIPEA (4.32 mmol, 0.753 mL) according to general procedure C. The compound was purified by flash column chromatography with DCM/MeOH = 20/1 (v/v) as eluent. Yield 24%; orange oil; *R*_*f*_ = 0.14 (DCM/MeOH, 20:1, v/v); ^1^H NMR (400 MHz, CDCl_3_): δ (ppm) = 2.31 (s, 3H), 2.39 (s, 3H), 2.55 (br s, 4H), 2.59–2.63 (m, 2H), 3.58–3.74 (m, 6H), 3.87 (s, 3H), 4.86 (s, 2H), 6.91 (d, *J* = 8.2 Hz, 1H), 6.95 (dd, *J* = 8.2, 1.8 Hz, 1H), 7.01 (d, *J* = 1.7 Hz, 1H), 1H from OH is exchanged; ^13^C NMR (100 MHz, CDCl_3_): δ (ppm) = 10.15, 11.17, 50.82, 53.02, 55.97, 57.79, 59.35, 61.00, 110.11, 111.47, 114.26, 119.83, 129.39, 148.84, 150.21, 159.83, 167.72, 170.03; HRMS (ESI^+^) *m*/*z* calcd for C_20_H_28_N_3_O_5_ [M + H]^+^ 390.20235; found, 390.20140; HPLC purity 100.00% at 254 nm (*t*_R_ = 2.427 min).

#### 4-((3,5-Dimethylisoxazol-4-yl)­methoxy)-3-methoxy-*N*-(1-propylpiperidin-4-yl)­benzamide (**31**)

The compound was synthesized from 4-((3,5-dimethylisoxazol-4-yl)­methoxy)-3-methoxybenzoic acid (0.54 mmol, 0.150 g), HATU (0.81 mmol, 0.308 g), 1-propylpiperidin-4-amine (0.54 mmol, 0.086 mL), and DIPEA (2.16 mmol, 0.376 mL) according to general procedure C. The compound was purified by flash column chromatography with DCM/MeOH = 9/1 (v/v) as eluent. Yield 49%; white solid; *R*_*f*_ = 0.19 (DCM/MeOH, 9:1, v/v); ^1^H NMR (400 MHz, CDCl_3_): δ (ppm) = 0.94 (t, *J* = 7.4 Hz, 3H), 1.57–1.69 (m, 4H), 2.07–2.14 (m, 2H), 2.30 (s, 3H), 2.31–2.39 (m, 2H), 2.39 (s, 3H), 2.44–2.53 (m, 2H), 3.08 (bd, *J* = 12.0 Hz, 2H), 3.91 (s, 3H), 4.00–4.10 (m, 1H), 4.88 (s, 2H), 5.99 (d, *J* = 8.0 Hz, 1H), 6.91 (d, *J* = 8.3 Hz, 1H), 7.22 (dd, *J* = 8.3, 2.0 Hz, 1H), 7.41 (d, *J* = 2.0 Hz, 1H); ^13^C NMR (100 MHz, CDCl_3_): δ (ppm) = 10.15, 11.18, 11.76, 19.74, 31.60, 46.59, 52.59, 56.05, 60.51, 60.98, 110.00, 111.27, 113.98, 118.99, 128.56, 150.20, 150.28, 159.80, 166.40, 167.76; HRMS (ESI^+^) *m*/*z* calcd for C_22_H_32_N_3_O_4_ [M + H]^+^ 402.23873; found, 402.23753; HPLC purity 100.00% at 254 nm (*t*_R_ = 2.857 min).

#### 4-((3,5-Dimethylisoxazol-4-yl)­methoxy)-3-methoxy-*N*-((1-methylpyrrolidin-3-yl)­methyl)­benzamide (**32**)

The compound was synthesized from 4-((3,5-dimethylisoxazol-4-yl)­methoxy)-3-methoxybenzoic acid (0.54 mmol, 0.150 g), HATU (0.81 mmol, 0.308 g), (1-methylpyrrolidin-3-yl)­methanamine (0.54 mmol, 0.062 g), and DIPEA (2.16 mmol, 0.376 mL) according to general procedure C. The compound was purified by flash column chromatography with DCM/MeOH = 9/1 (v/v) as eluent. Yield 15%; white solid; *R*_*f*_ = 0.04 (DCM/MeOH, 9:1, v/v); ^1^H NMR (400 MHz, CDCl_3_): δ (ppm) = 1.61–1.69 (m, 1H), 2.06–2.17 (m, 1H), 2.30 (s, 3H), 2.38 (s, 3H), 2.39 (s, 3H), 2.45–2.52 (m, 1H), 2.53–2.59 (m, 1H), 2.70 (dd, *J* = 9.2, 2.4 Hz, 1H), 2.90 (td, *J* = 8.8, 3.7 Hz, 1H), 3.38–3.44 (m, 1H), 3.37–3.51 (m, 2H), 3.90 (s, 3H), 4.88 (s, 2H), 6.92 (d, *J* = 8.3 Hz, 1H), 7.29 (dd, *J* = 8.3, 2.0 Hz, 1H), 7.46 (d, *J* = 1.9 Hz, 1H), 7.61 (br s, 1H); ^13^C NMR (100 MHz, CDCl_3_): δ (ppm) = 10.24, 11.26, 28.84, 36.38, 42.05, 45.75, 56.04, 56.31, 61.03, 61.12, 110.20, 111.24, 114.36, 119.29, 128.99, 150.13, 150.21, 159.91, 167.25, 167.86; HRMS (ESI^+^) *m*/*z* calcd for C_20_H_28_N_3_O_4_ [M + H]^+^ 374.20743; found, 374.20630; HPLC purity 100.00% at 254 nm (*t*_R_ = 2.657 min).

#### 4-((3,5-Dimethylisoxazol-4-yl)­methoxy)-3-methoxy-*N*-(1-methylpiperidin-4-yl)­benzamide (**33**)

The compound was synthesized from 4-((3,5-dimethylisoxazol-4-yl)­methoxy)-3-methoxybenzoic acid (0.54 mmol, 0.150 g), HATU (0.81 mmol, 0.308 g), 1-methylpiperridin-4-amine (0.54 mmol, 0.068 mL), and DIPEA (2.16 mmol, 0.376 mL) according to general procedure C. The compound was purified by flash column chromatography with DCM/MeOH = 9/1 (v/v) as eluent. Yield 48%; white solid; *R*_*f*_ = 0.09 (DCM/MeOH, 9:1, v/v); ^1^H NMR (400 MHz, CDCl_3_): δ (ppm) = 1.54–1.65 (m, 2H), 2.01–2.09 (m, 2H), 2.14–2.23 (m, 2H), 2.30 (s, 3H), 2.32 (s, 3H), 2.38 (s, 3H), 2.85 (bd, *J* = 11.6 Hz, 2H), 3.91 (s, 3H), 3.94–4.04 (m, 1H), 4.88 (s, 2H), 5.92 (d, *J* = 7.8 Hz, 1H), 6.90 (d, *J* = 8.3 Hz, 1H), 7.21 (dd, *J* = 8.3, 2.1 Hz, 1H), 7.43 (d, *J* = 2.0 Hz, 1H); ^13^C NMR (100 MHz, CDCl_3_): δ (ppm) = 10.28, 11.30, 32.48, 46.32, 46.76, 54.69, 56.18, 61.16, 110.15, 111.50, 114.21, 118.97, 129.00, 150.32, 150.38, 159.91, 166.45, 167.87; HRMS (ESI^+^) *m*/*z* calcd for C_20_H_28_N_3_O_4_ [M + H]^+^ 374.20743; found, 374.20634; HPLC purity 100.00% at 254 nm (*t*_R_ = 2.643 min).

#### 4-((3,5-Dimethylisoxazol-4-yl)­methoxy)-*N*-(1-methylpiperidin-4-yl)­benzamide (**34**)

The compound was synthesized from 4-((3,5-dimethylisoxazol-4-yl)­methoxy)­benzoic acid (0.61 mmol, 0.150 g), HATU (0.92 mmol, 0.348 g), 1-methylpiperidin-4-amine (0.61 mmol, 0.077 mL), and DIPEA (2.44 mmol, 0.425 mL) according to general procedure C. The compound was purified by flash column chromatography with DCM/MeOH = 9/1 (v/v) as eluent. Yield 13%; white solid; *R*_*f*_ = 0.10 (DCM/MeOH, 9:1, v/v); ^1^H NMR (400 MHz, CDCl_3_): δ (ppm) = 1.51–1.61 (m, 2H), 2.00–2.07 (m, 2H), 2.12–2.18 (m, 2H), 2.28 (s, 3H), 2.29 (s, 3H), 2.41 (s, 3H), 2.79–2.84 (m, 2H), 3.93–4.02 (m, 1H), 4.82 (s, 2H), 5.90 (d, *J* = 7.7 Hz, 1H), 6.94–6.97 (m, 2H), 7.72–7.76 (m, 2H); ^13^C NMR (100 MHz, CDCl_3_): δ (ppm) = 10.27, 11.31, 32.57, 46.35, 46.71, 54.68, 59.77, 110.00, 114.63, 127.92, 128.89, 159.77, 160.96, 166.32, 167.75; HRMS (ESI^+^) *m*/*z* calcd for C_19_H_26_N_3_O_3_ [M + H]^+^ 344.19687; found,344.19585; HPLC purity 100.00% at 254 nm (*t*_R_ = 2.680 min).

#### 4-((3,5-Dimethylisoxazol-4-yl)­methoxy)-*N*-(1-isopropylpiperidin-4-yl)­benzamide (**35**)

The compound was synthesized from 4-((3,5-dimethylisoxazol-4-yl)­methoxy)­benzoic acid (0.61 mmol, 0.150 g), HATU (0.92 mmol, 0.348 g), 1-isopropylpiperidin-4-amine (0.61 mmol, 0.086 g), and DIPEA (2.44 mmol, 0.425 mL) according to general procedure C. The compound was purified by flash column chromatography with DCM/MeOH = 9/1 (v/v) as eluent. Yield 39%; white solid; *R*_*f*_ = 0.11 (DCM/MeOH, 9:1, v/v); ^1^H NMR (400 MHz, CDCl_3_): δ (ppm) = 1.06 (d, *J* = 6.6 Hz, 6H), 1.52–1.62 (m, 2H), 2.02–2.08 (m, 2H), 2.27 (s, 3H), 2.31–2.37 (m, 2H), 2.40 (s, 3H), 2.73–2.83 (m, 1H), 2.86–2.92 (m, 2H), 3.92–4.02 (m, 1H), 4.81 (s, 2H), 5.90 (d, *J* = 8.0 Hz, 1H), 6.93–6.96 (m, 2H), 7.71–7.74 (m, 2H); ^13^C NMR (100 MHz, CDCl_3_): δ (ppm) = 10.25, 11.29, 18.45, 32.61, 47.31, 47.73, 54.85, 59.74, 109.99, 114.60, 127.90, 128.89, 159.75, 160.92, 166.28, 167.73; HRMS (ESI^+^) *m*/*z* calcd for C_21_H_30_N_3_O_3_ [M + H]^+^ 372.22817; found,372.22702; HPLC purity 99.67% at 254 nm (*t*_R_ = 2.843 min).

#### (4-((3,5-Dimethylisoxazol-4-yl)­methoxy)­phenyl)­(4-(2-hydroxyethyl)­piperazin-1-yl)­methanone (**36**)

The compound was synthesized from 4-((3,5-dimethylisoxazol-4-yl)­methoxy)­benzoic acid (0.61 mmol, 0.150 g), HATU (0.92 mmol, 0.348 g), 2-(piperazin-1-yl)­ethan-1-ol (0.61 mmol, 0.075 mL), and DIPEA (2.44 mmol, 0.425 mL) according to general procedure C. The compound was purified by flash column chromatography with DCM/MeOH = 20/1 (v/v) as eluent. Yield 22%; yellow solid; *R*_*f*_ = 0.08 (DCM/MeOH, 20:1, v/v); ^1^H NMR (400 MHz, CDCl_3_): δ (ppm) = 2.29 (s, 3H), 2.42 (s, 3H), 2.55 (br s, 4H), 2.59–2.62 (m, 2H), 3.49–3.83 (m, 6H), 4.82 (s, 2H), 6.94–6.97 (m, 2H), 7.39–7.43 (m, 2H); ^13^C NMR (100 MHz, CDCl_3_): δ (ppm) = 10.31, 11.43, 53.09, 56.00, 57.89, 59.46, 59.72, 110.08, 114.67, 128.67, 129.40, 159.67, 159.83, 167.74, 170.19; HRMS (ESI^+^) *m*/*z* calcd for C_19_H_26_N_3_O_4_ [M + H]^+^ 360.19178; found,360.19092; HPLC purity 97.84% at 254 nm (*t*_R_ = 2.453 min).

#### 4-((3,5-Dimethylisoxazol-4-yl)­methoxy)-*N*-((1-methylpyrrolidin-3-yl)­methyl)­benzamide (**37**)

The compound was synthesized from 4-((3,5-dimethylisoxazol-4-yl)­methoxy)­benzoic acid (0.61 mmol, 0.150 g), HATU (0.92 mmol, 0.348 g), (1-methylpyrrolidin-3-yl)­methanamine (0.61 mmol, 0.075 mL), and DIPEA (2.44 mmol, 0.425 mL) according to general procedure C. The compound was purified by flash column chromatography with DCM/MeOH = 9/1 (v/v) as eluent. Yield 22%; yellow solid; *R*_*f*_ = 0.02 (DCM/MeOH, 9:1, v/v); ^1^H NMR (400 MHz, CDCl_3_): δ (ppm) = 1.55–1.64 (m, 1H), 2.00–2.09 (m, 1H), 2.25 (s, 3H), 2.27–2.32 (m, 1H), 2.35 (s, 3H), 2.38 (s, 3H), 2.44–2.48 (m, 1H), 2.50–2.54 (m, 1H), 2.59 (dd, *J* = 8.8, 2.7 Hz, 1H), 2.82 (td, *J* = 8.7, 3.9 Hz, 1H), 3.33–3.46 (m, 2H), 4.80 (s, 2H), 6.91–6.95 (m, 2H), 7.58 (t, *J* = 4.8 Hz, 1H), 7.73–7.77 (m, 2H); ^13^C NMR (100 MHz, CDCl_3_): δ (ppm) = 10.14, 11.17, 28.66, 36.57, 41.85, 45.07, 50.49, 56.01, 59.56, 60.54, 109.94, 114.42, 127.67, 128.85, 159.70, 160.71, 167.21, 167.64; HRMS (ESI^+^) *m*/*z* calcd for C_19_H_26_N_3_O_3_ [M + H]^+^ 344.19687; found,344.19525; HPLC purity 99.02% at 254 nm (*t*_R_ = 2.683 min).

#### 4-((3,5-Dimethylisoxazol-4-yl)­methoxy)-*N*-(1-propylpiperidin-4-yl)­benzamide (**38**)

The compound was synthesized from 4-((3,5-dimethylisoxazol-4-yl)­methoxy)­benzoic acid (0.61 mmol, 0.150 g), HATU (0.92 mmol, 0.348 g), 1-propylpiperidin-4-amine (0.61 mmol, 0.096 mL), and DIPEA (2.44 mmol, 0.425 mL) according to general procedure C. The compound was purified by flash column chromatography with DCM/MeOH = 9/1 (v/v) as eluent. Yield 40%; white solid; *R*_*f*_ = 0.23 (DCM/MeOH, 9:1, v/v); ^1^H NMR (400 MHz, CDCl_3_): δ (ppm) = 0.93 (t, *J* = 7.4 Hz, 3H), 1.54–1.72 (m, 4H), 2.08–2.13 (m, 2H), 2.27–2.33 (m, 2H), 2.29 (s, 3H), 2.42 (s, 3H), 2.44–2.47 (m, 2H), 3.01–3.08 (m, 2H), 4.01–4.09 (m, 1H), 4.83 (s, 2H), 5.98 (d, *J* = 7.9 Hz, 1H), 6.95–6.98 (m, 2H), 7.73–7.76 (m, 2H); ^13^C NMR (100 MHz, CDCl_3_): δ (ppm) = 10.17, 11.20, 11.81, 19.86, 31.80, 46.63, 52.53, 59.63, 60.54, 109.85, 114.51, 127.56, 128.80, 159.67, 160.89, 166.27, 167.66; HRMS (ESI^+^) *m*/*z* calcd for C_21_H_30_N_3_O_3_ [M + H]^+^ 372.22817; found,372.22724; HPLC purity 97.87% at 254 nm (*t*_R_ = 2.900 min).

#### 4-((3,5-Dimethylisoxazol-4-yl)­methoxy)-*N*-(1-ethylpiperidin-4-yl)­benzamide (**39**)

The compound was synthesized from 4-((3,5-dimethylisoxazol-4-yl)­methoxy)­benzoic acid (0.81 mmol, 0.200 g), HATU (1.22 mmol, 0.462 g), 1-ethylpiperidin-4-amine (0.81 mmol, 0.104 g), and DIPEA (3.24 mmol, 0.564 mL) according to general procedure D. The compound was purified by flash column chromatography using DCM/MeOH = 9/1 (v/v) as eluent. Yield 31%; white solid; *R*_*f*_ = 0.18 (DCM/MeOH, 9:1, v/v); ^1^H NMR (400 MHz, CDCl_3_): δ (ppm) = 1.10 (t, *J* = 7.2 Hz, 3H), 1.51–1.61 (m, 2H), 2.05–2.17 (m, 4H), 2.29 (s, 3H), 2.42 (s, 3H), 2.43 (q, *J* = 7.2 Hz, 2H), 2.92 (bd, *J* = 11.4 Hz, 2H), 3.92–4.08 (m, 1H), 4.83 (s, 2H), 5.86 (d, *J* = 7.6 Hz, 1H), 6.95–6.98 (m, 2H), 7.72–7.76 (m, 2H); ^13^C NMR (100 MHz, CDCl_3_): δ (ppm) = 10.17, 11.21, 12.27, 32.44, 47.12, 52.06, 52.35, 59.62, 109.85, 114.48, 127.79, 128.75, 159.67, 160.81, 166.15, 167.64; HRMS (ESI^+^) *m*/*z* calcd for C_20_H_28_N_3_O_3_ [M + H]^+^ 358.21252; found,358.21224; HPLC purity 100.00% at 254 nm (*t*_R_ = 2.737 min).

#### *tert*-Butyl 4-(4-((3,5-dimethylisoxazol-4-yl)­methoxy)­benzamido)­piperidine-1-carboxylate (**42**)

The compound was synthesized from 4-((3,5-dimethylisoxazol-4-yl)­methoxy)­benzoic acid (1.21 mmol, 0.300 g), HATU (1.82 mmol, 0.690 g), *tert*-butyl 4-aminopiperidine-1-carboxylate (1.21 mmol, 0.243 g), and DIPEA (4.84 mmol, 0.843 mL) via general procedure D. The compound was purified by flash column chromatography using EtOAc/*n*-Hex = 2/1 (v/v) as eluent. Yield 72%; colorless oil; *R*_*f*_ = 0.23 (EtOAc/*n*-hex, 2:1, v/v); ^1^H NMR (400 MHz, CDCl_3_): δ (ppm) = 1.22–1.42 (m, 2H), 1.47 (s, 9H), 1.98–2.07 (m, 2H), 2.29 (s, 3H), 2.42 (s, 3H), 2.92 (s, 2H), 4.00–4.24 (m, 3H), 4.83 (s, 2H), 5.87 (d, *J* = 7.9 Hz, 1H), 6.94–6.97 (m, 2H), 7.72–7.76 (m, 2H).

#### 4-((3,5-Dimethylisoxazol-4-yl)­methoxy)-*N*-(piperidin-4-yl)­benzamide (**43**)

*tert*-Butyl 4-(4-((3,5-dimethylisoxazol-4-yl)­methoxy)­benzamido)­piperidine-1-carboxylate (**42**) (0.39 mmol, 0.168 g) was dissolved in DCM (10 mL) and trifluoroacetic acid (3.90 mmol, 0.300 mL) and stirred at room temperature for 24 h. Solvents were evaporated, and the compound was purified by flash column chromatography using DCM/isopropanol = 7/3 (v/v)+ 1% NH_3_ as an eluent. Yield 21%; colorless oil; *R*_*f*_ = 0.24 ((DCM/isopropanol, 7:3, v/v) + 1% NH_3_); ^1^H NMR (400 MHz, acetone-*d*_6_): δ (ppm) = 1.92–2.02 (m, 2H), 2.14–2.18 (m, 2H), 2.24 (s, 3H), 2.42 (s, 3H), 3.20 (td, *J* = 12.7, 3.0 Hz, 2H), 3.55 (dt, *J* = 12.8, 3.3 Hz, 2H), 4.18–4.28 (m, 1H), 5.00 (s, 2H), 5.23 (br s, 1H), 7.02–7.06 (m, 2H), 7.90–7.95 (m, 3H); ^13^C NMR (100 MHz, acetone-*d*_6_): δ (ppm) = 10.05, 10.93, 29.33, 43.73, 45.74, 60.28, 111.09, 115.16, 128.05, 130.16, 160.32, 161.97, 166.66, 168.31; HRMS (ESI^+^) *m*/*z* calcd for C_18_H_24_N_3_O_3_ [M + H]^+^ 330.18122; found,330.18053; HPLC purity 100.00% at 254 nm (*t*_R_ = 2.643 min).

### General Procedure D: Alkylation of Amine

4-((3,5-Dimethylisoxazol-4-yl)­methoxy)-*N*-(piperidin-4-yl)­benzamide (**43**) (1 equiv) was dissolved in anhydrous acetonitrile, and an appropriate alkyl bromide (2 equiv), K_2_CO_3_ (1.5 equiv), and KI (catalytic amount) were added. The reaction mixture was stirred at reflux for 2 h. The solvent was removed under reduced pressure, and the residue was dissolved in DCM and extracted with water. The organic phase was dried over anhydrous Na_2_SO_4_, filtered, and evaporated under reduced pressure. The residue was purified by flash column chromatography.

#### *N*-(1-Butylpiperidin-4-yl)-4-((3,5-dimethylisoxazol-4-yl)­methoxy)­benzamide (**40**)

The compound was synthesized from 4-((3,5-dimethylisoxazol-4-yl)­methoxy)-*N*-(piperidin-4-yl)­benzamide (**43**) (0.46 mmol, 0.150 g), K_2_CO_3_ (0.68 mmol, 0.094 g), 1-chlorobutane (0.91 mmol, 0.096 mL), and KI (cat.) according to general procedure D. The compound was purified by flash column chromatography using DCM/MeOH = 9/1 (v/v) as eluent. Yield 8%; white solid; *R*_*f*_ = 0.37 (DCM/MeOH, 9:1, v/v); ^1^H NMR (400 MHz, CDCl_3_): δ (ppm) = 0.94 (t, *J* = 7.3 Hz, 3H), 1.30–1.39 (m, 2H), 1.75 (br s, 4H), 2.05–2.12 (m, 2H), 2.28 (br s, 2H), 2.29 (s, 3H), 2.42 (s, 3H), 2.48 (s, 2H), 3.05 (br s, 2H), 4.02–4.10 (m, 1H), 4.83 (s, 2H), 5.95 (d, *J* = 7.6 Hz, 1H), 6.95–6.99 (m, 2H), 7.72–7.76 (m, 2H); ^13^C NMR (100 MHz, CDCl_3_): δ (ppm) = 10.16, 11.20, 13.94, 20.69, 28.51, 31.59, 46.54, 52.41, 58.25, 59.63, 109.86, 114.49, 127.56, 128.83, 159.66, 160.88, 166.21, 167.64; HRMS (ESI^+^) *m*/*z* calcd for C_22_H_32_N_3_O_3_ [M + H]^+^ 386.24382; found,386.24272; HPLC purity 100.00% at 254 nm (*t*_R_ = 3.110 min).

#### *N*-(1-Allylpiperidin-4-yl)-4-((3,5-dimethylisoxazol-4-yl)­methoxy)­benzamide (**41**)

The compound was synthesized from 4-((3,5-dimethylisoxazol-4-yl)­methoxy)-*N*-(piperidin-4-yl)­benzamide (**43**) (0.91 mmol, 0.300 g), K_2_CO_3_ (1.37 mmol, 0.189 g), 3-bromoprop-1-ene (1.82 mmol, 0.158 mL), and KI (cat.) according to general procedure D. The compound was purified by flash column chromatography using DCM/MeOH = 9/1 (v/v) as eluent. Yield 26%; white solid; *R*_*f*_ = 0.34 (DCM/MeOH, 9:1, v/v); ^1^H NMR (400 MHz, CDCl_3_): δ (ppm) = 1.60–1.70 (m, 2H), 2.03–2.11 (m, 2H), 2.17–2.26 (m, 2H), 2.29 (s, 3H), 2.42 (s, 3H), 2.96–2.99 (m, 2H), 3.06–3.09 (m, 2H), 3.98–4.08 (m, 1H), 4.83 (s, 2H), 5.19–5.25 (m, 2H), 0.86–5.96 (m, 2H), 6.95–6.98 (m, 2H), 7.72–7.76 (m, 2H); ^13^C NMR (100 MHz, CDCl_3_): δ (ppm) = 10.17, 11.20, 31.78, 46.62, 52.18, 59.61, 61.44, 109.86, 114.47, 119.43, 127.55, 128.84, 133.59, 159.66, 160.85, 166.24, 167.64; HRMS (ESI^+^) *m*/*z* calcd for C_21_H_28_N_3_O_3_[M + H]^+^ 370.21252; found,370.21145. HPLC purity, 98.08% at 254 nm (*t*_R_ = 2.867 min).

### Cell Culture

Human embryonic Kidney (HEK)-Blue Null1, HEK-Blue hTLR2-TLR1, HEK-Blue hTLR2-TLR6, HEK-Blue hTLR4, HEK-Blue hTLR5, HEK-Blue hTLR7, HEK-Blue hTLR8, and HEK-Blue hTLR9 (InvivoGen, Toulouse, France) were cultured in Dulbecco’s modified Eagle’s medium (PAN-Biotech, Aidenbach, Germany) containing 10% (v/v) heat inactivated fetal bovine serum (FBS; S0615), 100 U/mL penicillin, 100 mg/mL streptomycin (P4333), 2 mM 1-glutamine (G7513) (all from Sigma-Aldrich, Taufkirchen, Germany), 100 μg/mL Normocin and selective antibiotics HEK-Blue Selection (hTLR2-TLR1, hTLR2-TLR6, hTLR4), 100 μg/mL zeocin (Null1), 100 μg/mL zeocin with 10 μg/mL (hTLR7, hTLR9), and 30 μg/mL (hTLR5, hTLR8) blasticidin (all from InvivoGen, Toulouse, France). THP1-Dual TLR4/MD-2/CD14 cells (InvivoGen, Toulouse, France) were cultured in RPMI-1640 medium supplemented with 10% fetal bovine serum (FBS), penicillin (100U/mL), streptomycin (100 μg/mL), l-glutamine (2 mM), HEPES (25 mM), Normocin (100 μg/mL), and selective antibiotics (blasticidin: 10 μg/mL, zeocin: 100 μg/mL) following the manufacturer’s instructions.

THP-1 cells (ACC 16, DSMZ-German Collection of Microorganisms and Cell Cultures GmbH, Braunschweig, Germany) were cultured in RPMI 1640 (11530586, Fisher scientific, Schwerte, Germany) containing 100 U/mL penicillin, 100 μg/mL streptomycin (P4333), 2 mM l-glutamine (G7513, both from Sigma-Aldrich, Taufkirchen, Germany), and 10% heat-inactivated fetal bovine serum (FBS; S0615, Sigma- Aldrich, Taufkirchen, Germany) at a density of 4 × 10^5^ cells/mL to 2 × 10^6^ cells/mL. For generating THP-1-derived macrophages, THP-1 monocytes were seeded into 24-well plates at a density of 4 × 10^5^ cells/mL in growth medium including 25 ng/mL PMA (phorbol 12-myristate 13-acetate; tlrl-pma, Invivogen, Toulouse, France). After 48 h, adherent cells were carefully washed with PBS (phosphate buffered saline; P04-53500, Pan Biotechne, Aidenbach, Germany) and rested in PMA-free medium for 24 h. All cell lines were maintained at 37 °C in a humidified atmosphere of 5% CO2 and 95% air and were regularly tested negative for mycoplasma contamination (VenorGeM Classic Mycoplasma PCR detection kit, Minerva Biolabs, Berlin, Germany).

PBMCs (peripheral blood mononuclear cells) were obtained from buffy-coat donations (Institute of Experimental Haematology and Transfusion Medicine, University Clinic Bonn) and isolated by density gradient centrifugation using Biocoll separation media (Bio&Sell, Nuremberg, Germany). PMBCs were washed three times with PBS containing ETDA and afterward seeded in 24-well plates (5 × 10^6^ cells/well). The studies with human blood were approved by the ethics committee of the University Clinic Bonn (315/22) and written informed consent was obtained from all healthy donors.

### Plasmids

Human embryonic Kidney (HEK)-Blue Null1 Cells (InvivoGen, Toulouse, France) were seeded into 6-well plates at a density of 1 × 10^6^ cells/mL. After 24 h, adherent cells were washed with PBS and afterward transfected with plasmids using PEI Max. After another 24 h, cells were detached with TrypLE Express (Thermo Fisher Scientific, Darmstadt, Germany), seeded into 96-well plates at a density of 4 × 10^4^ cells/mL, and rested for 24 h in medium containing selective antibiotics for hTLR8 cells (as described above). Plasmids encoding mutant TLR8 (TLR8^F513L^) were prepared from the TLR8^WT^ construct (pUNO1-hTLR08a, NM_016610.4, InvivoGen, Toulouse, France) by site-directed mutagenesis (Q5 Site-Directed Mutagenesis Kit, NEB, Frankfurt am Main, Germany) using the primer pairs 5′-AAGAGGTTATATGTTCCAGGAAC-3′ and 5′-AAATGCAATGCCCGTAGAG-3′ (synthesized by Thermo Fisher Scientific, Darmstadt, Germany). Successful mutagenesis was confirmed by Sanger sequencing. TLR8 transfection and overexpression was confirmed using Western Blot (Figure S11).

### Cell Stimulation

HEK-Blue cells (4 × 10^4^ cells/well) and THP-1 macrophages (4 × 10^4^ cells/well, 4 × 10^5^ cells/well and 8 × 10^5^ cells/well) were seeded in 96-well plates, 24-well plates, or 6-well plates (PS, Sarsted, Germany), respectively. For stimulation experiments, cells were washed with phosphate-buffered saline (PBS, Sigma-Aldrich) and media was replaced with OptiMEM (Thermo Fisher Scientific, Darmstadt, Germany). For inhibition studies, the cells were preincubated with TLR8 antagonists for 1 h and afterward stimulated with TLR agonists for 24 h. The following TLR ligands were used: Pam_2_CSK_4_, Pam_3_CSK_4_, poly­(I:C) (HMW), LPS from *Escherichia coli* O111:B4 (LPS-EB Ultrapure), flagellin from *Bacillus subtilis* (flagellin-BS Ultrapure), CL307, CL075, R848, TL8-506, ODN2006 (all from InvivoGen, Toulouse, France), and Enpatoran (BIOZOL Diagnostika, Germany). After 24 h, NF-κB activity was measured using QuantiBlue solution (InvivoGen, Toulouse, France) following the manufacturer’s instructions.

THP1-Dual TLR4/MD-2/CD14 cells (InvivoGen, Toulouse, France) were seeded in 96-well plates at a density of 1 × 10^5^ cells per well and were immediately preincubated with designated antagonists for 1 h and afterward stimulated with TLR agonists. After 24 h, NF-κB activity was measured via the SEAP reporter assay using QuantiBlue solution (InvivoGen, Toulouse, France) and ISRE activity was measured via lucia luciferase using QuantiLuc solution (InvivoGen, Toulouse, France), both following the manufacturer’s instructions.

The TLR8 antagonists synthesized and commercially purchased were dissolved in DMSO as a 50 mM stock solution. Final DMSO concentrations in the cell culture were below 0.2% (v/v). The cells were first incubated with the antagonists for 1 h and afterward stimulated with the respective TLR agonist.

### Cell Viability

Effects on cell viability were assessed by the MTT assay. HEK-Blue hTLR7, HEK-Blue hTLR8 cells, or differentiated THP-1 macrophages (40,000 cells/well, 96 well plate) were preincubated with TLR8 antagonists for 1 h and afterward stimulated for 20 h with TLR7 or TLR8 agonists. Subsequently, 25 μL of MTT (3-(4,5-dimethylthiazol-2-yl)-2,5-diphenyltetrazolium bromide, 5 mg/mL) was added and incubated for 4 h at 37 °C. After the supernatants were removed, DMSO (4720.1, Carl Roth, Karlsruhe, Germany) was added and absorption at 540 nm was measured. Viability of the untreated cells was defined as 100%. DMSO (10% (v/v); A994.1, Carl Roth, Karlsruhe, Germany) served as a positive control.

In selected experiments, LDH assay was performed according to the manufacturer’s instructions (Thermo fisher Scientific, Darmstadt, Germany). The percentage of LDH release was calculated compared to the 100% cell lysis control.

### Enzyme-Linked Immunosorbent Assay

After 4 h of stimulation of THP-1 macrophages or PBMCs with the respective TLR agonists, cell culture supernatants were collected and analyzed using commercially available human TNF or IL-1β secretion ELISA kits (88-7346-88, 88-7261-88; Thermo Fisher Scientific, Darmstadt, Germany).

### Dynamic Mass Redistribution (DMR) Label-Free Assay

DMR assays were conducted using the EPIC system (Corning) in accordance with established protocols.^42,63,64^ On the day of the assay, THP-1 Dual TLR4-MD2-CD14 cells were seeded as suspension cells at a density of 40,000 cells per well in assay buffer (Hank’s Balanced Salt Solution (HBSS) with 20 mM HEPES, pH 7.0) into an Epic 384-well uncoated glass microplate (Corning, New York, NY, USA). Cells were briefly centrifuged for 10 s to ensure proper contact with the bottom biosensor and prevent drops from adhering to the sides of the wells. Each well had a final volume of 30 μL. After cell seeding, the Epic microplates were incubated in the EPIC instrument at 37 °C for 1.5 h. Serial dilutions of compounds were prepared in the same assay buffer. DMR measurements were performed using the Epic biosensor, and following baseline readings, 10 μL of compounds were added to each well (40 μL total volume) using a semiautomated liquid handler, Selma (Analytik Jena AG, Jena, Germany). The antagonist was preincubated for 1.5 h before the addition of the agonist. DMR signals were recorded for 15,000 s, and the data were analyzed and exported using the Epic Analyzer Software (Corning, New York, NY, USA). All signals were baseline-corrected, and compound responses were represented as picometer (picosecond) shifts over time (minutes) following baseline normalization. Experiments were performed at 37 °C in triplicate or quadruplicate.

### Western Blotting

The protein amount was quantified by a bicinchoninic acid assay (Pierce BCA Protein Assay Kit; 23227, Thermo Fisher Scientific, Darmstadt, Germany). Twenty-five μg protein per lane was separated on a 10% TGX Stain-Free FastCast acrylamide gel (1610183, Bio-Rad, Feldkirchen, Germany) containing TEMED (2367, Carl Roth, Karlsruhe, Germany) and ammonium persulfate (A3678, Sigma-Aldrich, Taufkirchen, Germany) using MiniPROTEAN electrophoresis system (Bio-Rad). Gels were blotted on low fluorescence polyvinylidene difluoride membranes (Immobilon-FL PVDF low fluorescence; 05317, Merck, Darmstadt, Germany) using the Trans-Blot Turbo System (Bio-Rad, Feldkirchen, Germany). Membranes were blocked using 5% milk (T145.2, Carl Roth, Karlsruhe, Germany) in TBS-T buffer consisting of TRIS HCl (T3253, Sigma-Aldrich, Taufkirchen, Germany), NaCl (27810.295, VWR, Darmstadt, Germany), and Tween 20 (9127.1, Carl Roth, Karlsruhe, Germany). Membranes were incubated overnight at 4 °C with either mouse anti-TLR8 mAb (sc-373760, SCBT), rabbit anti-MyD88 mAb (4283, CST), rabbit anti-Phospho-NF-κB p65 (Ser536) (no. 93H1), rabbit anti-NF-κB p65 (D14E12) XP (no. 8242), or rabbit anti-IκBα antibody (no. 9242) in 5% BSA. Afterward, they were washed three times with TBS-T and incubated with either rabbit anti-IgG HRP conjugated antibody (no. 7074) or mouse anti-IgG HRP conjugated antibody (no. 7076), all from Cell Signaling Technology (CST, Leiden, The Netherlands), for one h at RT. Blots were developed with ECL reagent (Clarity Western ECL Substrate; 1705060, Bio-Rad, Feldkirchen, Germany) and imaged using ChemiDoc imaging system (Bio-Rad, Feldkirchen, Germany). Values of protein expression were analyzed by densitometry and normalized to total protein levels using Image lab 6.1 Bio-Rad, Feldkirchen, Germany. Uncropped Western blots are provided in Figure S10A.

### Co-Immunoprecipitation

THP-1 macrophages were lysed in cell lysis buffer (RIPA) with a protease/phosphatase inhibitor cocktail (NEB, Frankfurt am Main, Germany). The total amount of protein was quantified by using bicinchoninic acid assay (Pierce BCA Protein Assay Kit; 23227, Thermo Fisher Scientific, Darmstadt, Germany) and equivalent amounts of each sample were used for coimmunoprecipitation. Lysates were incubated with mouse anti-TLR8 mAb (sc-373760, SCBT, Heidelberg, Germany) for 2 h at 4 °C. Mouse anti-IgG2b mAb (no. 53484, CST, Leiden, The Netherlands) served as the isotype control. Next, protein A/G PLUS agarose beads (sc-2003, SCBT, Heidelberg, Germany) were added for overnight incubation at 4 °C followed by washing with RIPA buffer 4 times. The immunoprecipitated proteins were removed from the beads with standard SDS-PAGE sample buffer in the presence of DTT by boiling for 5 min at 95 °C. Afterward, the samples were analyzed by Western blot. The nonimmunoprecipitated cell lysate was used as loading control. Uncropped Western blots are provided in Figure S10B.

### RNA Isolation, cDNA Synthesis, and qRT-PCR

Total RNA isolation was performed using innuPREP RNA mini kit 2.0 (845-KS-2040050, AnalytikJena, Jena, Germany) according to the manufacturer’s protocol. Synthesis of cDNA was carried out using an iScript cDNA synthesis kit (1708891, Bio-Rad, Feldkirchen, Germany). Quantitative real-time RT-PCR (qRT-PCR) was performed as described before.^65^ Primers (synthesized by TIB Molbiol, Berlin, Germany or Eurofins Genomics, Ebersberg, Germany) with the following sequences were used: *GAPDH*, 5′-CTCTCTGCTCCTCCTGTTCGAC-3′ and 5′- TGAGCGATGTGGCTCGGCT-3′; *TNF*, 5′-CCCAGGGACCTCTCTAATC-3′ and 5′-GCTACAGGCTTGTCACTCGG-3′, *IL1B*, 5′-TGGAGCAACAAGTGGTGT-3′ and 5′-TTGGGATCTACACTCTCCAGC-3′. Fold difference in gene expression was normalized to the housekeeping gene *GAPDH* showing the most constant expression levels. The reaction mix containing cDNA template, primers, and SYBR green (iTaq Universal SYBR Green Supermix; 172–5125, Bio-Rad, Feldkirchen, Germany) was run under the conditions as described.

### Statistical Analysis

Data are expressed as mean ± SEM. For multiple comparisons, statistically significant differences were determined by one-way ANOVA followed by a Dunnett’s or Tukey’s post-test and considered significant at *P ≤ 0.05, **P ≤ 0.01, ***P ≤ 0.001, ****P ≤ 0.0001. All other values were calculated accordingly. Statistical differences were assessed by a one-sample *t*-test against 100%. Statistical analysis was performed using GraphPad Prism (version 8.0, GraphPad software, San Diego, USA).

## Supplementary Material









## Data Availability

All data generated or analyzed during this study are included in the article and its Supporting Information.

## Notes

Chemical structures of known cocrystallized TLR8 antagonists; validation of the virtual screening 3D pharmacophore; NF-κB activity and cell viability in hTLR8-HEK293 cells for compounds **1**-**12**; concentration–response curves in hTLR8-HEK293 cells for compounds **1**, **10**–**12**; concentration–response curves in hTLR7-HEK293 cells for compounds **10**–**12**; NF-κB activity and cell viability in hTLR8-HEK293 cells for compounds **17**–**41**, **43**; concentration–response curves in hTLR8-HEK293 cells for synthesized compounds; cell viability of compounds **10** and **12** in THP-1 macrophages and PMBCs; inhibition of TLR8-dependent activity in THP-1 Dual cells; whole uncropped images of the original Western blots; transfection of mutant and wildtype TLR8 plasmids into HEK293 cells; HPLC traces for compounds **1**–**12**; ^1^H NMR spectra for purchased active compounds **1**, **10**–**12**; ^1^H NMR, ^13^C NMR, and HPLC traces for synthesized active compounds; chemical structures of compounds **1**–**12** containing the isoxazole scaffold; inhibition of NF-κB activity in hTLR8-HEK293 cells by compounds **1**–**12**; inhibition of NF-κB activity in hTLR8-HEK293 cells by compounds **17**–**41**, **43**; chemical structures of synthesized compounds **17**–**41**, **43**; interaction frequencies of **10** with TLR8 homodimer during MD simulation; pharmacological parameters (EC_50_, *E*_max_) of compound **10**, and references (PDF)
Docking model (7rc9_1_12) (PDB)
Docking model (7rc9_17_42) (PDB)
Molecular formula strings (CSV)

## Abbreviations

CD14: cluster of differentiation 14
CLE: cutaneous lupus erythematosus
DAMP: damage-associated molecular pattern
ELISA: enzyme-linked immunosorbent assay
HBA: hydrogen bond acceptor
HEK: human embryonic kidney
HYD: hydrophobic contacts
IC50: half-maximal inhibitory concentration
IFN: interferon
IL: interleukin
IRF: Interferon regulatory factor
LDH: lactate dehydrogenase
MD: molecular dynamics
NF-κB: nuclear factor κB
PAMP: pathogen-associated molecular pattern
PBMC: peripheral blood mononuclear cell
PDB: protein data bank
PI: positive ionizable
PRR: pattern recognition receptor
RA: rheumatoid arthritis
SAR: structure–activity relationship
SEAP: secreted alkaline phosphatase
SLE: systemic lupus erythematosus
TLR: Toll-like receptor
TNF: tumor necrosis factor
