# Supplementary material for: Discovery of Novel Isoxazole-Based Small-Molecule Toll-Like Receptor 8 Antagonists
Source: J Med Chem. 2025 Feb 14;68(4):4888–907. doi: 10.1021/acs.jmedchem.4c03148 (PMC12613816; doi:10.1021/acs.jmedchem.4c03148)
Supplement: Supplementary file 1 [file jm4c03148_si_001.pdf]

Supporting Information

Discovery of novel isoxazole-based small-molecule Toll-like receptor 8 antagonists

Troy Matziol[1#], Valerij Talagayev[2#], Tjaša Slokan[3], Nika Strašek Benedik[3], Janine Holze[1], Matej Sova[3*], Gerhard Wolber[2*], Günther Weindl[1*]

[1] University of Bonn, Pharmaceutical Institute, Pharmacology and Toxicology Section, Gerhard-Domagk-Str. 3, 53121 Bonn, Germany
[2] Freie Universität Berlin, Institute of Pharmacy, Pharmaceutical and Medicinal Chemistry, Königin-Luise-Str. 2+4, 14195 Berlin, Germany
[3] University of Ljubljana, Faculty of Pharmacy, The Department of Pharmaceutical Chemistry, Aškerčeva 7, 1000 Ljubljana, Slovenia

[#]These authors contributed equally to this work

[*]Corresponding authors. e-mail: matej.sova@ffa.uni-lj.si, gerhard.wolber@fu-berlin.de, guenther.weindl@uni-bonn.de

Table of contents

Figure S1. Chemical structures of known co-crystalized TLR8 antagonists. 2
Figure S2. Validation of the virtual screening 3D pharmacophore. 3
Figure S3. NF-κB activity and cell viability in hTLR8-HEK293 cells for compounds 1-12. 4
Figure S4. Concentration-response curves in hTLR8-HEK293 cells for compounds 1, 10-12. 5
Figure S5. Concentration-response curves in hTLR7-HEK293 cells for compounds 10-12. 6
Figure S6. NF-κB activity and cell viability in hTLR8-HEK293 cells for compounds 17-41, 43. 7
Figure S7. Concentration-response curves in hTLR8-HEK293 cells for synthesized compounds. 8
Figure S8. Cell viability of compounds 10 and 12 in THP-1 macrophages and PMBCs. 9
Figure S9. Inhibition of TLR8-dependent activity in THP-1 Dual cells. 10
Figure S10. Whole uncropped images of the original western blots. 11
Figure S11. Transfection of mutant and wildtype TLR8 plasmids into HEK293 cells. 12
Figure S12. HPLC traces for purchased compounds 1-12. 13
Figure S13. ^{1}H NMR spectra for purchased active compounds 1, 10-12. 25
Figure S14. ^{1}H NMR, ^{13}C NMR and HPLC traces for synthesized active compounds. 29
Table S1. Chemical structures of compounds 1-12 containing the isoxazole scaffold. 53
Table S2. Inhibition of NF-κB activity in hTLR8-HEK293 cells by compounds 1-12. 56
Table S3. Inhibition of NF-κB activity in hTLR8-HEK293 cells by compounds 17-41, 43. 57
Table S4. Chemical structures of synthesized compounds 17-41, 43. 58
Table S5. Interaction frequencies of 10 with TLR8 homodimer during MD simulation. 60
Table S6. Pharmacological parameters (EC_{50}, E_{max}) of compound 10. 61
References 62

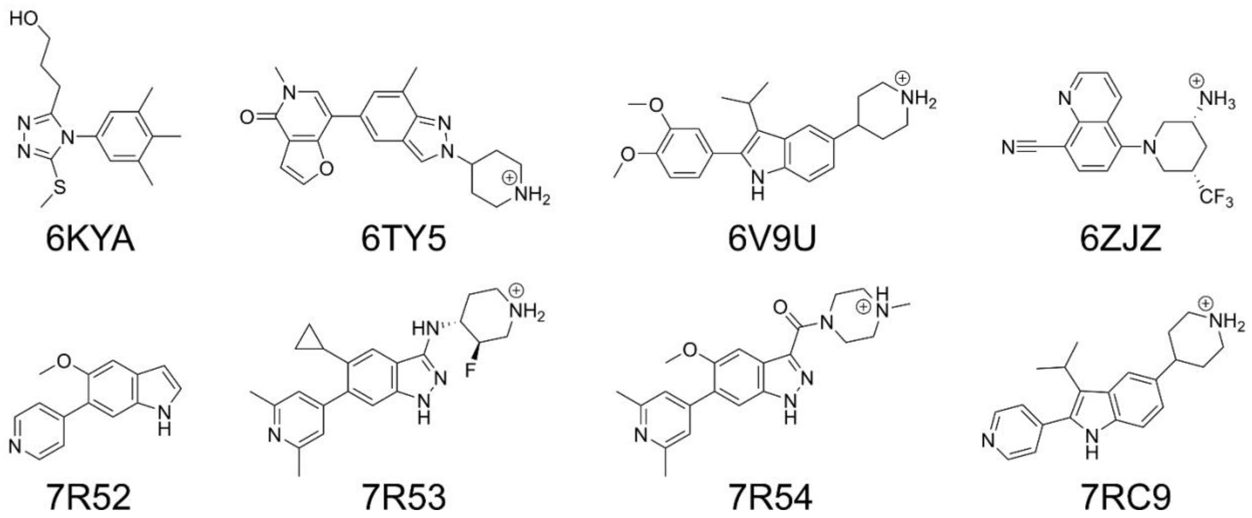

Figure S1. Chemical structures of known co-crystalized TLR8 antagonists.

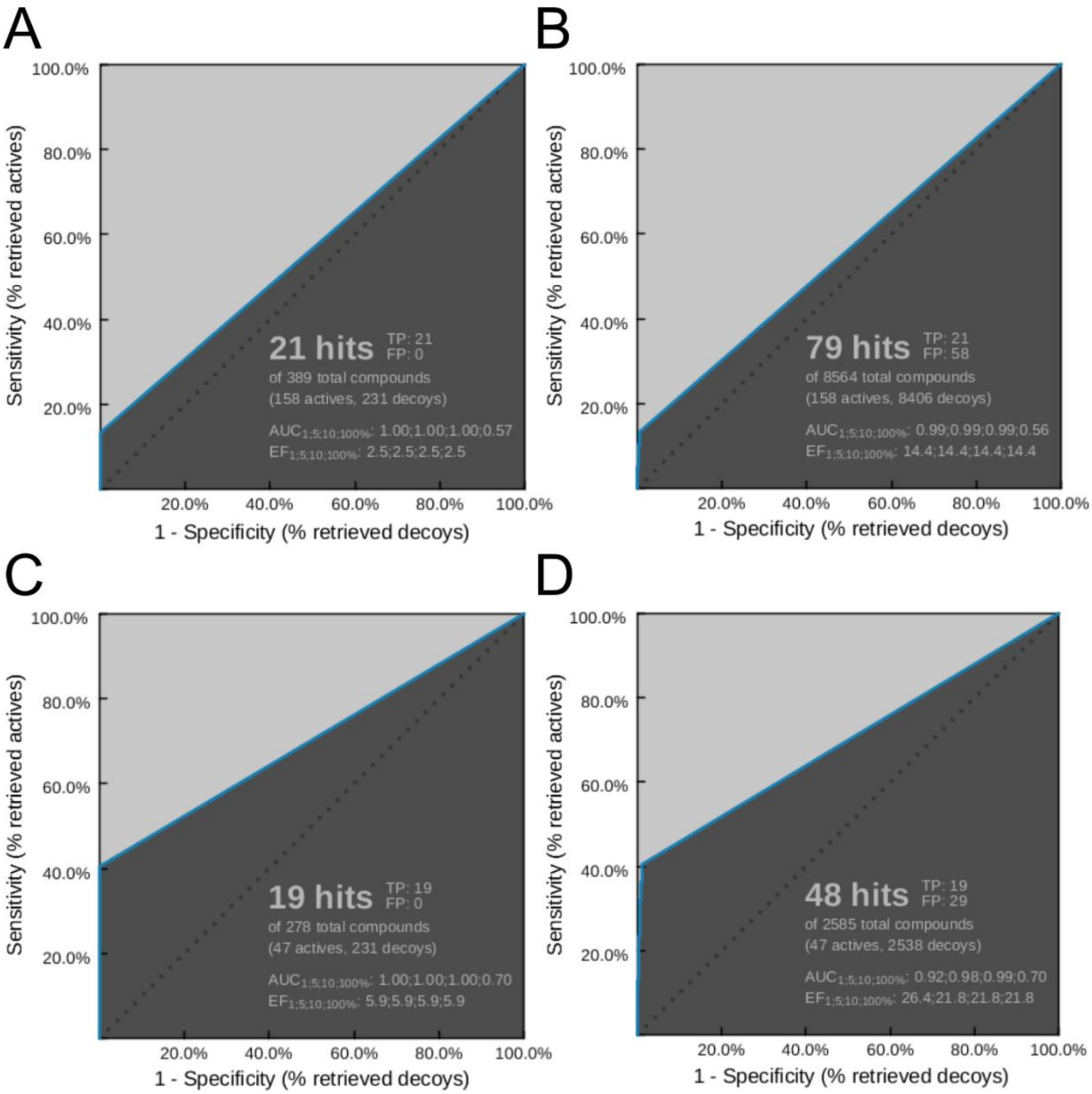

Figure S2. Validation of the virtual screening 3D pharmacophore.

(A) Validation of the 3D pharmacophore[1–3] with set A actives with an IC_{50} below 1000 nM and inactives obtained from ChEMBL.[4–6] (B) Validation of the 3D pharmacophore with set A actives and decoys obtained from DUD-E.[7] (C) Validation of the 3D pharmacophore with set B actives with an IC_{50} below 50 nM and inactives obtained from ChEMBL. (D) Validation of the 3D pharmacophore with set B actives with an IC_{50} below 50 nM and decoys obtained from DUD-E.

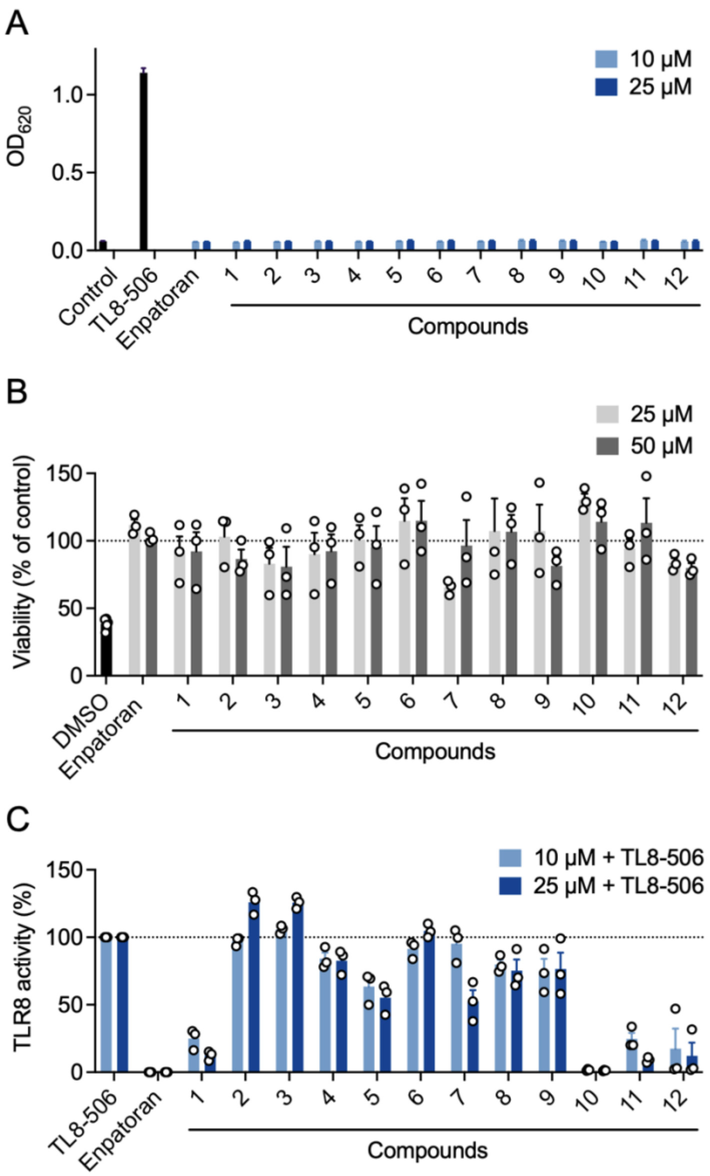

Figure S3. NF-κB activity and cell viability in hTLR8-HEK293 cells for compounds **1-12**.

(A) Activation of TLR8-dependent NF-κB activity in cells. HEK-Blue hTLR8 cells were stimulated with TL8-506 (0.6 µM) or the compounds (10 µM, 25 µM) for 24 h. Supernatants were analyzed for TLR8-mediated NF-κB activation by SEAP reporter assay using QuantiBlue (OD_{620}). Mean + SEM (n=3). (B) HEK-Blue hTLR8 cells were incubated with the compounds (25, 50 µM) for 24 h. Cell viability was analyzed using the MTT assay, and normalized to non-stimulated cells (vehicle control). DMSO (10%, v/v) was used as the cytotoxic control. Mean + SEM (n=3). (C) HEK-Blue hTLR8 cells were preincubated with the compounds (10 µM, 25 µM) for 1 h, and then stimulated with TL8-506 (0.6 µM) for 24 h. Supernatants were analyzed for TLR8-mediated NF-κB activation by SEAP reporter assay using QuantiBlue (OD_{620}), and normalized to TL8-506 alone. Mean + SEM (n=3).

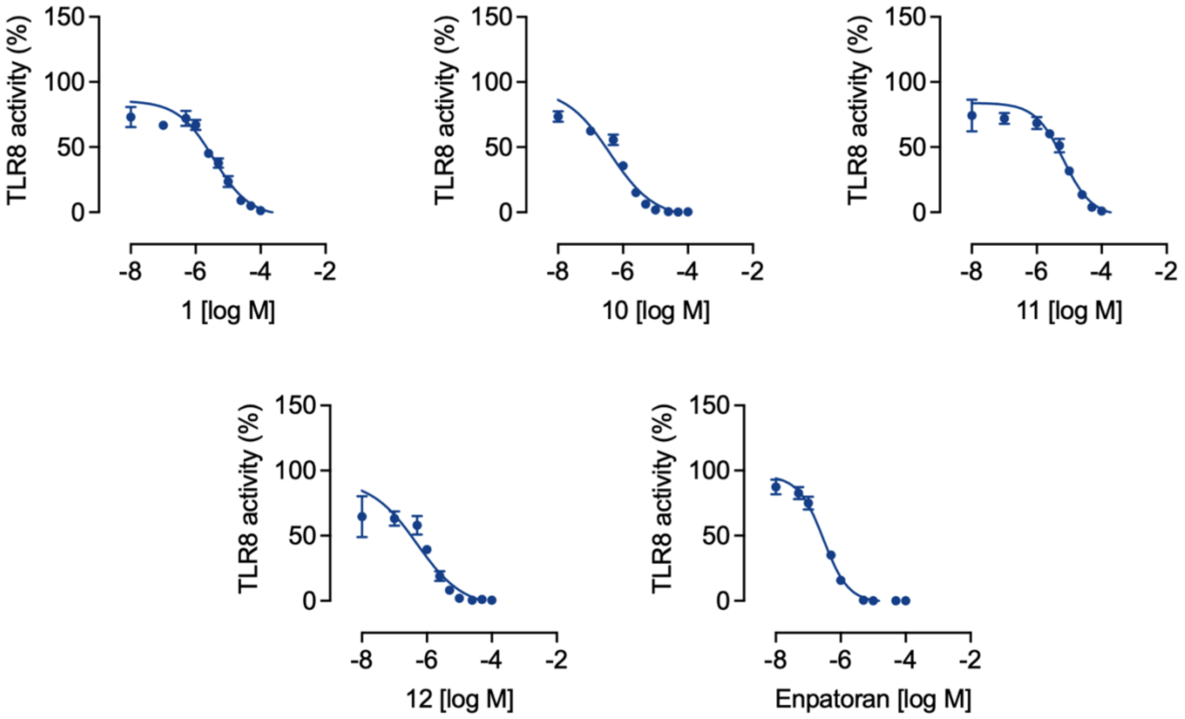

Figure S4. Concentration-response curves in hTLR8-HEK293 cells for compounds **1**, **10-12**. HEK-Blue hTLR8 cells were preincubated with increasing concentrations of the compounds for 1 h, and then stimulated with TL8-506 (0.6 μM) for 24 h. Supernatants were analyzed for TLR8-mediated NF-κB activation by SEAP reporter assay using QuantiBlue (OD_{620}). For the calculation of the concentration-response curve nonlinear regression with variable slope (four parameters) was used. IC_{50} values are shown in Table 1. Mean ± SEM (n=3-4).

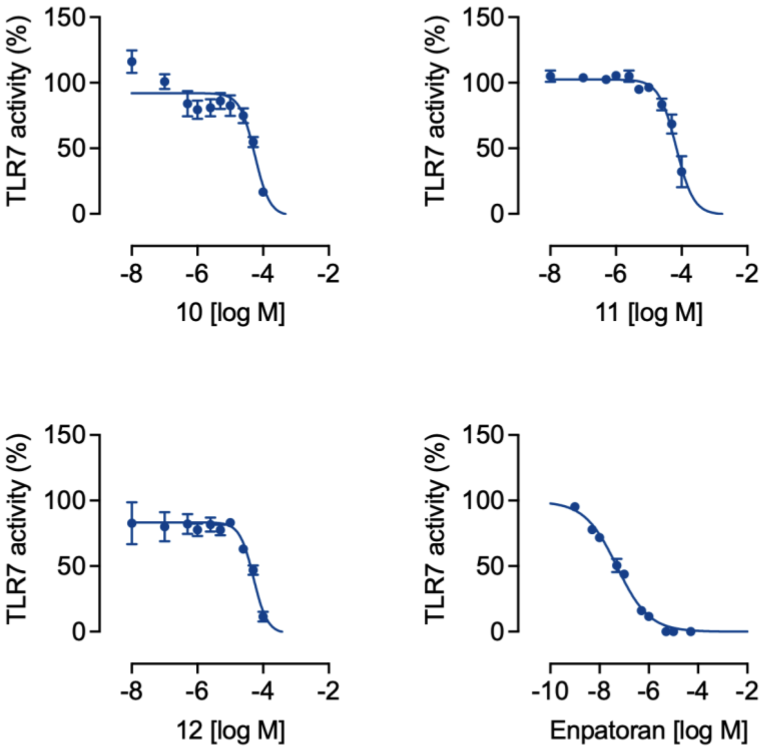

Figure S5. Concentration-response curves in hTLR7-HEK293 cells for compounds **10-12**.

HEK-Blue hTLR7 cells were preincubated with increasing concentrations of the compounds for 1 h, and then stimulated with CL307 (1.7 µM) for 24 h. Supernatants were analyzed for TLR7-mediated NF-κB activation by SEAP reporter assay using QuantiBlue (OD_{620}). For the calculation of the concentration-response curve nonlinear regression with variable slope (four parameters) was used. IC_{50} values are shown in Table 1. Mean ± SEM (n=4).

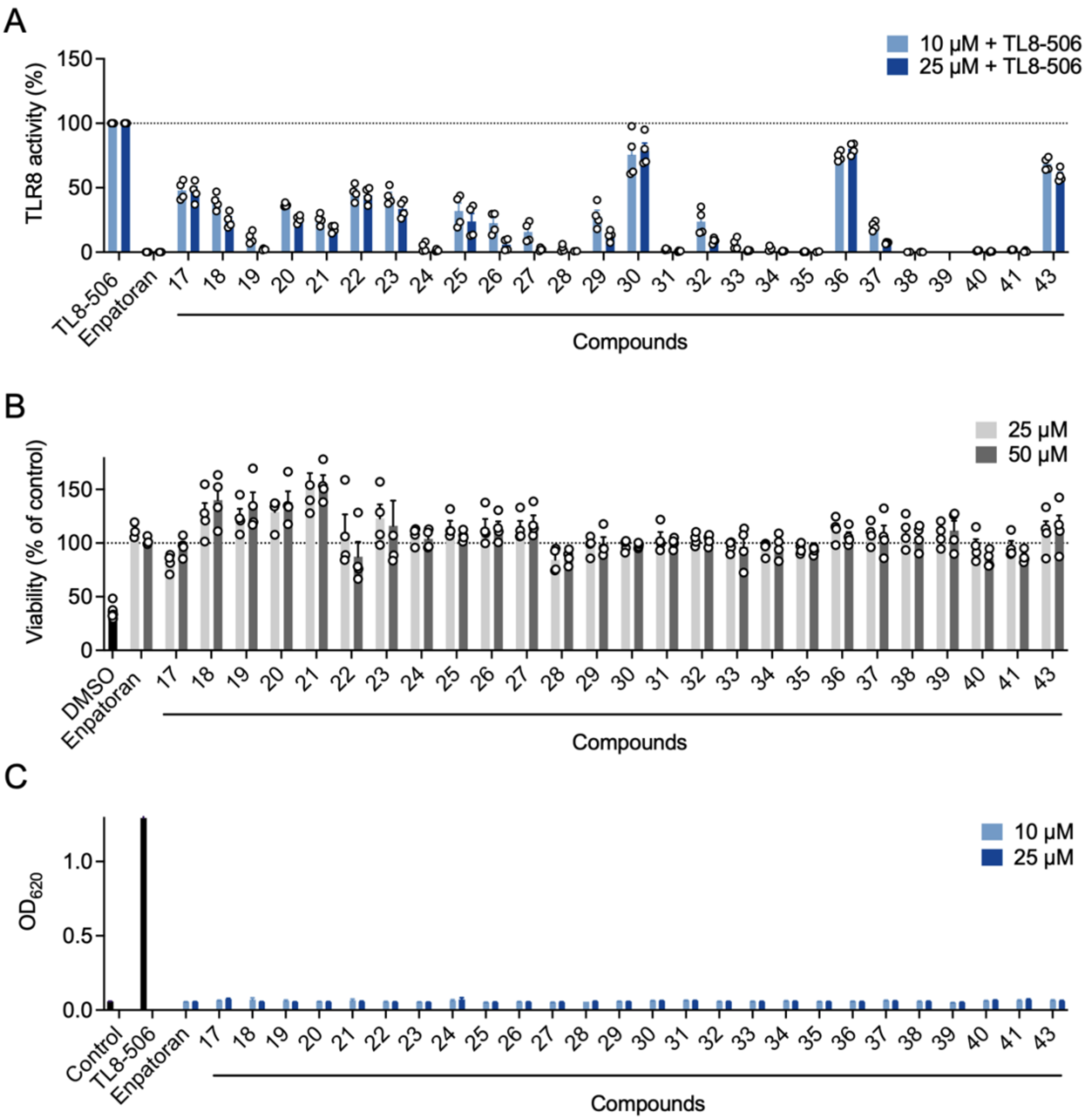

Figure S6. NF-κB activity and cell viability in hTLR8-HEK293 cells for compounds **17-41, 43**.

(A) HEK-Blue hTLR8 cells were preincubated with the compounds (10 µM, 25 µM) for 1 h, and then stimulated with TL8-506 (0.6 µM) for 24 h. Supernatants were analyzed for TLR8-mediated NF-κB activation by SEAP reporter assay using QuantiBlue (OD_{620}), and normalized to TL8-506 alone. Mean + SEM (n=4). (B) HEK-Blue hTLR8 cells were incubated with the compounds (25, 50 µM) for 24 h. Cell viability was analyzed using the MTT assay, and normalized to non-stimulated cells (vehicle control). DMSO (10%, v/v) was used as the cytotoxic control. Mean + SEM (n=4). (C) Activation of TLR8-dependent NF-κB activity in cells. HEK-Blue hTLR8 cells were stimulated with TL8-506 (0.6 µM) or the compounds (10 µM, 25 µM) for 24 h. Supernatants were analyzed for TLR8-mediated NF-κB activation by SEAP reporter assay using QuantiBlue (OD_{620}). Mean + SEM (n=4).

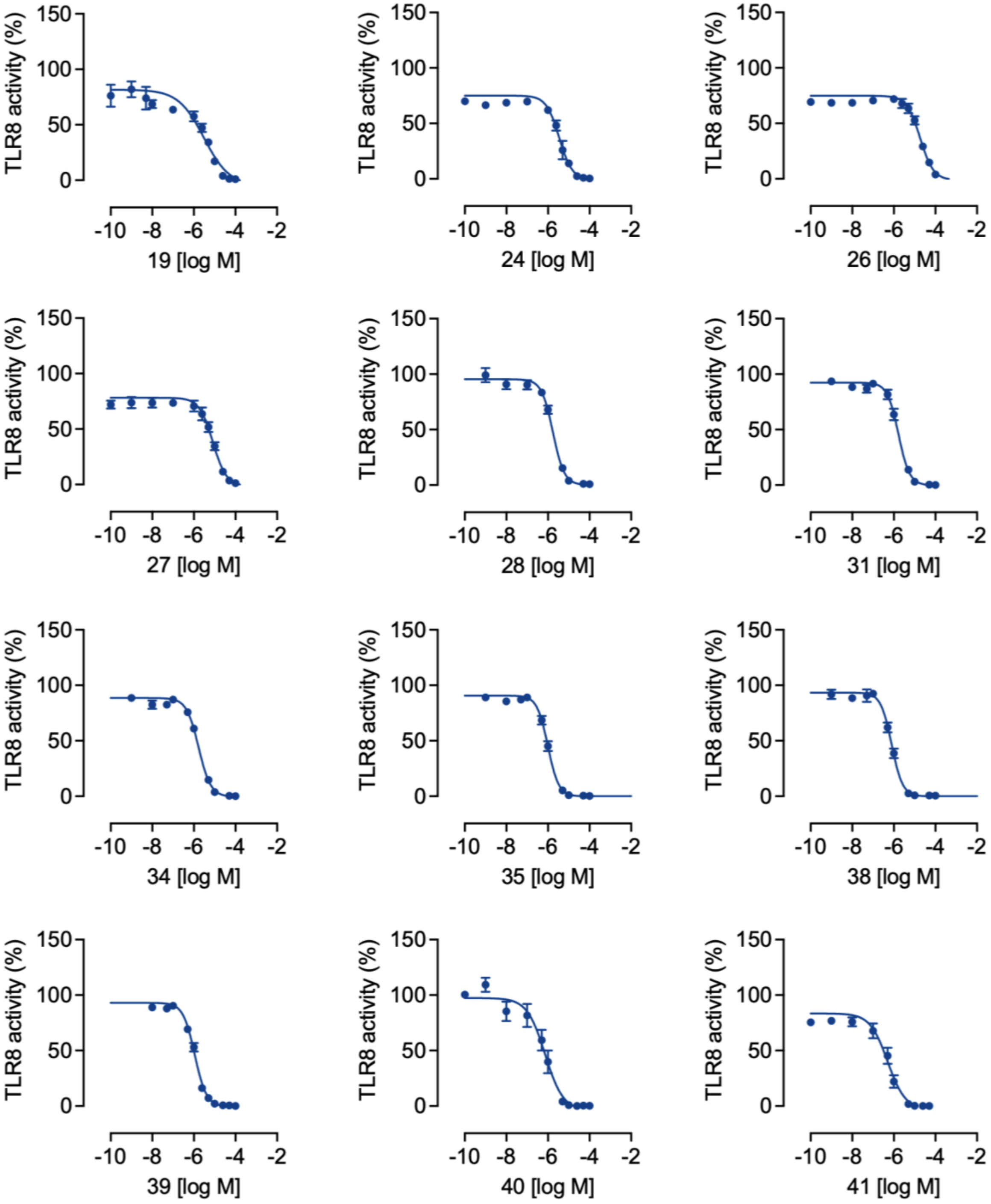

Figure S7. Concentration-response curves in hTLR8-HEK293 cells for synthesized compounds. HEK-Blue hTLR8 cells were preincubated with increasing concentrations of the compounds **19**, **24**, **26-28**, **31**, **34**, **35**, **38-41** for 1 h, and then stimulated with TL8-506 (0.6 μM) for 24 h. Supernatants were analyzed for TLR8-mediated NF-κB activation by SEAP reporter assay using QuantiBlue (OD_{620}). For the calculation of the concentration-response curves nonlinear regression with variable slope (four parameters) was used. IC_{50} values are shown in Table 2. Mean ± SEM (n=3-4).

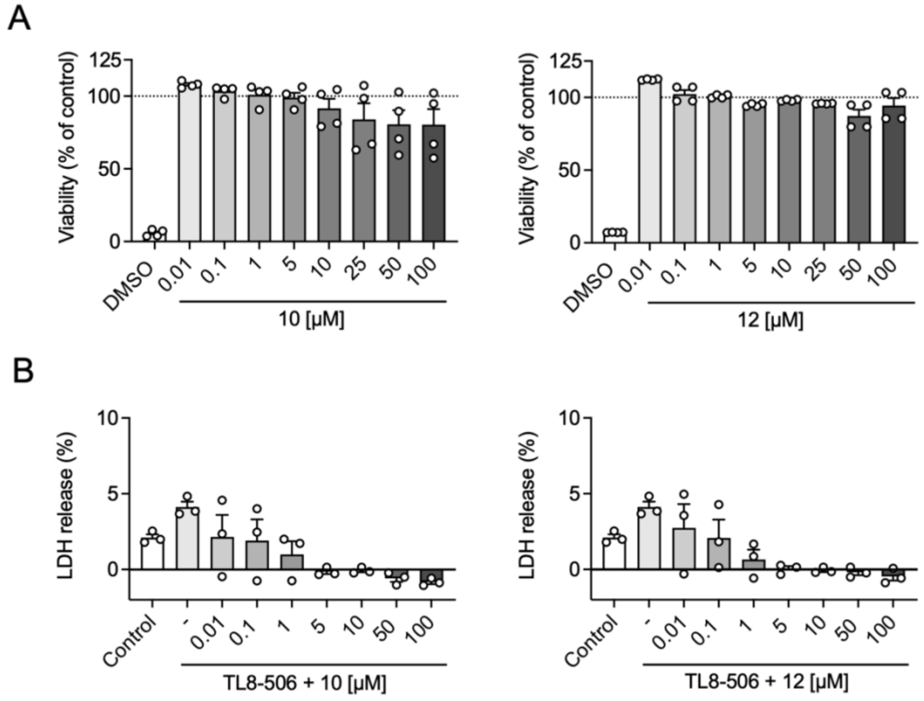

Figure S8. Cell viability of compounds **10** and **12** in THP-1 macrophages and PMBCs.

(A) THP-1 Macrophages were incubated with increasing concentrations of **10** and **12** for 24 h. Cell viability was analyzed using the MTT assay, and normalized to non-stimulated cells (vehicle control). DMSO (10%, v/v) was used as the cytotoxic control. Mean + SEM (n=3). (B) PBMCs were incubated with 0.6 µM TL8-506 or increasing concentrations of **10** and **12** for 24 h. LDH release was determined in cell culture supernatants. Results are shown as percentage of the maximum LDH release. Mean + SEM (n=3-4).

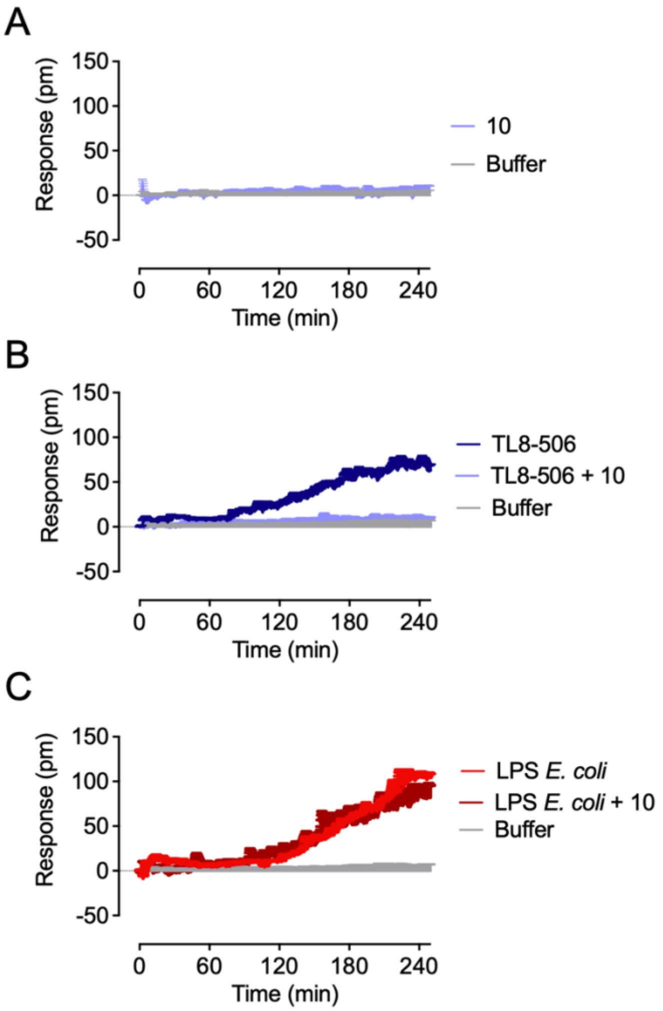

Figure S9. Inhibition of TLR8-dependent activity in THP-1 Dual cells.

Baseline corrected DMR representative recordings of THP-1 Dual MD2-CD14-TLR4 cells stimulated with (A) compound **10** (10 µM) or after 1.5 h preincubation with compound **10** (10 µM) with either (B) TL8-506 (6 µM) or (C) LPS from *E. coli* (1 µg/ml). Calculated pharmacological parameters of the concentration-response curves are depicted in Table S6. Data are mean ± SEM of four independent experiments.

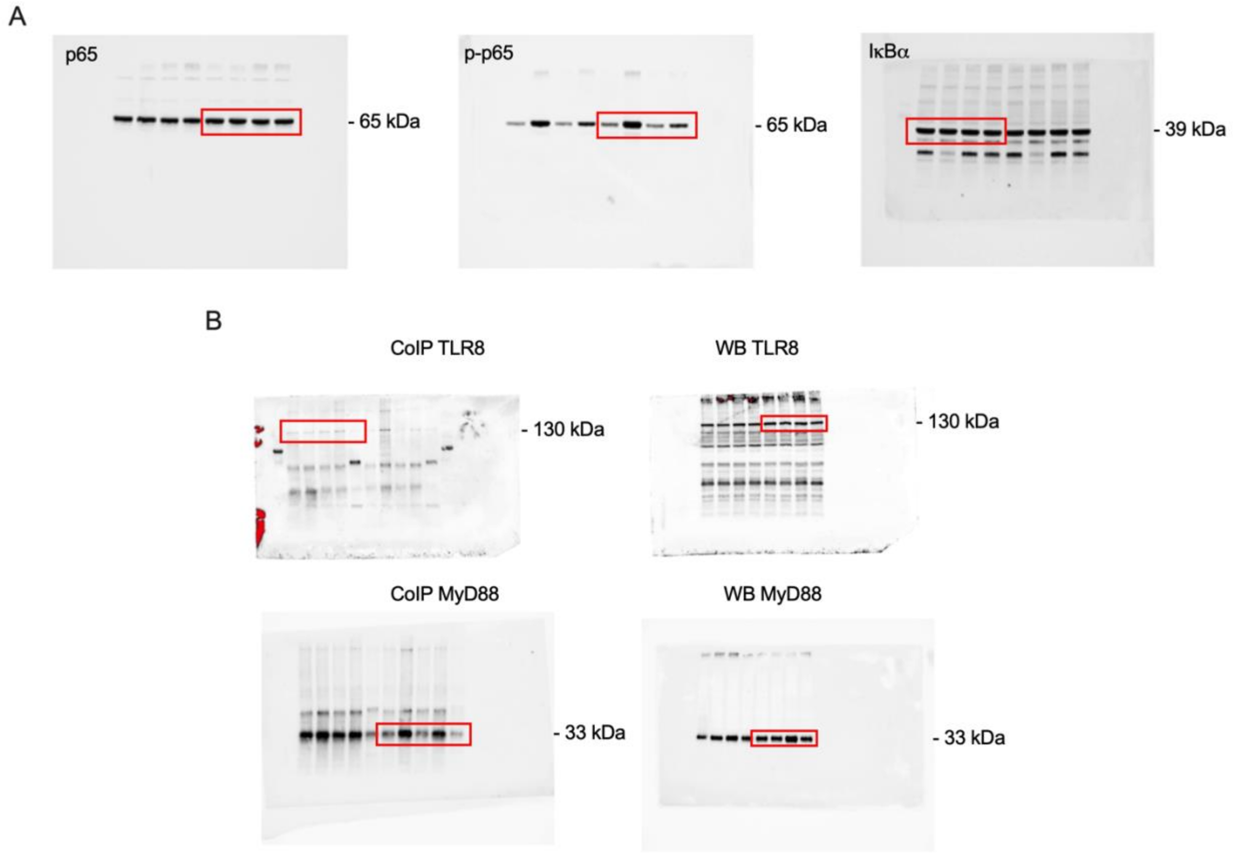

Figure S10. Whole uncropped images of the original western blots.

(A) Complete western blots of NF-κB p65, NF-κB phospho-p65, and IκBα shown in Figure 4E.

(B) Complete western blots of TLR8 and MyD88 after immunoprecipitation shown in Figure 4G.

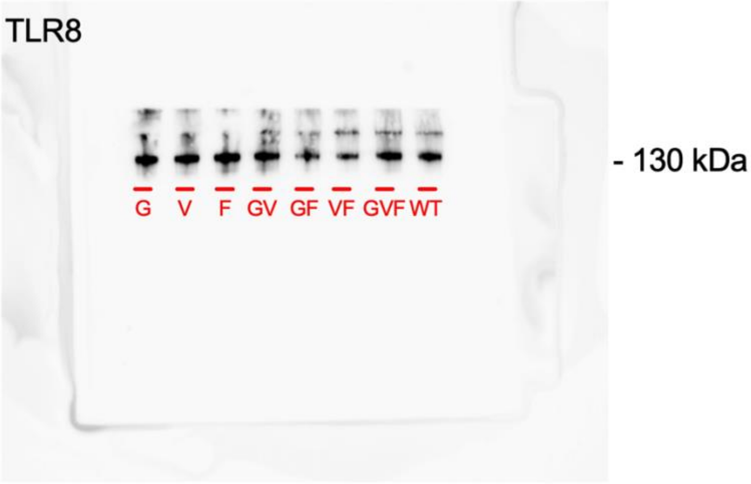

Figure S11. Transfection of mutant and wildtype TLR8 plasmids into HEK293 cells.

HEK293 reporter control cells (Null1) were transfected with mutant (G=Glycin351Prolin, V=Valin378Methionine, F=Phenylalanine495Leucin; GV, GF, VF and GVF=dual- or triple-mutated combinations) or wildtype TLR8 plasmid (WT).

Figure S12. HPLC traces for purchased compounds **1-12**.

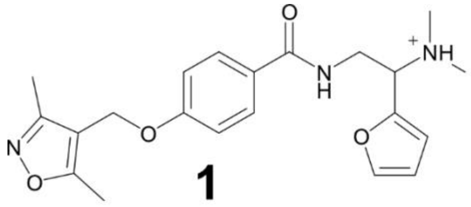

1

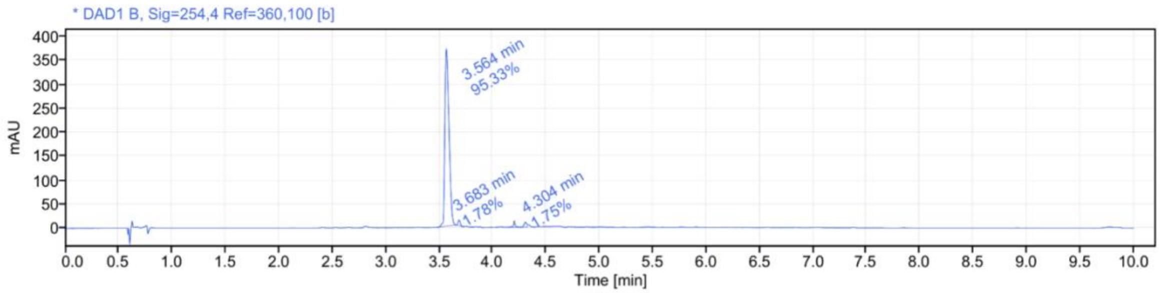

Signal: *DAD1 B, Sig=254,4 Ref= 360,100 [b]

RT	Peak area	Area %
3.564	998.726	95.33
3.683	18.692	1.78
4.156	11.921	1.14
4.304	18.311	1.75
**Sum**	1047.65	

HPLC trace for compound **1**.

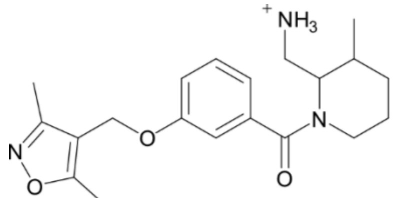

2

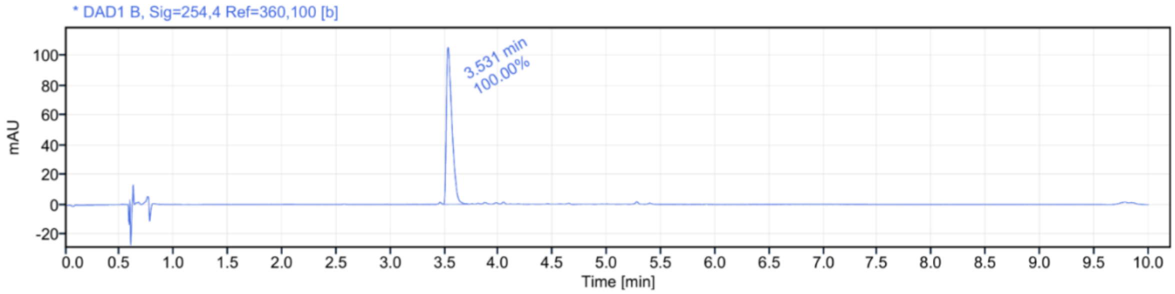

Signal: *DAD1 B, Sig=254,4 Ref= 360,100 [b]

RT	Peak area	Area %
3.531	379.3	100.00
**Sum**	379.3	

HPLC trace for compound **2**.

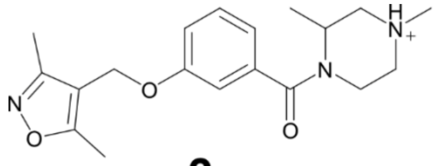

3

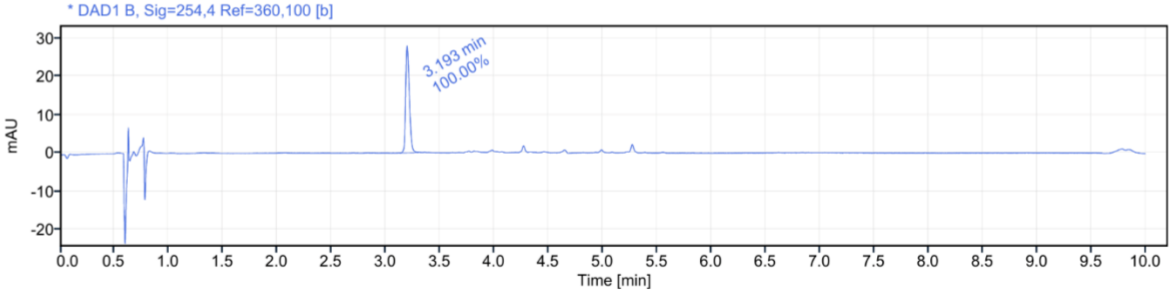

Signal: *DAD1 B, Sig=254,4 Ref= 360,100 [b]

RT	Peak area	Area %
3.193	67.29	100.00
**Sum**	67.29	

HPLC trace for compound **3**.

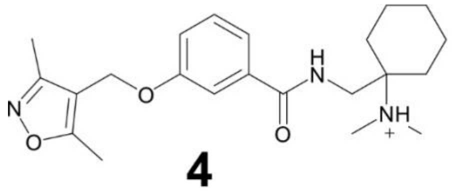

4

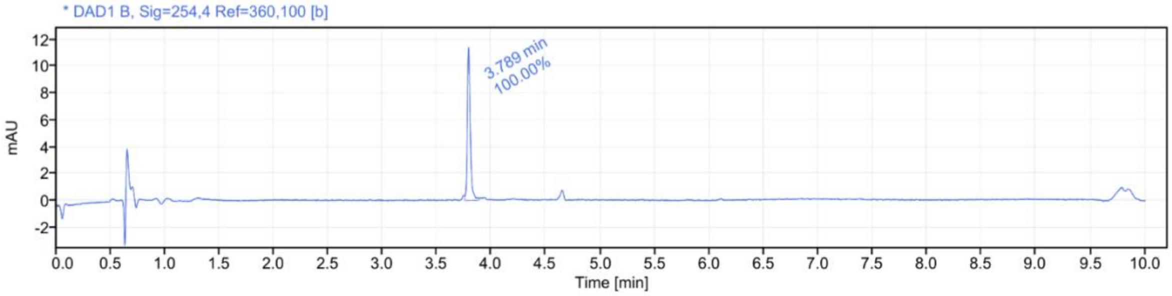

Signal: *DAD1 B, Sig=254,4 Ref= 360,100 [b]

RT	Peak area	Area %
3.789	21.72	100.00
**Sum**	21.72	

HPLC trace for compound **4**.

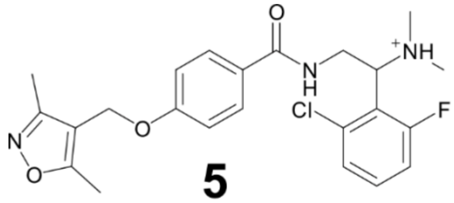

5

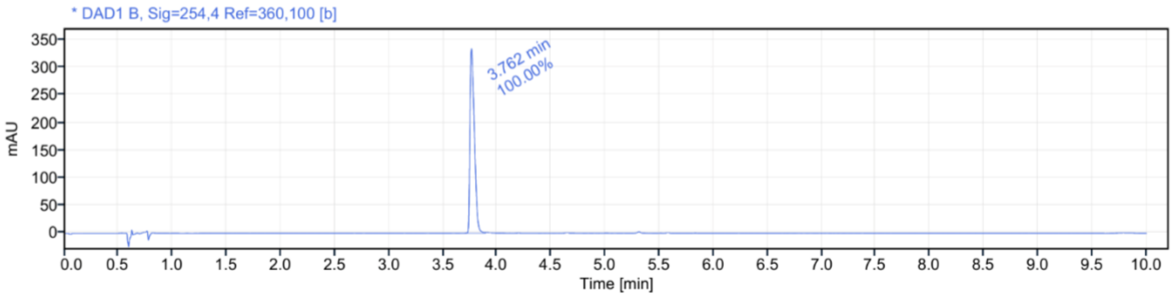

Signal: *DAD1 B, Sig=254,4 Ref= 360,100 [b]

**RT**	**Peak area**	**Area %**
3.762	954.9	100.00
**Sum**	954.9	

HPLC trace for compound **5**.

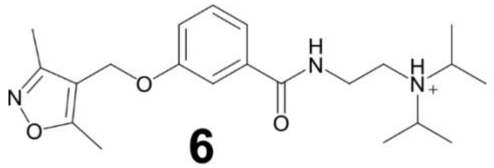

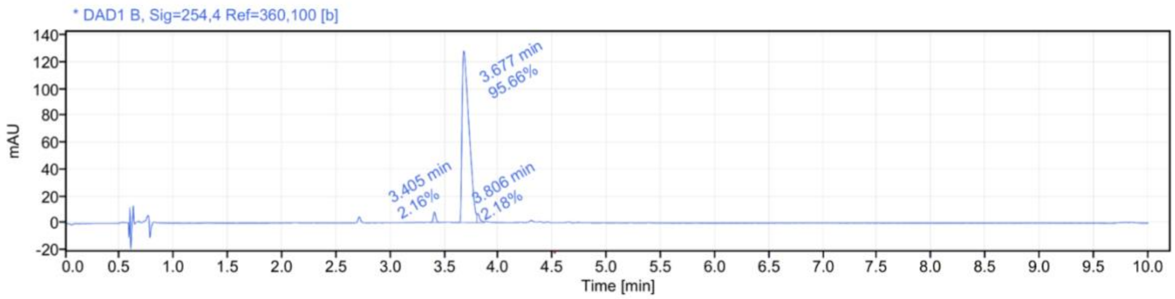

Signal: *DAD1 B, Sig=254,4 Ref= 360,100 [b]

RT	Peak area	Area %
3.405	12.97	2.16
3.677	574.1	95.66
3.806	13.05	2.18
**Sum**	600.12	

HPLC trace for compound **6**.

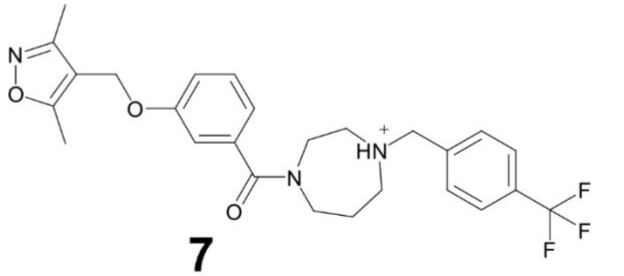

7

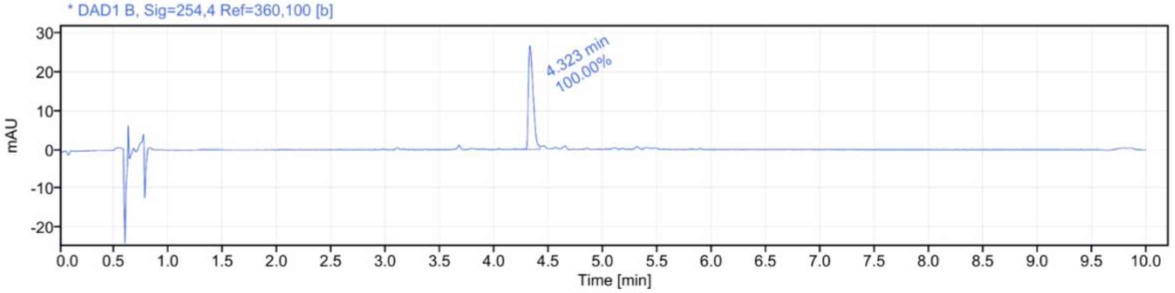

Signal: *DAD1 B, Sig=254,4 Ref= 360,100 [b]

RT	Peak area	Area %
4.323	81.04	100.00
**Sum**	81.04	

HPLC trace for compound **7**.

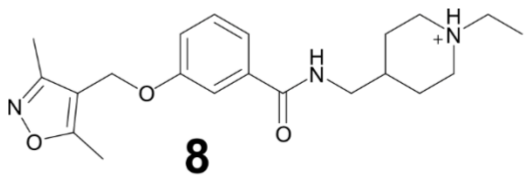

8

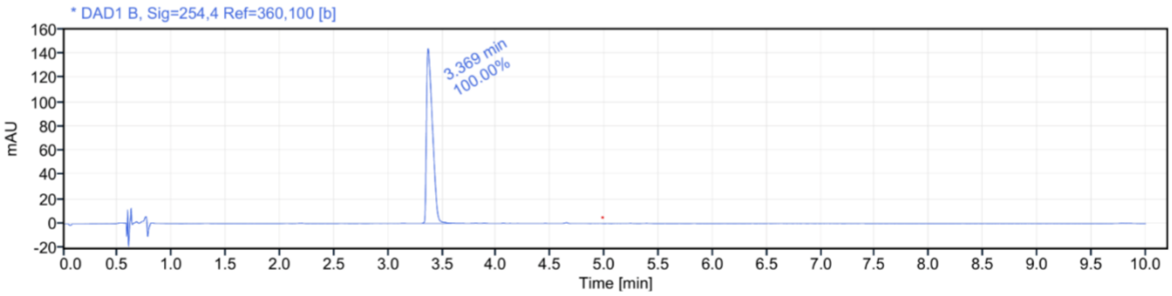

Signal: *DAD1 B, Sig=254,4 Ref= 360,100 [b]

RT	Peak area	Area %
3.369	554.1	100.00
**Sum**	554.1	

HPLC trace for compound **8**.

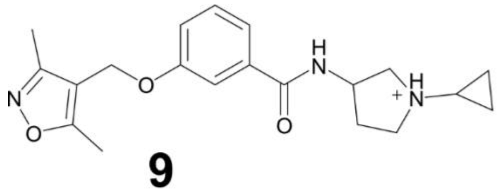

9

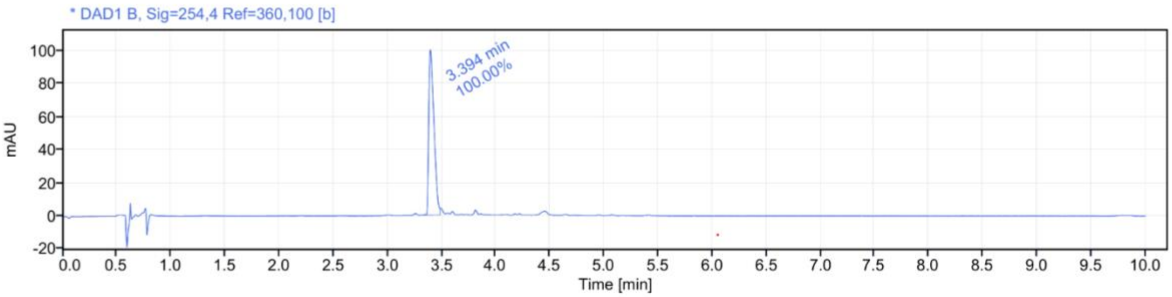

Signal:	*DAD1 B, Sig=254,4 Ref= 360,100 [b]	
**RT**	**Peak area**	**Area %**
3.394	337.2	100.00
**Sum**	337.2	

HPLC trace for compound **9**.

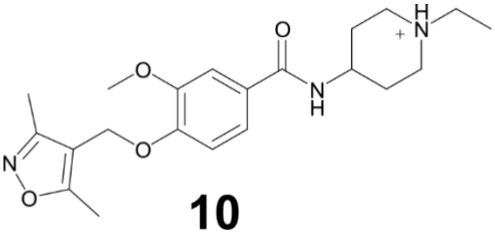

10

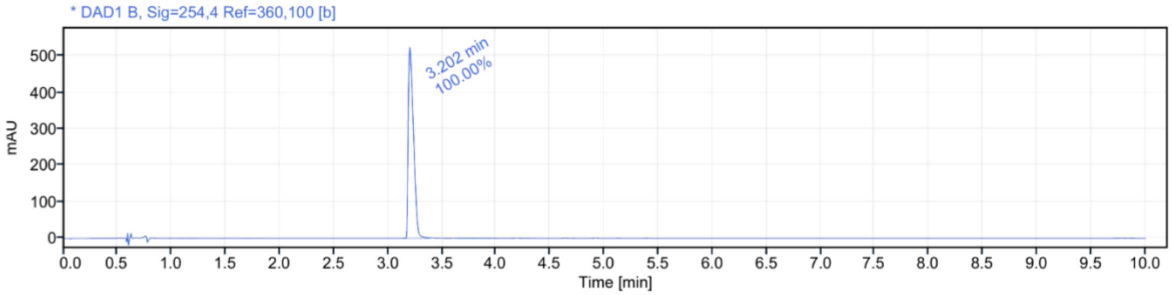

Signal: *DAD1 B, Sig=254,4 Ref= 360,100 [b]

RT	Peak area	Area %
3.202	1768	100.00
**Sum**	1768	

HPLC trace for lead compound **10**.

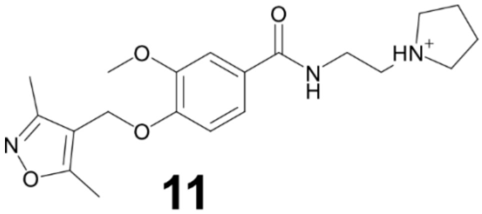

11

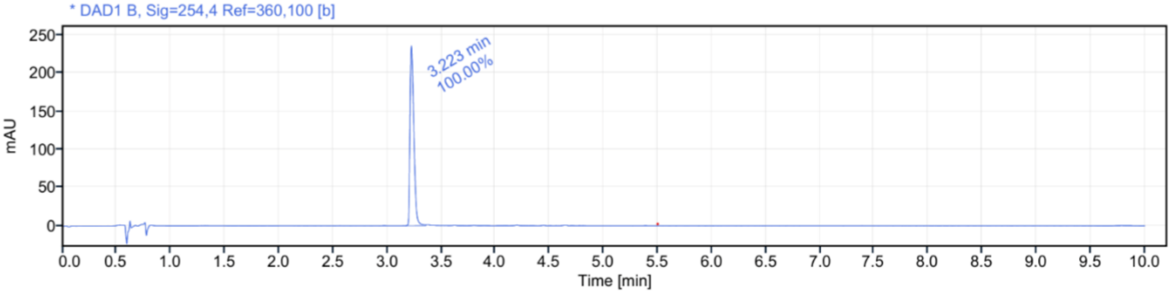

Signal:	*DAD1 B, Sig=254,4 Ref= 360,100 [b]	
**RT**	**Peak area**	**Area %**
3.223	584.0	100.00
**Sum**	584.0	

HPLC trace for compound **11**.

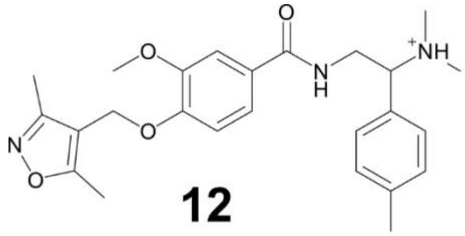

12

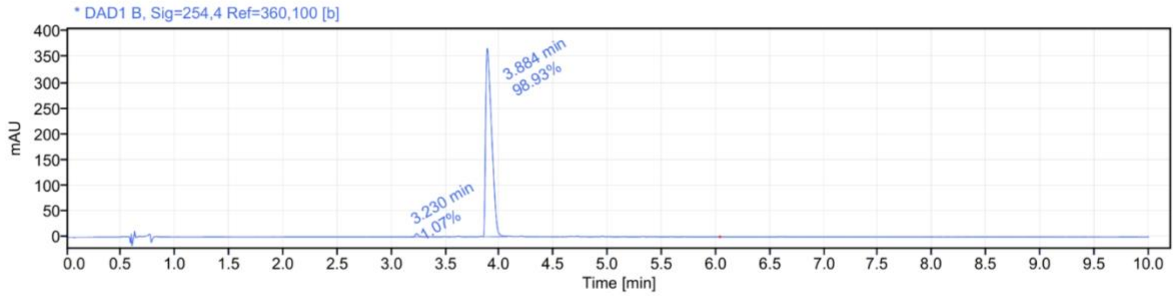

Signal: *DAD1 B, Sig=254,4 Ref= 360,100 [b]

RT	Peak area	Area %
3.230	15.48	1.07
3.884	1429	98.93
**Sum**	1444.48	

HPLC trace for compound **12**.

Figure S13. ^{1}H NMR spectra for purchased active compounds **1**, **10-12**.

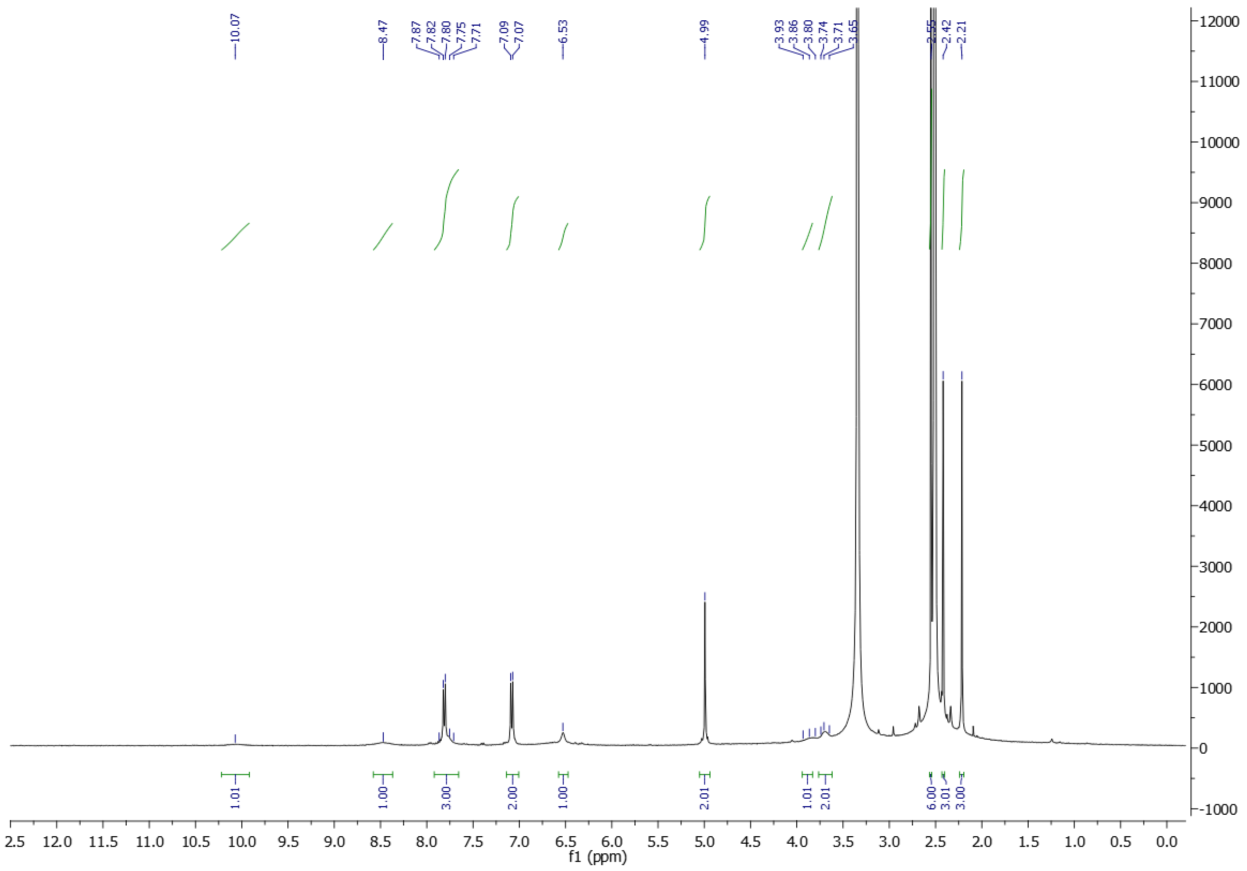

^{1}H NMR for compound **1**.

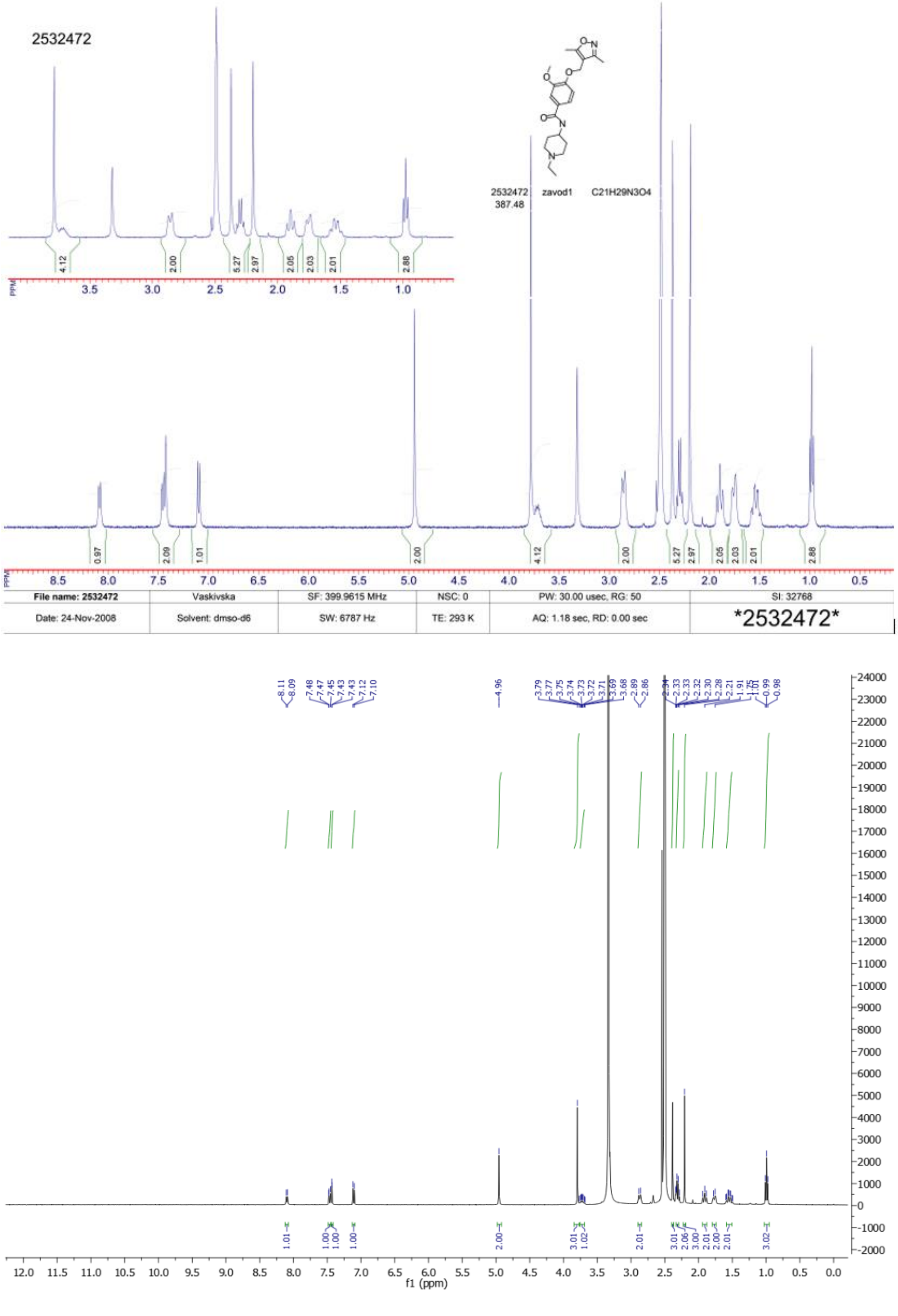

^{1}H NMR for lead compound **10** (top: provided by vendor, bottom: in-house analysis).

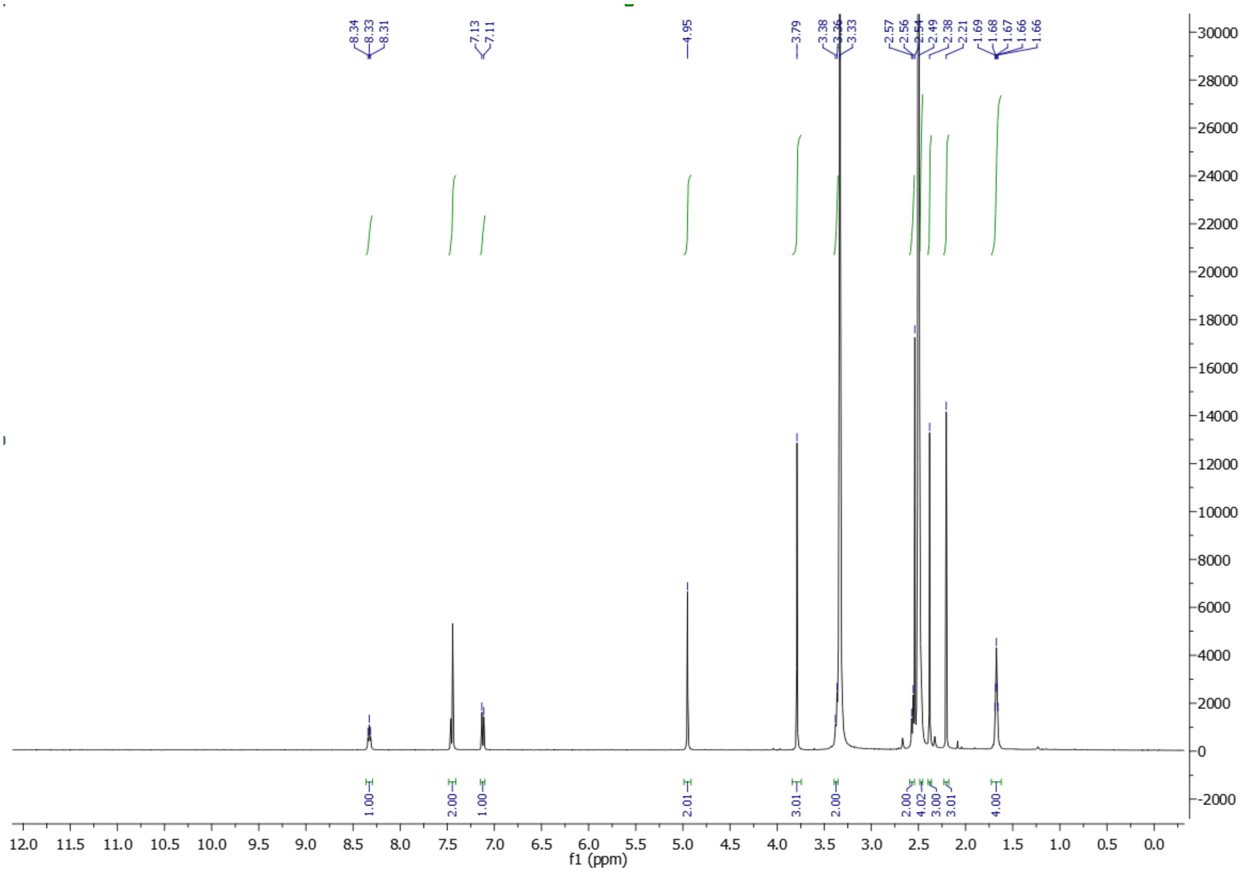

^{1}H NMR for compound **11**.

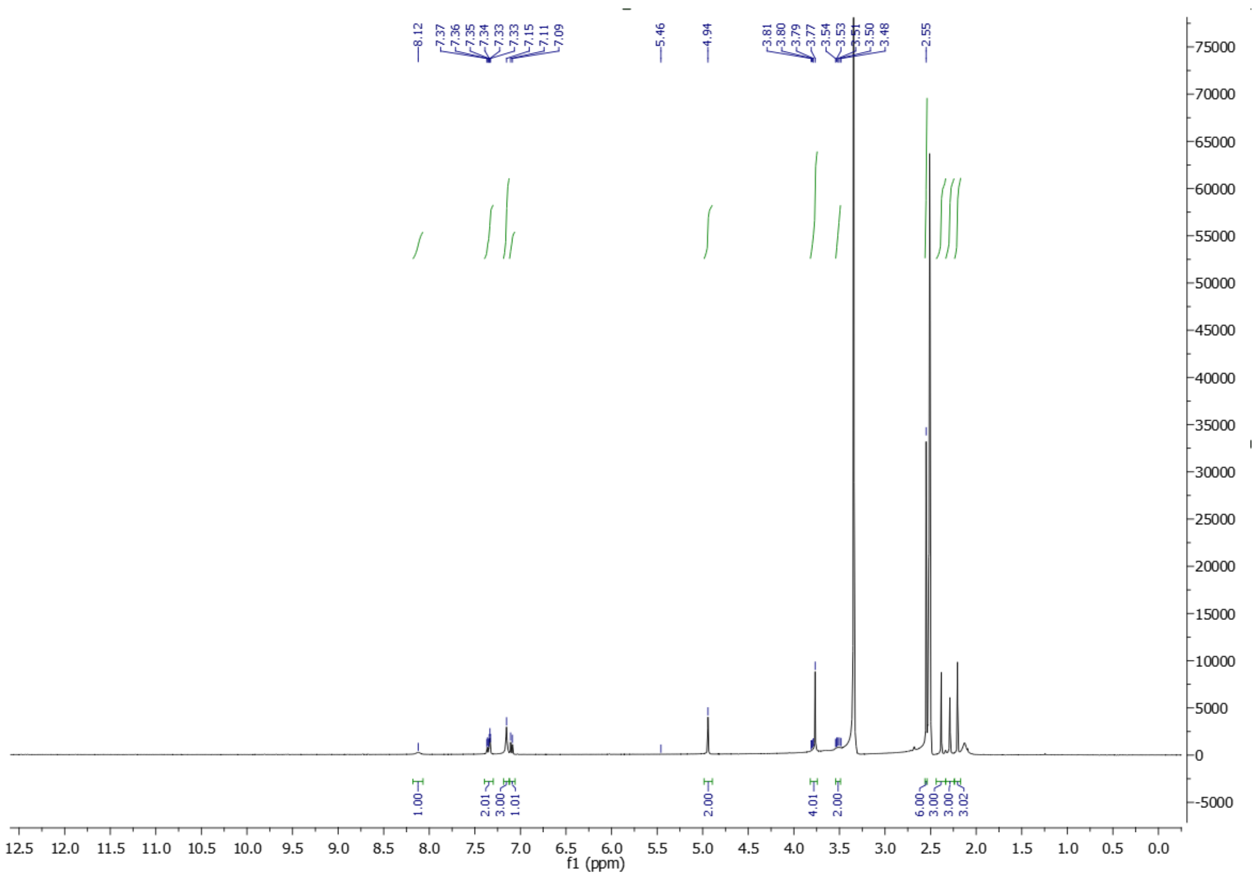

^{1}H NMR for compound **12**.

Figure S14. ^{1}H NMR, ^{13}C NMR and HPLC traces for synthesized active compounds.

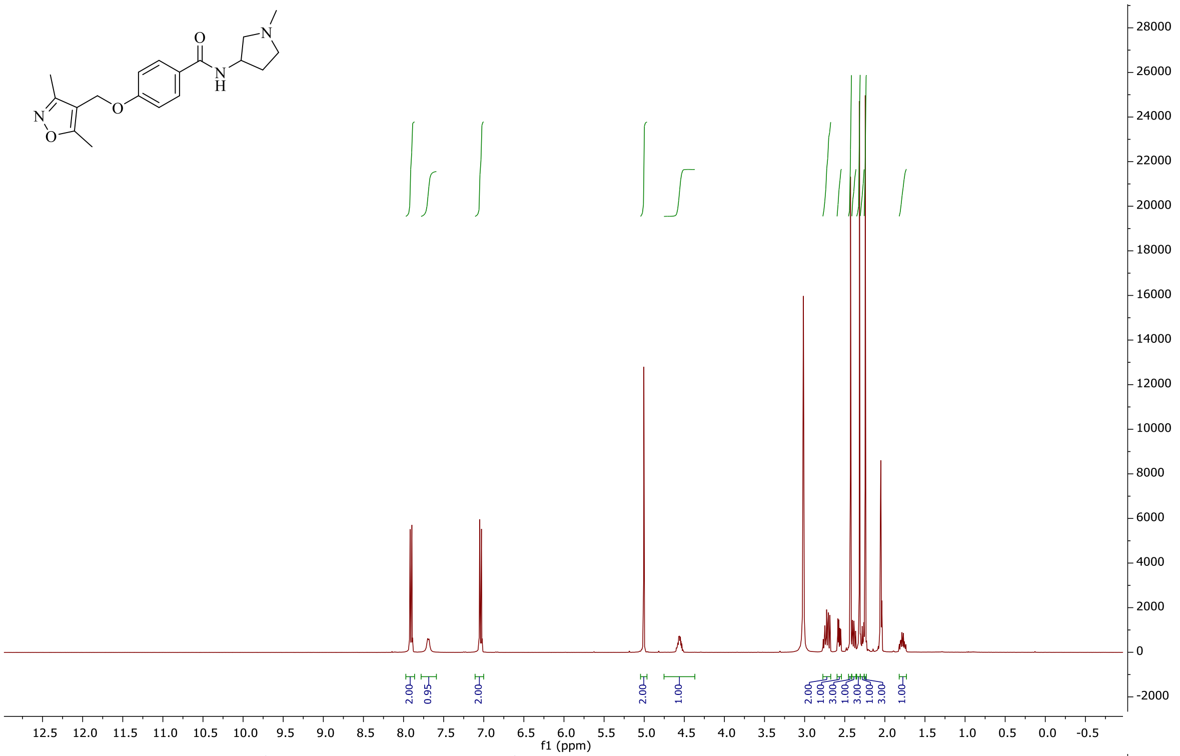

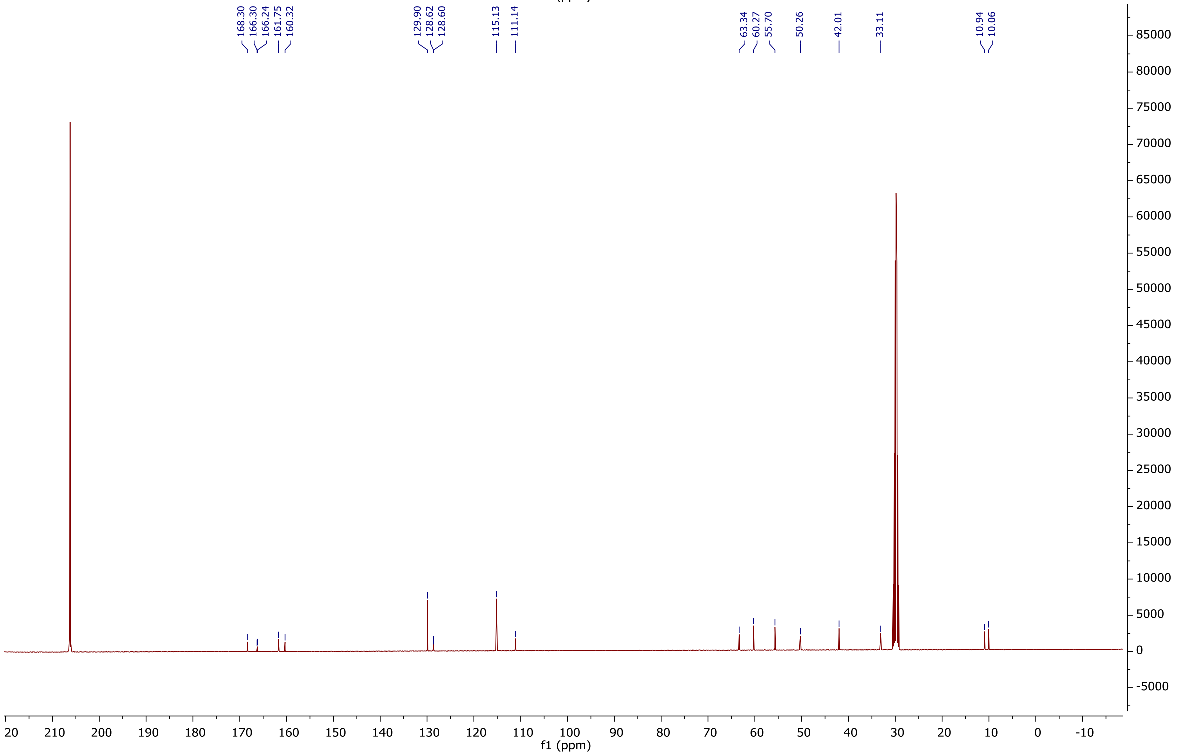

^{1}H and ^{13}C NMR for compound **19**.

Chromatogram

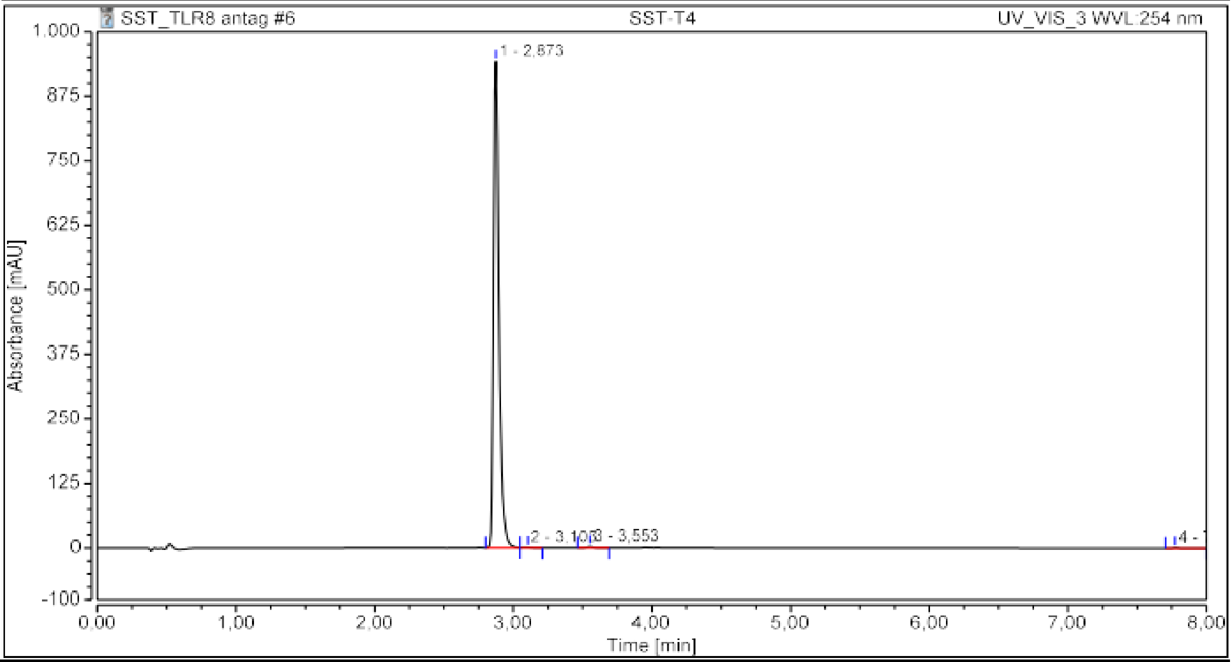

Integration Results

No.	Peak Name	Retention Time min	Area mAU*min	Height mAU	Relative Area %	Relative Height %	Amount n.a.
1		2,873	43,304	942,087	99,38	99,48	n.a.
2		3,103	0,079	1,027	0,18	0,11	n.a.
3		3,553	0,124	2,671	0,28	0,28	n.a.
4		7,773	0,066	1,258	0,15	0,13	n.a.
**Total:**			**43,573**	**947,042**	**100,00**	**100,00**	

HPLC trace for compound **19**.

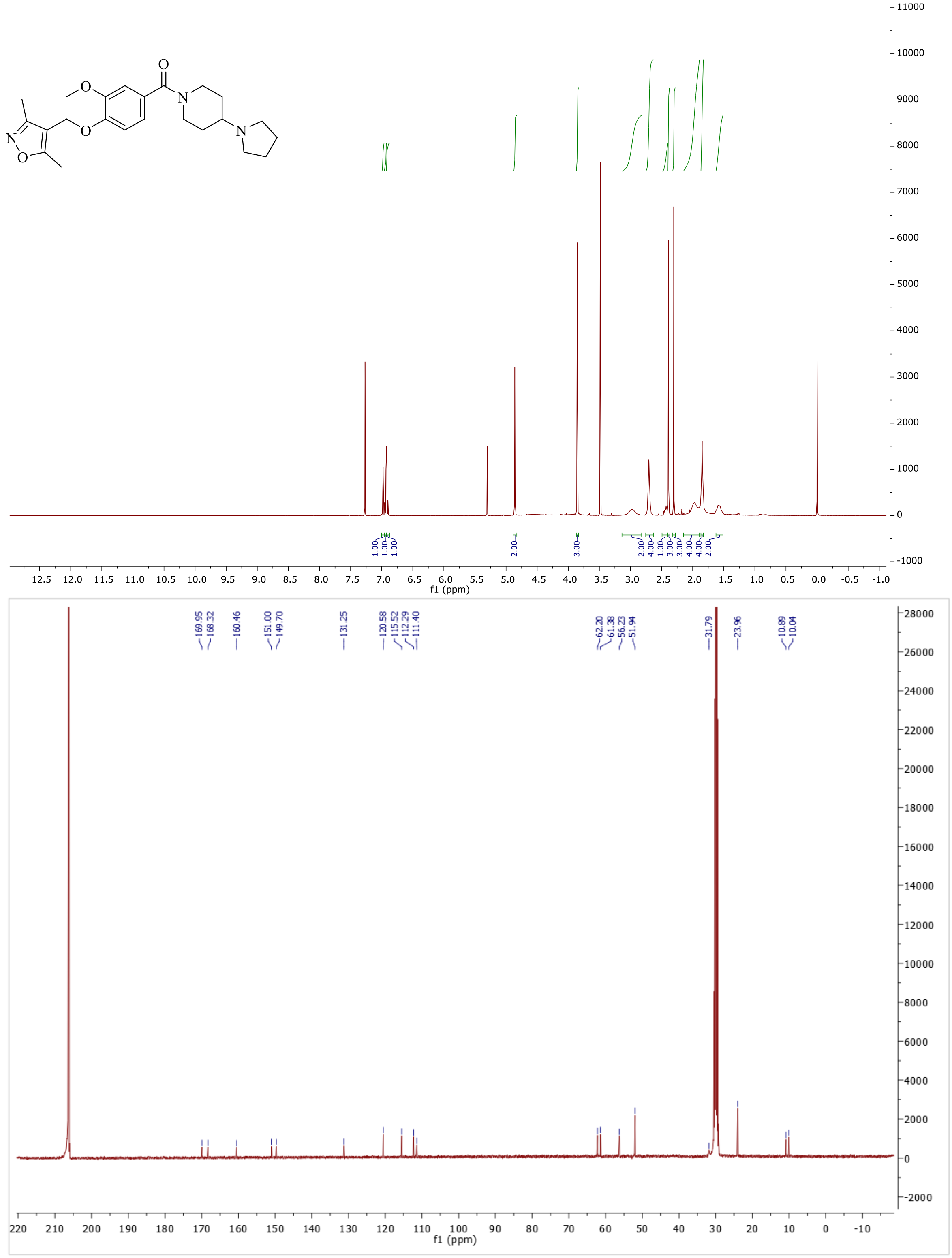

^{1}H and ^{13}C NMR for compound **24**.

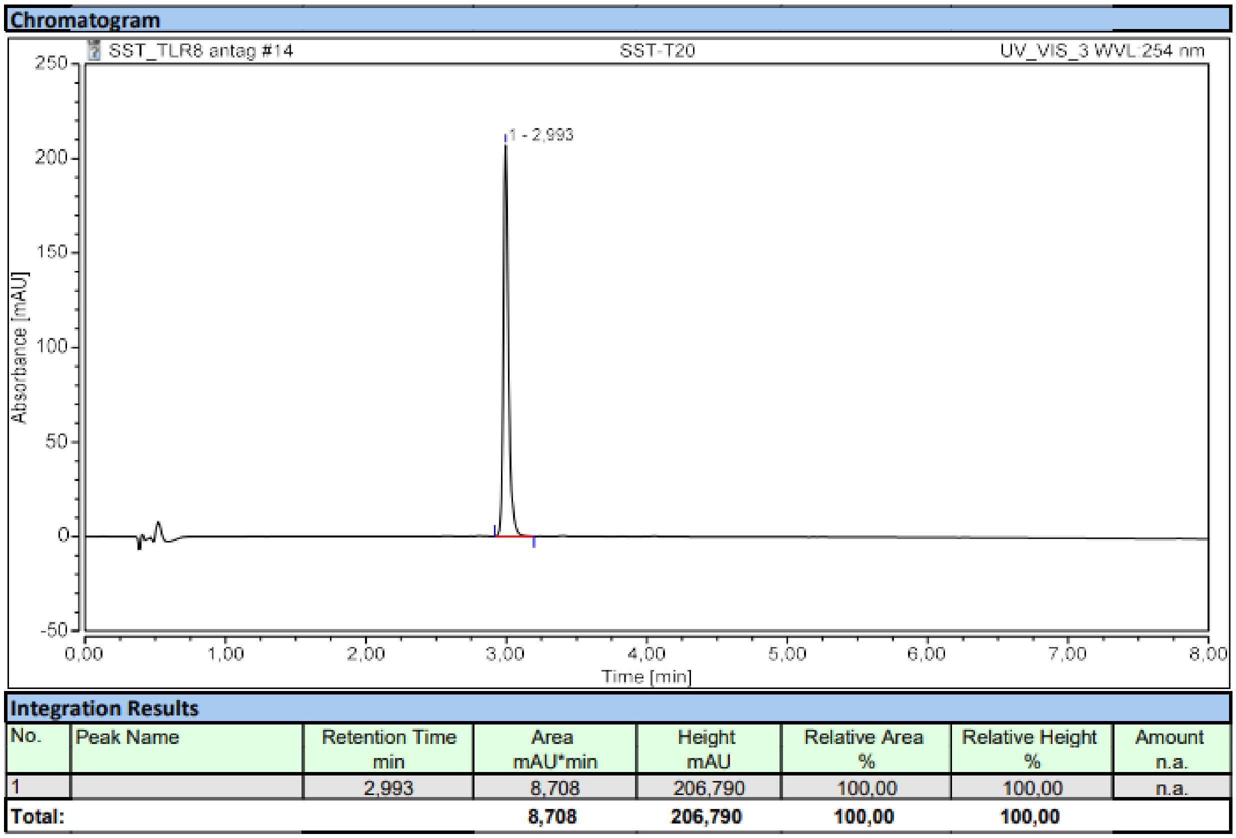

Integration Results

No.	Peak Name	Retention Time min	Area mAU*min	Height mAU	Relative Area %	Relative Height %	Amount n.a.
1		2,993	8,708	206,790	100,00	100,00	n.a.
**Total:**			**8,708**	**206,790**	**100,00**	**100,00**	

HPLC trace for compound **24**.

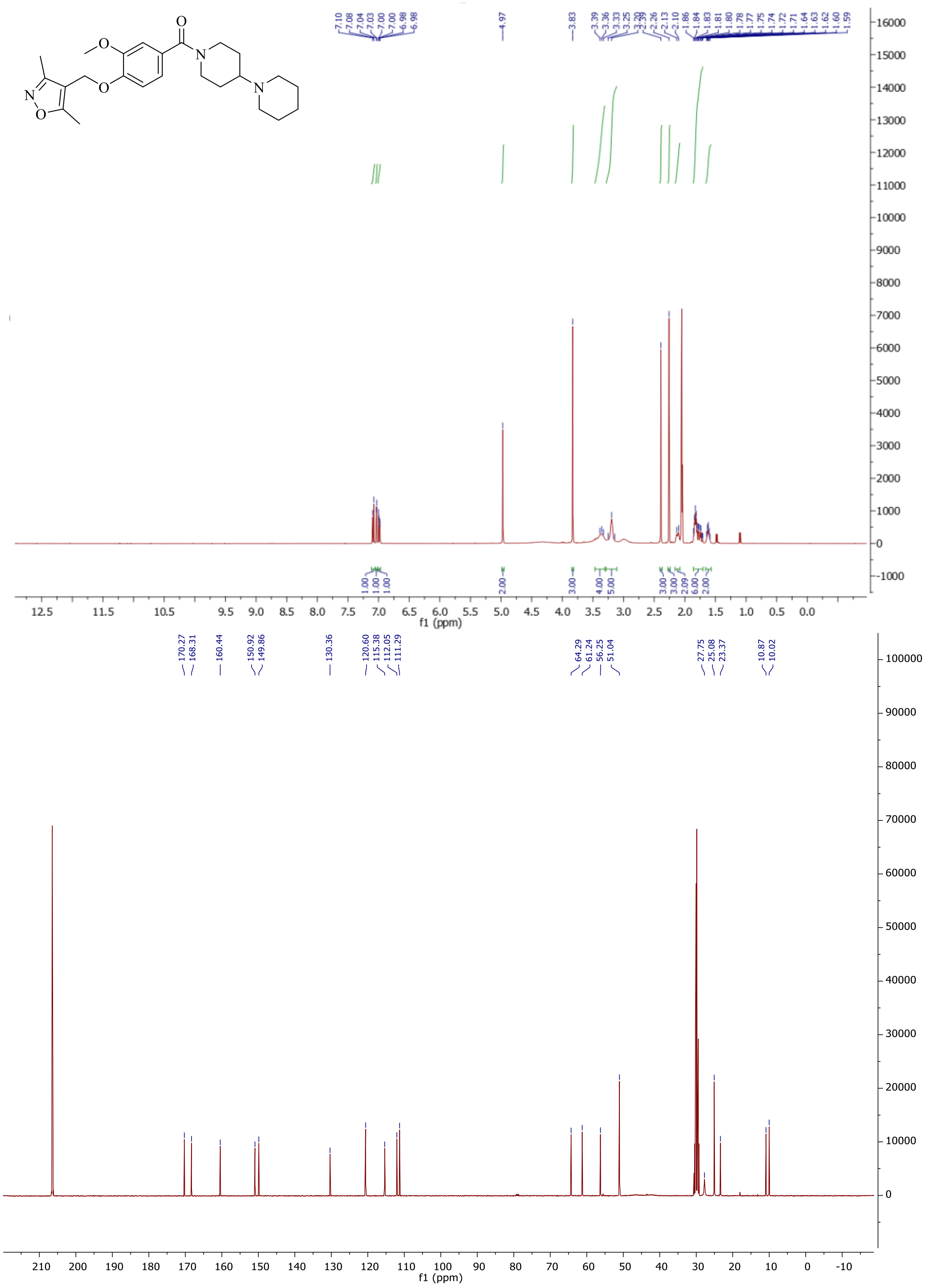

^{1}H and ^{13}C NMR for compound **26**.

Chromatogram

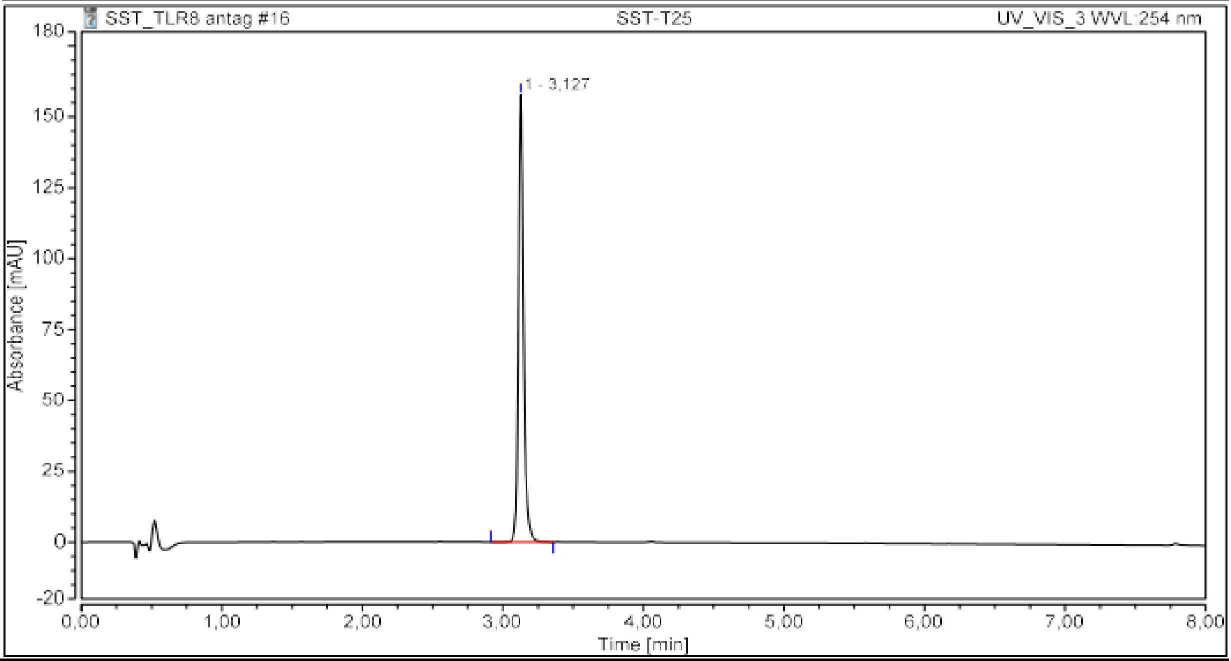

Integration Results

No.	Peak Name	Retention Time min	Area mAU*min	Height mAU	Relative Area %	Relative Height %	Amount n.a.
1		3,127	6,716	157,672	100,00	100,00	n.a.
**Total:**			**6,716**	**157,672**	**100,00**	**100,00**	

HPLC trace for compound **26**.

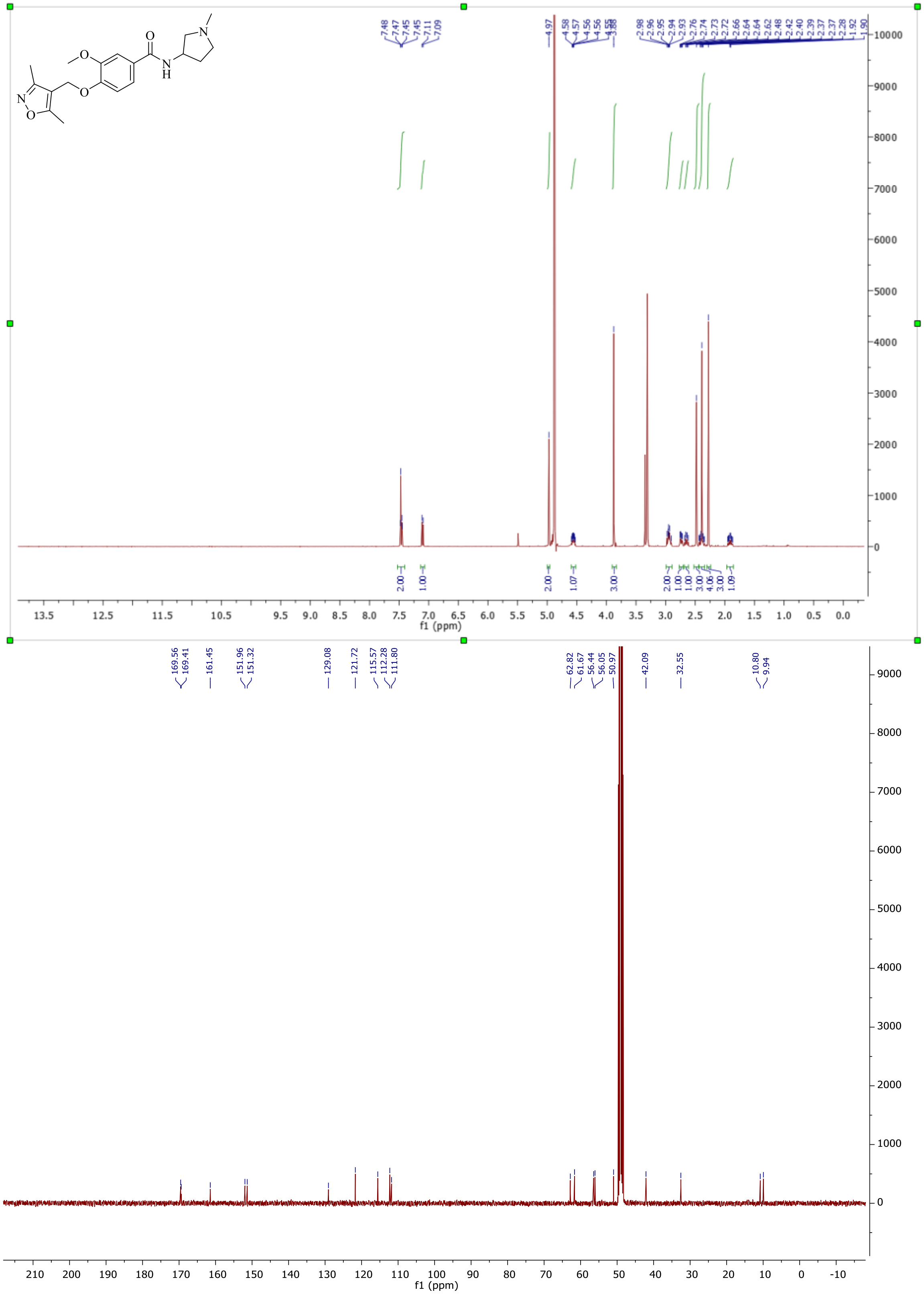

^{1}H and ^{13}C NMR for compound **27**.

Chromatogram

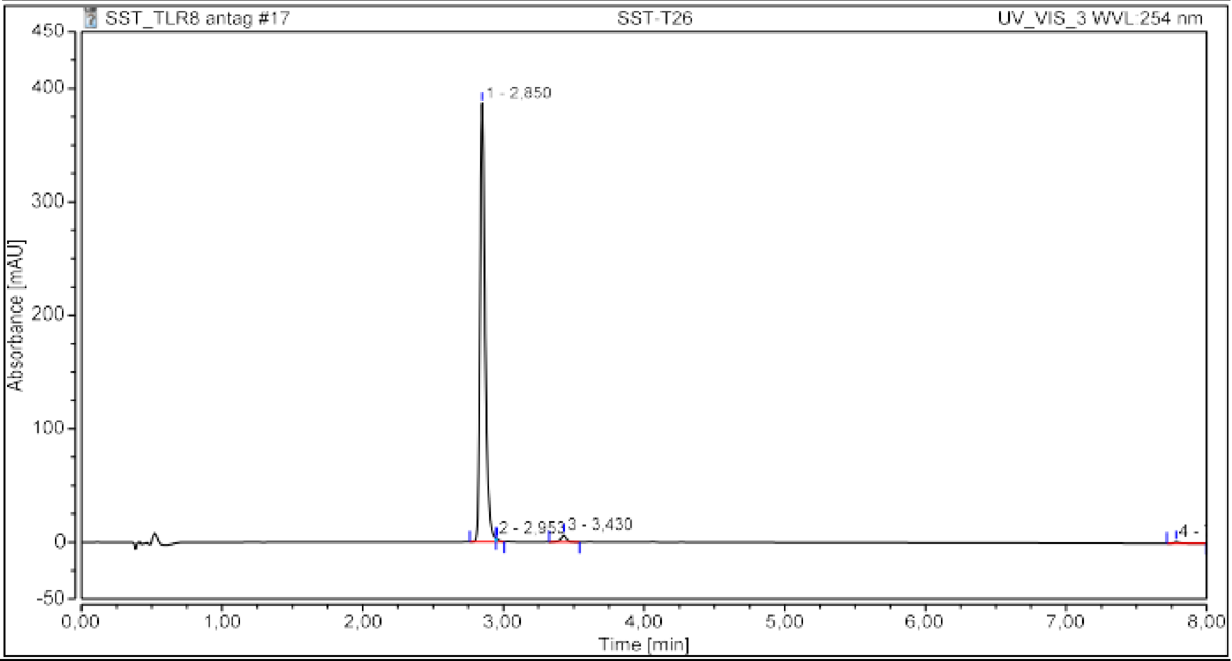

Integration Results

No.	Peak Name	Retention Time min	Area mAU*min	Height mAU	Relative Area %	Relative Height %	Amount n.a.
1		2,850	15,950	386,276	97,63	97,55	n.a.
2		2,953	0,056	2,426	0,35	0,61	n.a.
3		3,430	0,266	6,085	1,63	1,54	n.a.
4		7,783	0,065	1,181	0,40	0,30	n.a.
**Total:**			**16,338**	**395,968**	**100,00**	**100,00**	

HPLC trace for compound **27**.

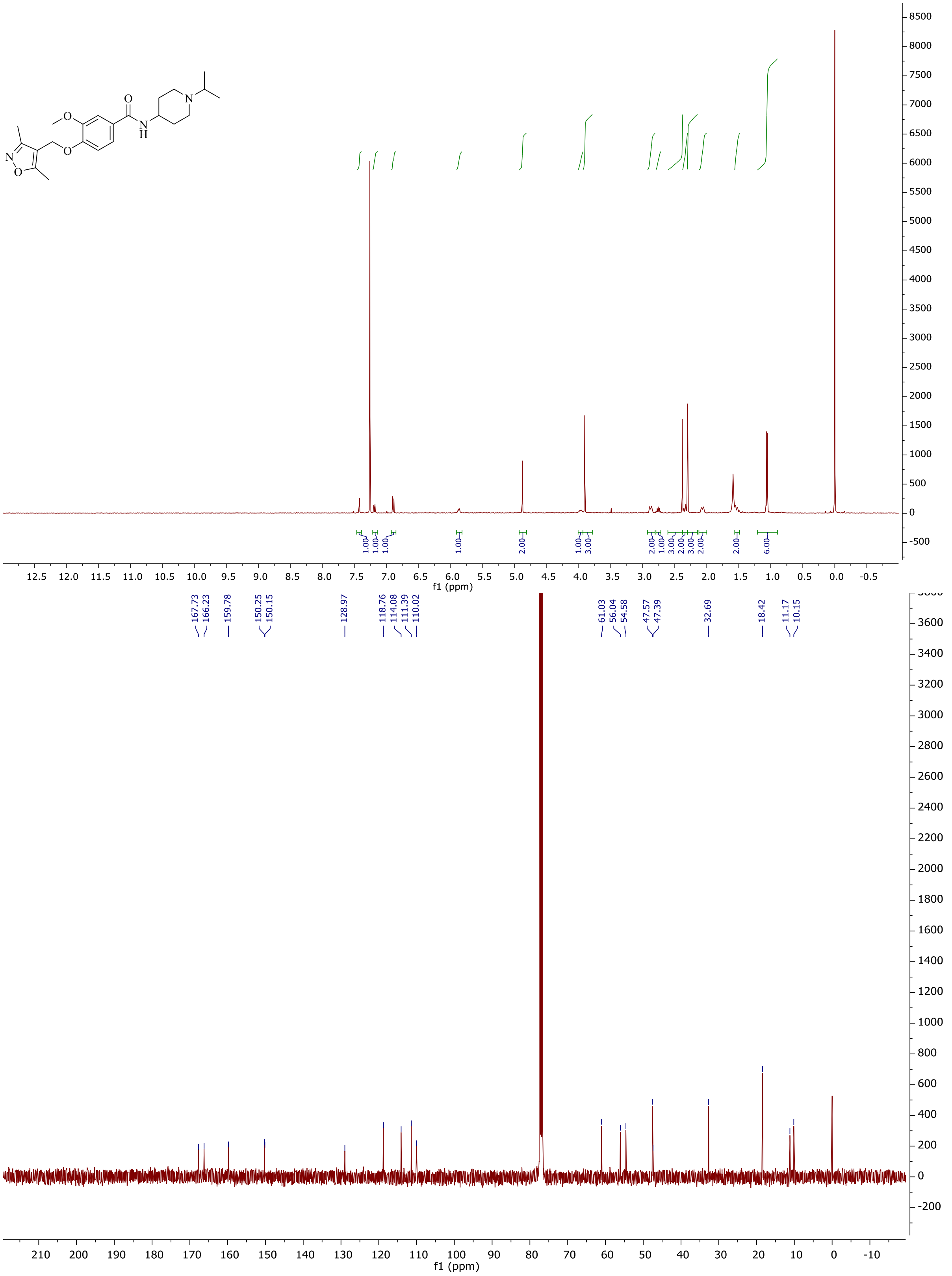

^{1}H and ^{13}C NMR for compound **28**.

Chromatogram

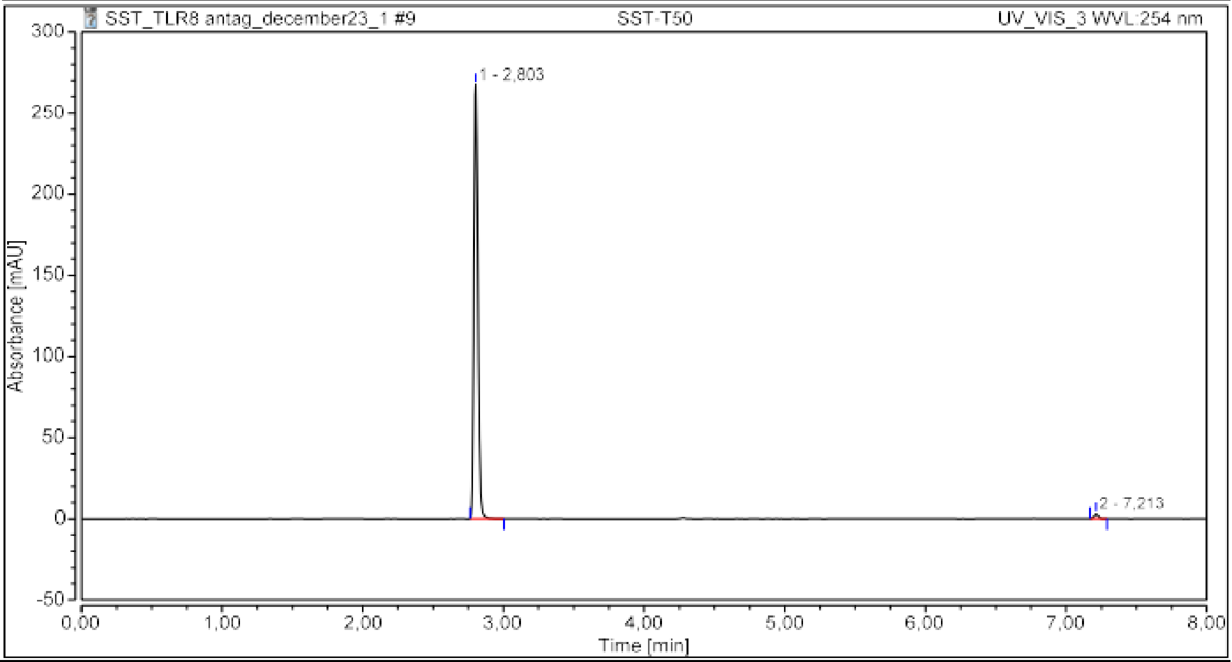

Integration Results

No.	Peak Name	Retention Time min	Area mAU*min	Height mAU	Relative Area %	Relative Height %	Amount n.a.
1		2,803	8,987	267,233	98,91	98,88	n.a.
2		7,213	0,099	3,030	1,09	1,12	n.a.
**Total:**			**9,086**	**270,263**	**100,00**	**100,00**	

HPLC trace for compound **28**.

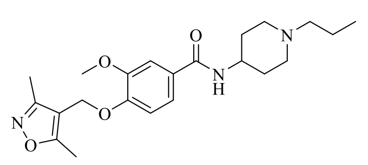

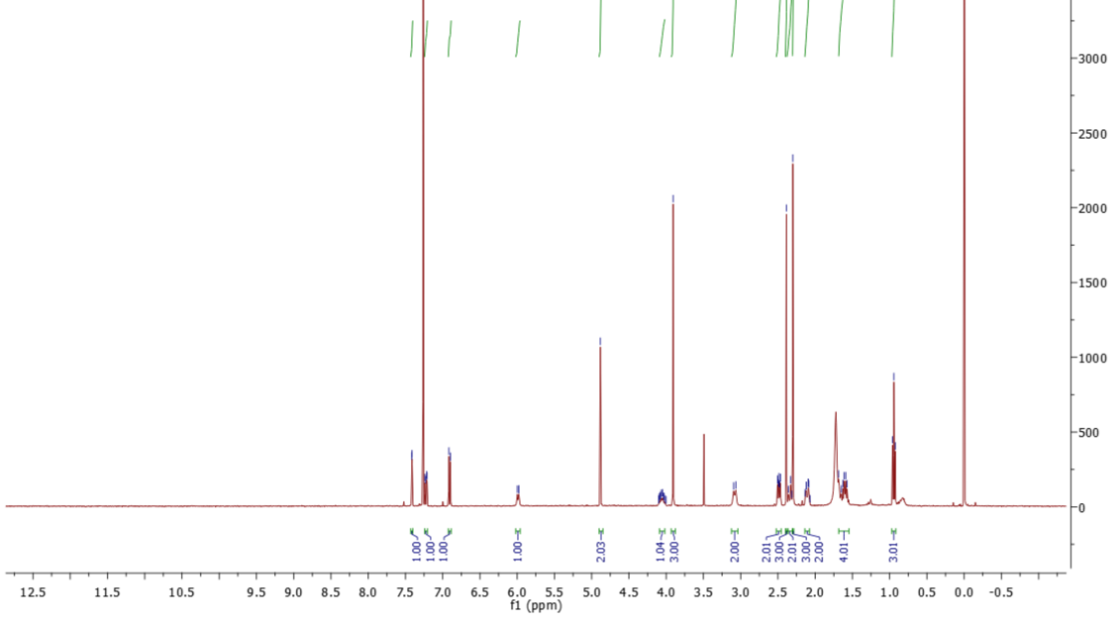

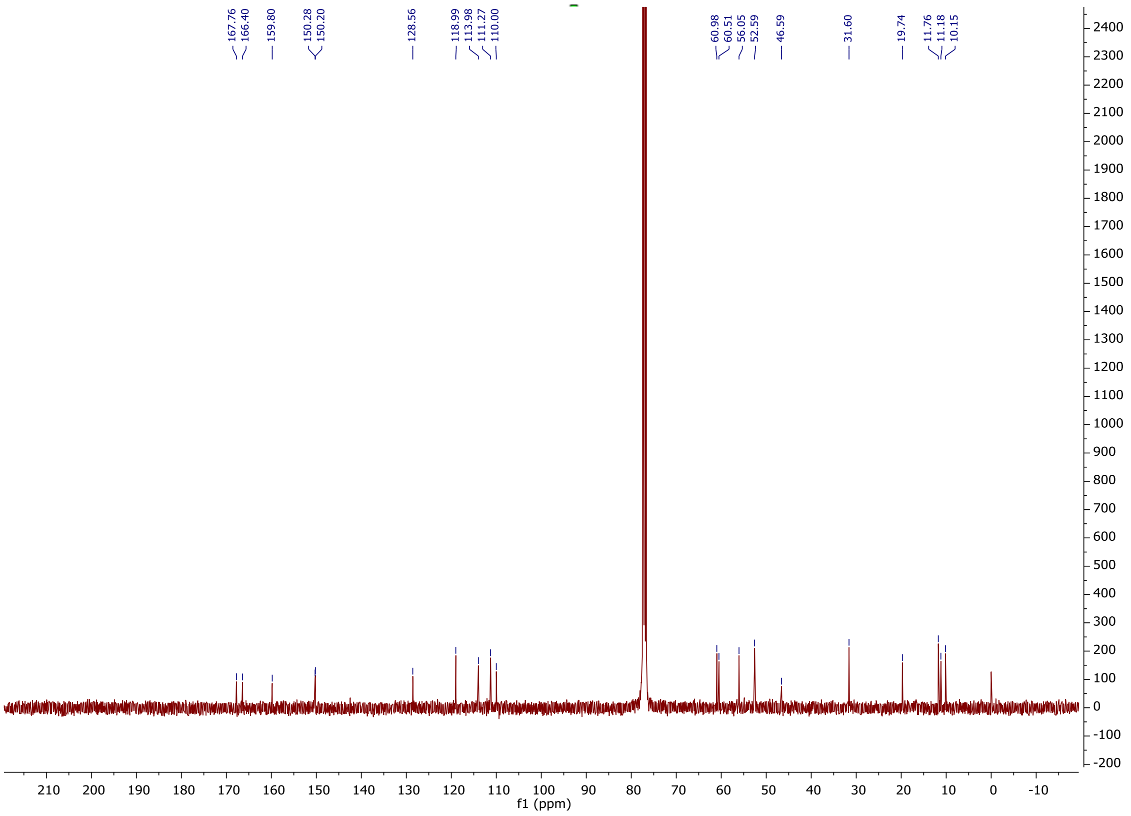

^{1}H and ^{13}C NMR for compound **31**.

Chromatogram

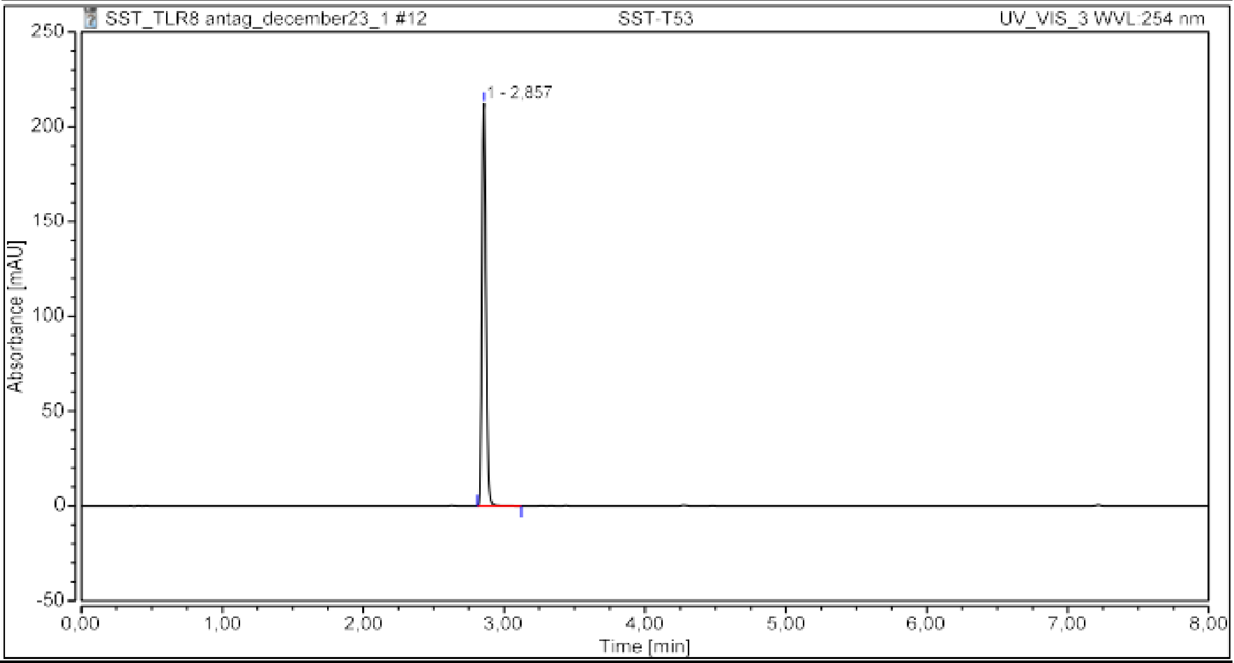

Integration Results

No.	Peak Name	Retention Time min	Area mAU*min	Height mAU	Relative Area %	Relative Height %	Amount n.a.
1		2,857	7,069	212,251	100,00	100,00	n.a.
**Total:**			**7,069**	**212,251**	**100,00**	**100,00**	

HPLC trace for compound **31**.

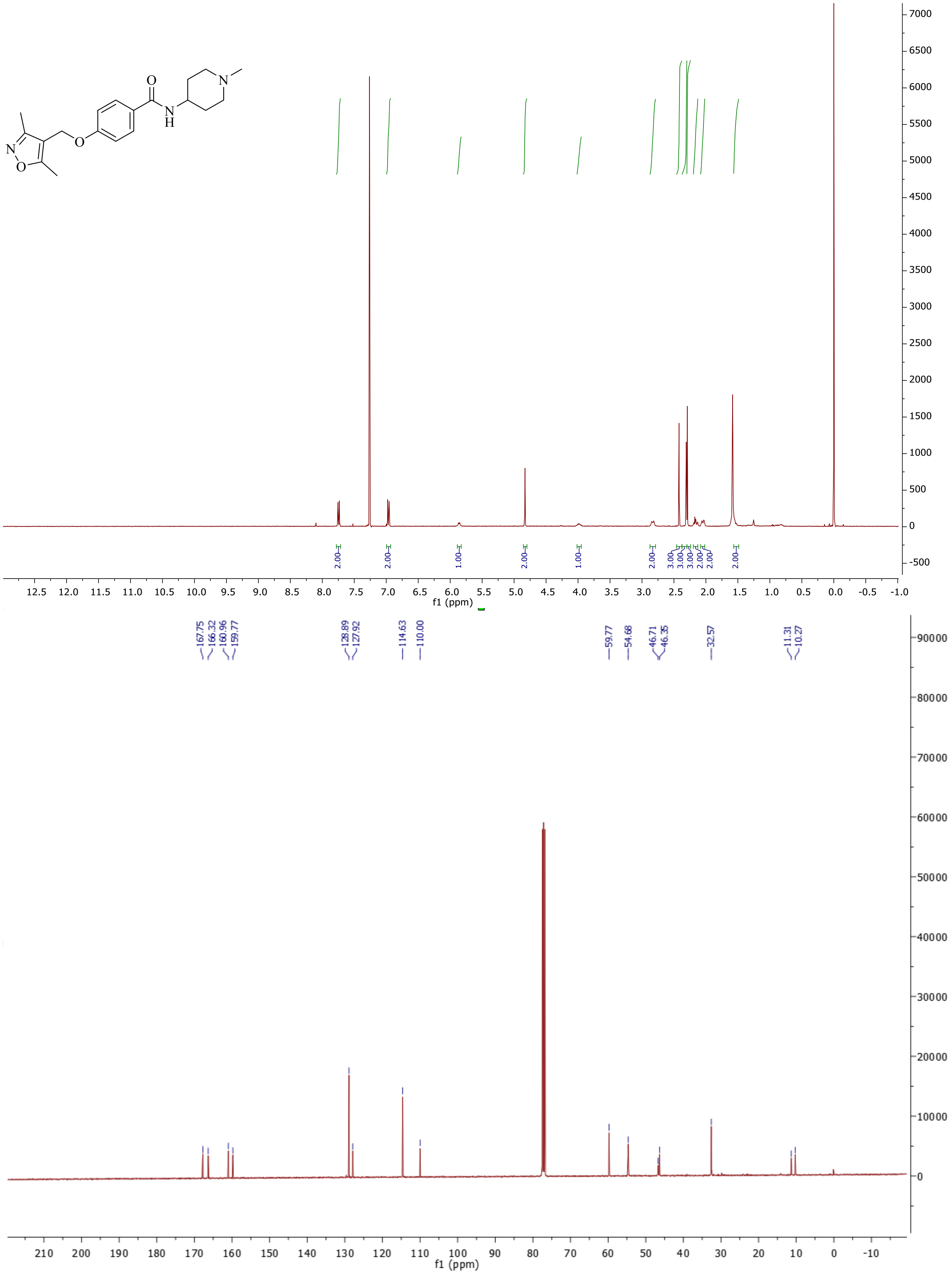

^{1}H and ^{13}C NMR for compound **34**.

Chromatogram

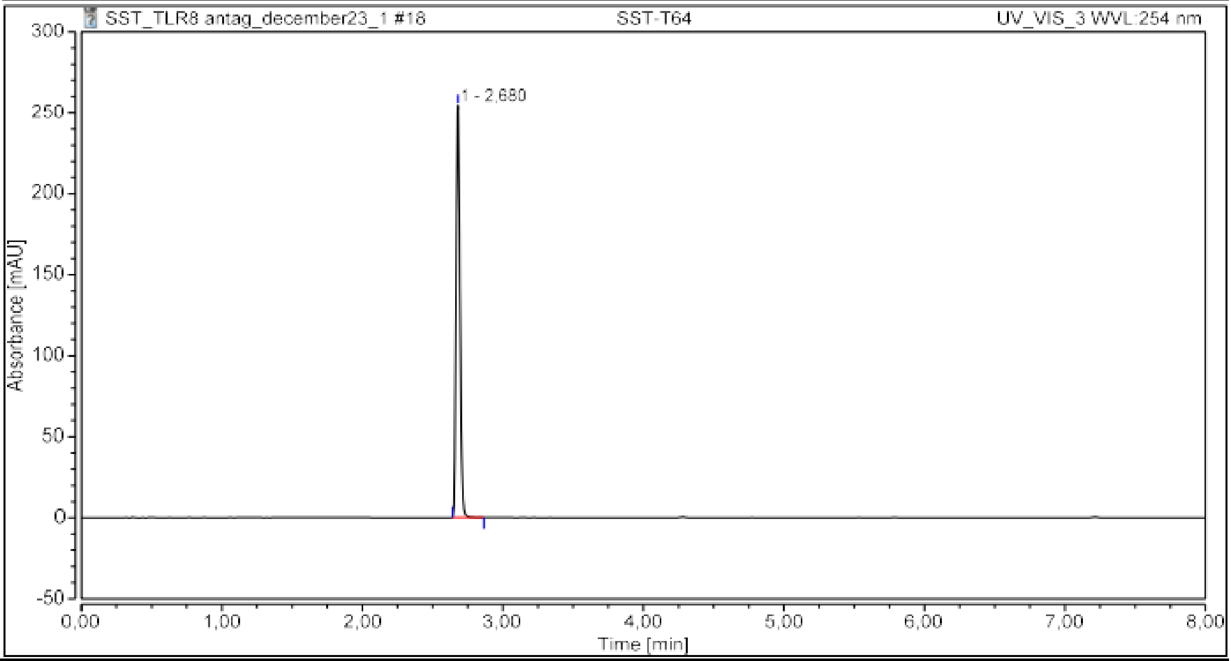

Integration Results

No.	Peak Name	Retention Time min	Area mAU*min	Height mAU	Relative Area %	Relative Height %	Amount n.a.
1		2,680	8,217	254,166	100,00	100,00	n.a.
**Total:**			**8,217**	**254,166**	**100,00**	**100,00**	

HPLC trace for compound **34**.

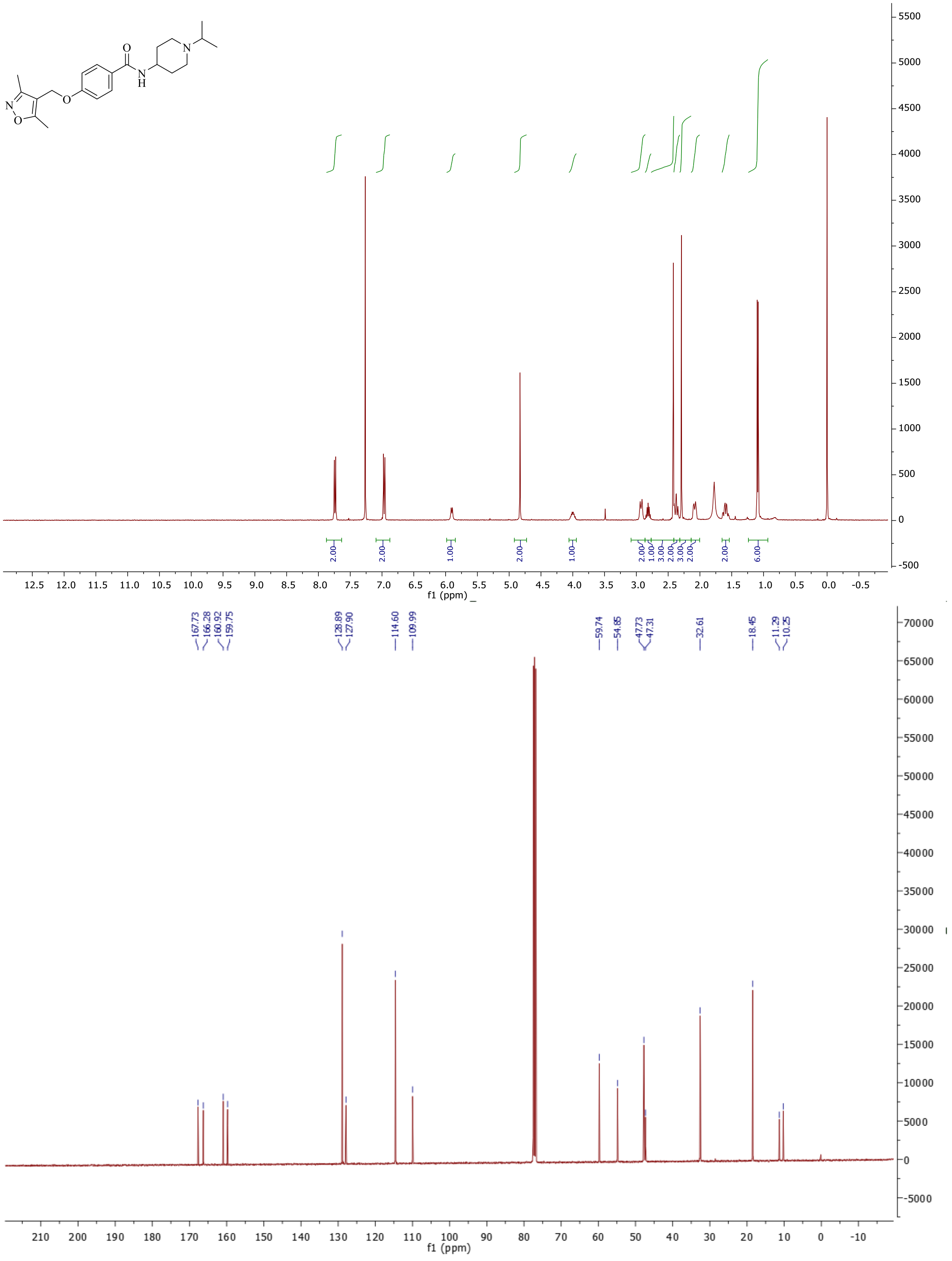

^{1}H and ^{13}C NMR for compound **35**.

Chromatogram

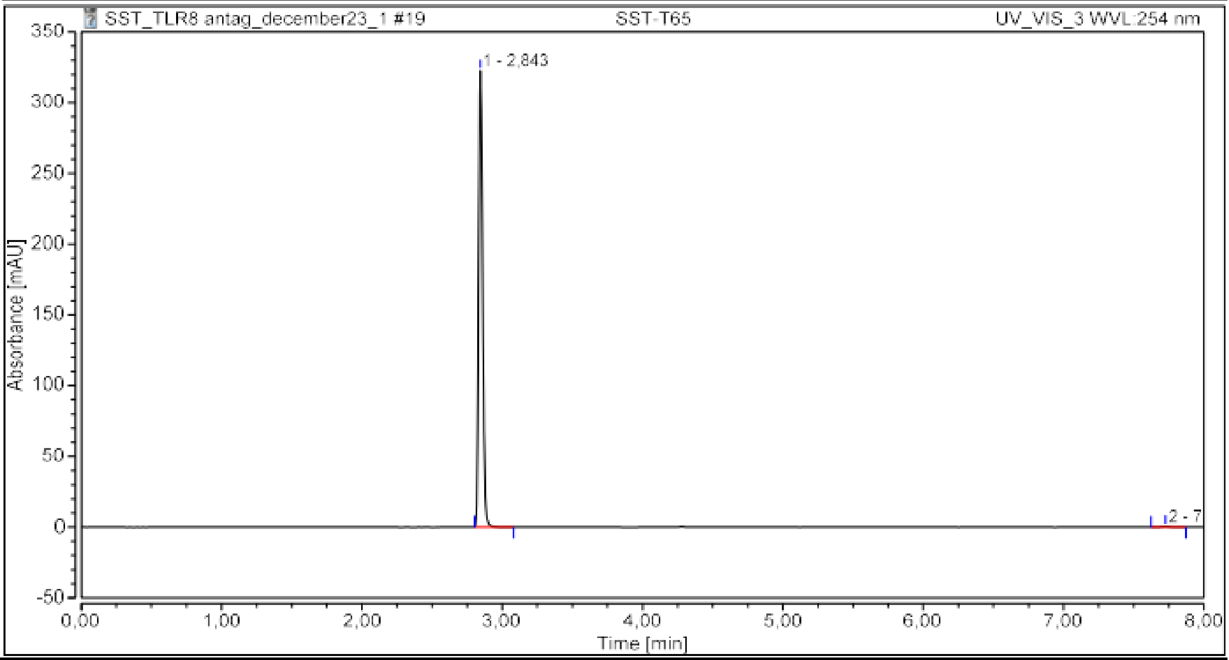

Integration Results

No.	Peak Name	Retention Time min	Area mAU*min	Height mAU	Relative Area %	Relative Height %	Amount n.a.
1		2,843	10,785	322,530	99,67	99,87	n.a.
2		7,727	0,035	0,415	0,33	0,13	n.a.
**Total:**			**10,821**	**322,945**	**100,00**	**100,00**	

HPLC trace for compound **35**.

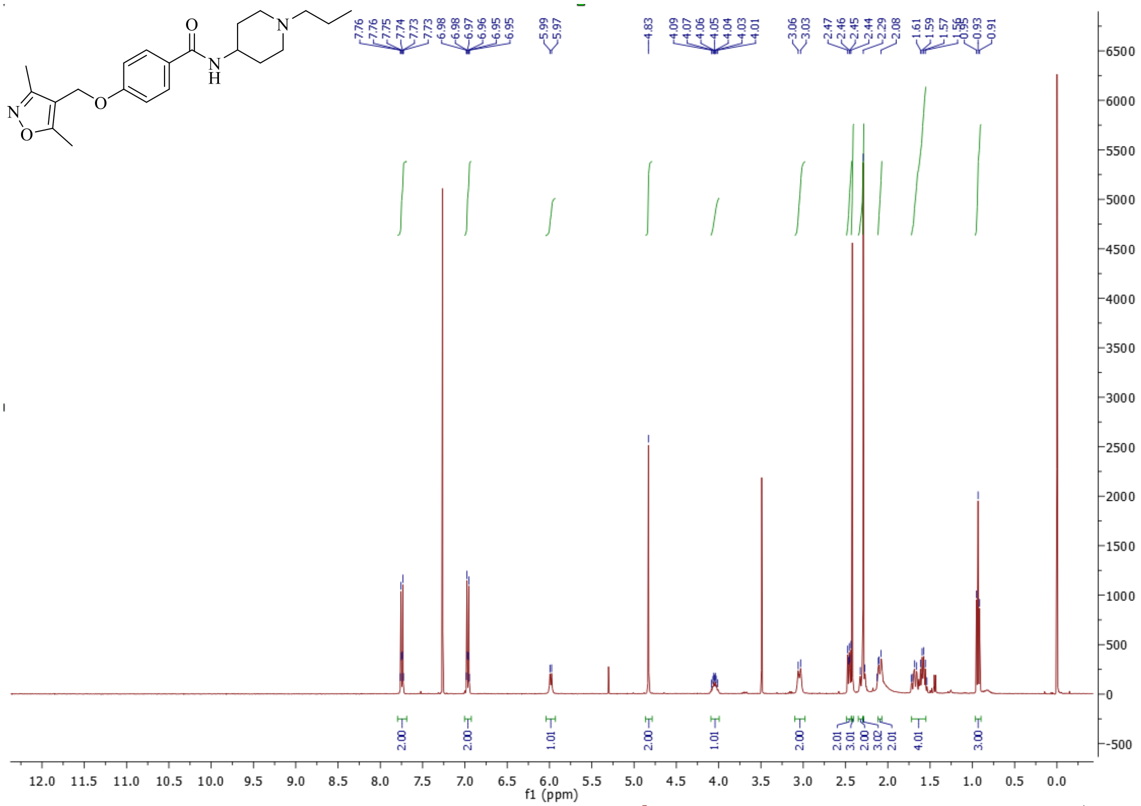

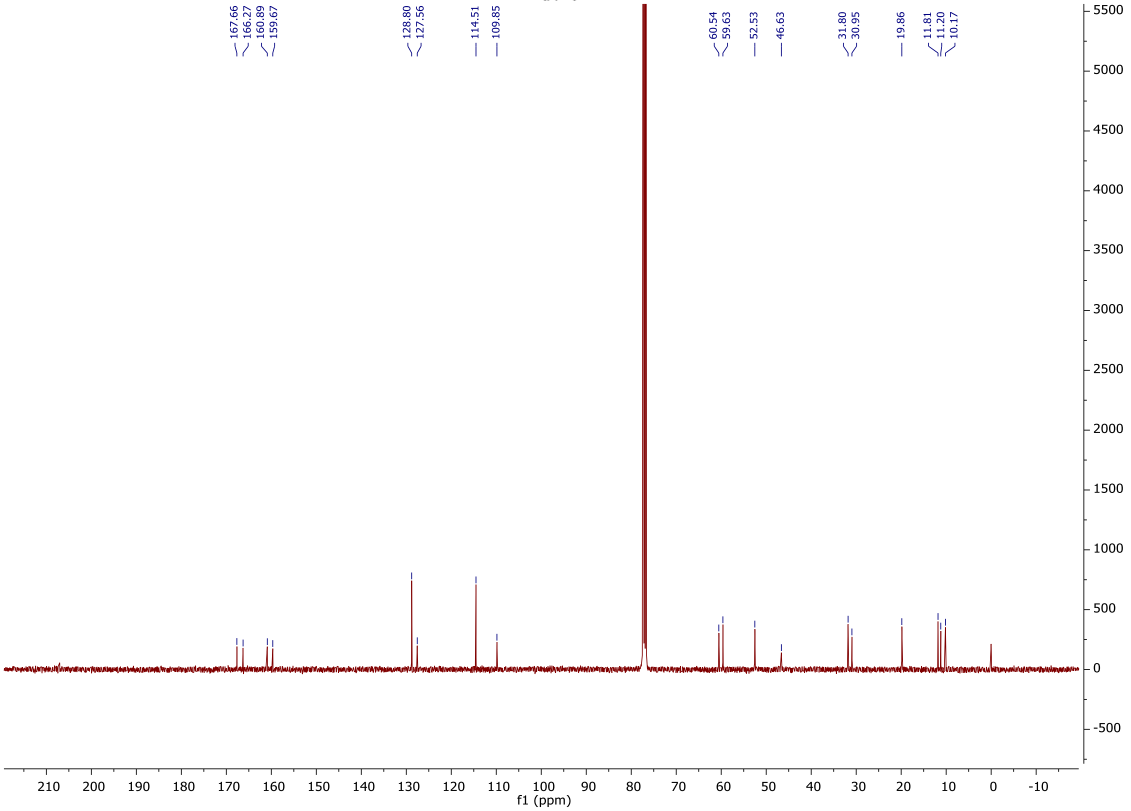

^{1}H and ^{13}C NMR for compound **38**.

Chromatogram

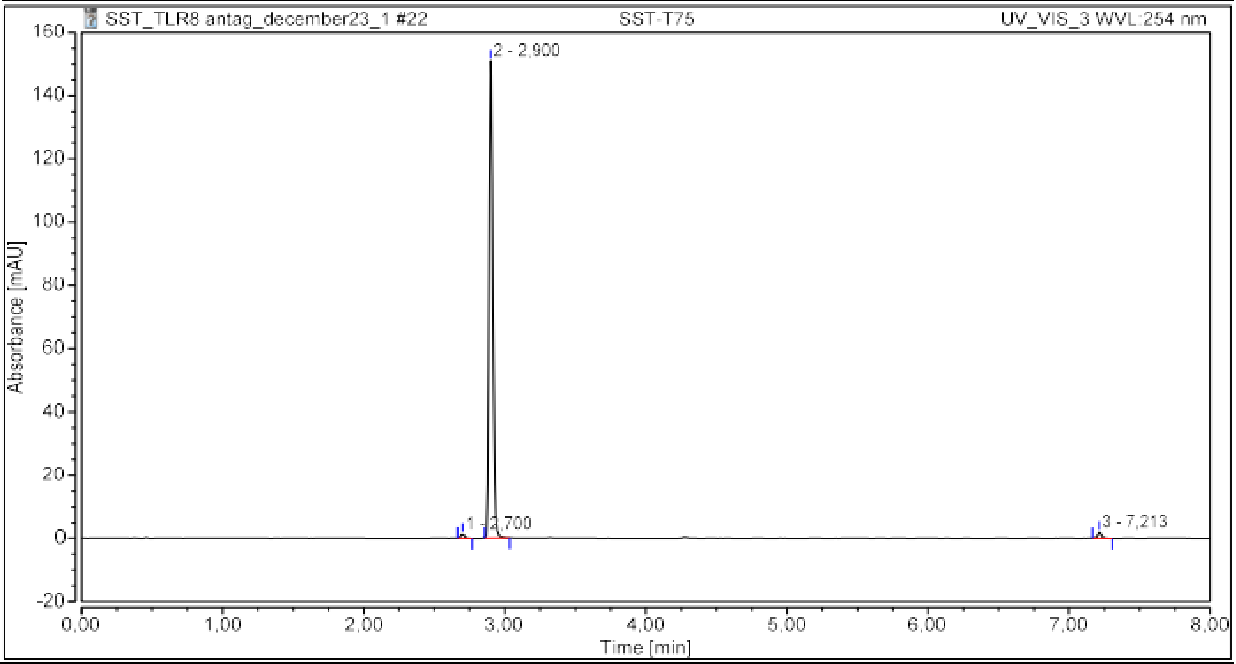

Integration Results

No.	Peak Name	Retention Time min	Area mAU*min	Height mAU	Relative Area %	Relative Height %	Amount n.a.
1		2,700	0,042	1,335	0,84	0,87	n.a.
2		2,900	4,870	150,941	97,87	97,87	n.a.
3		7,213	0,064	1,954	1,29	1,27	n.a.
**Total:**			**4,976**	**154,230**	**100,00**	**100,00**	

HPLC trace for compound **38**.

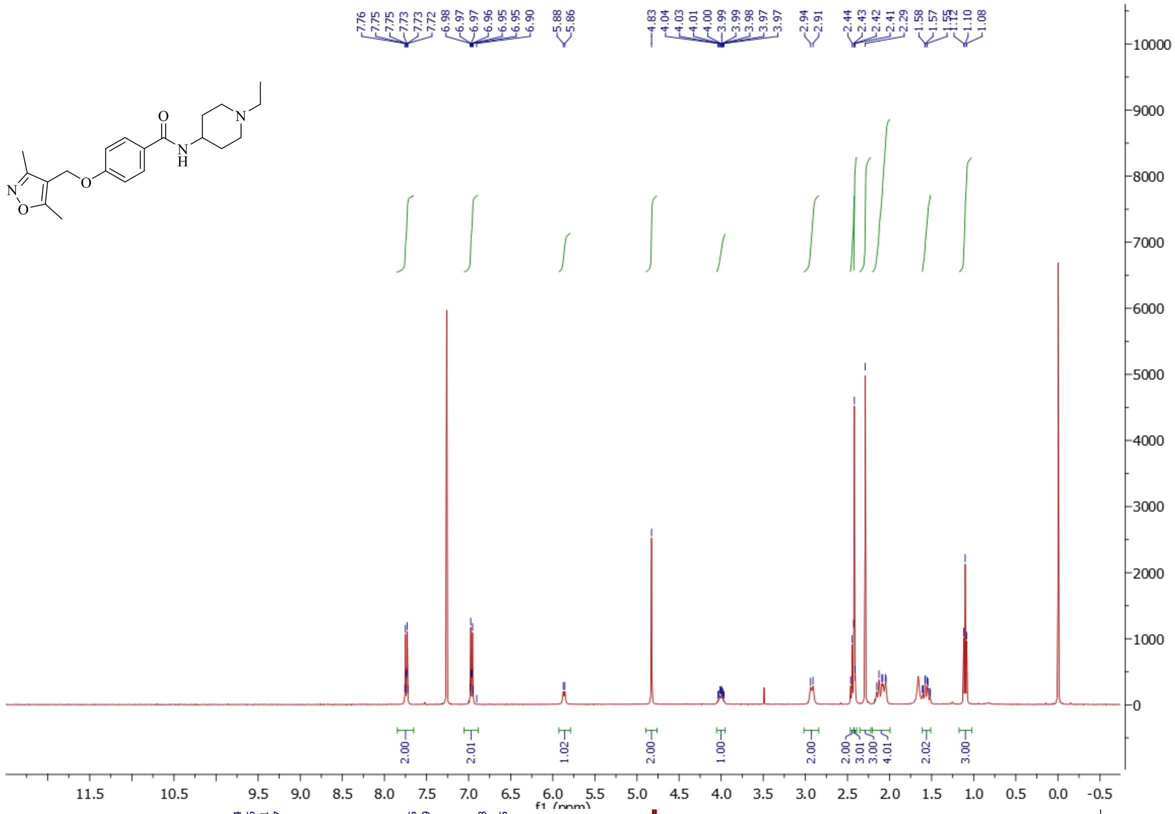

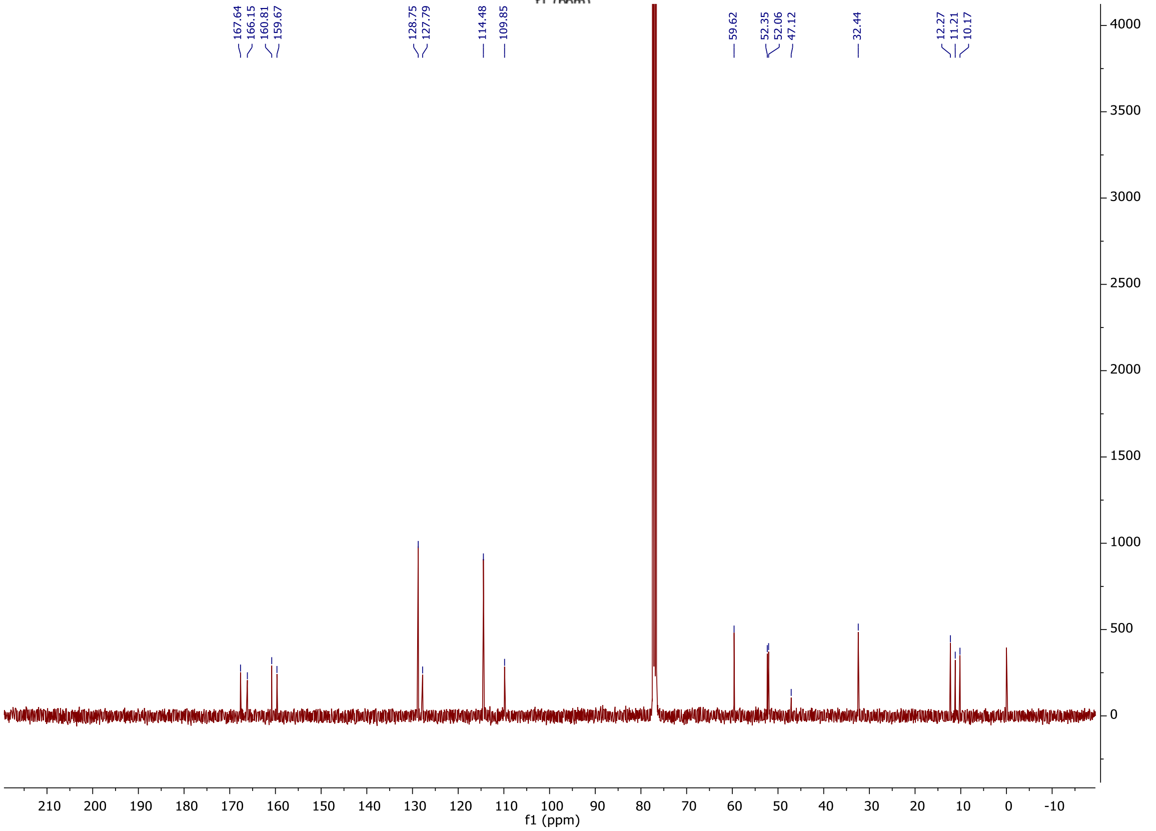

^{1}H and ^{13}C NMR for compound **39**.

Chromatogram

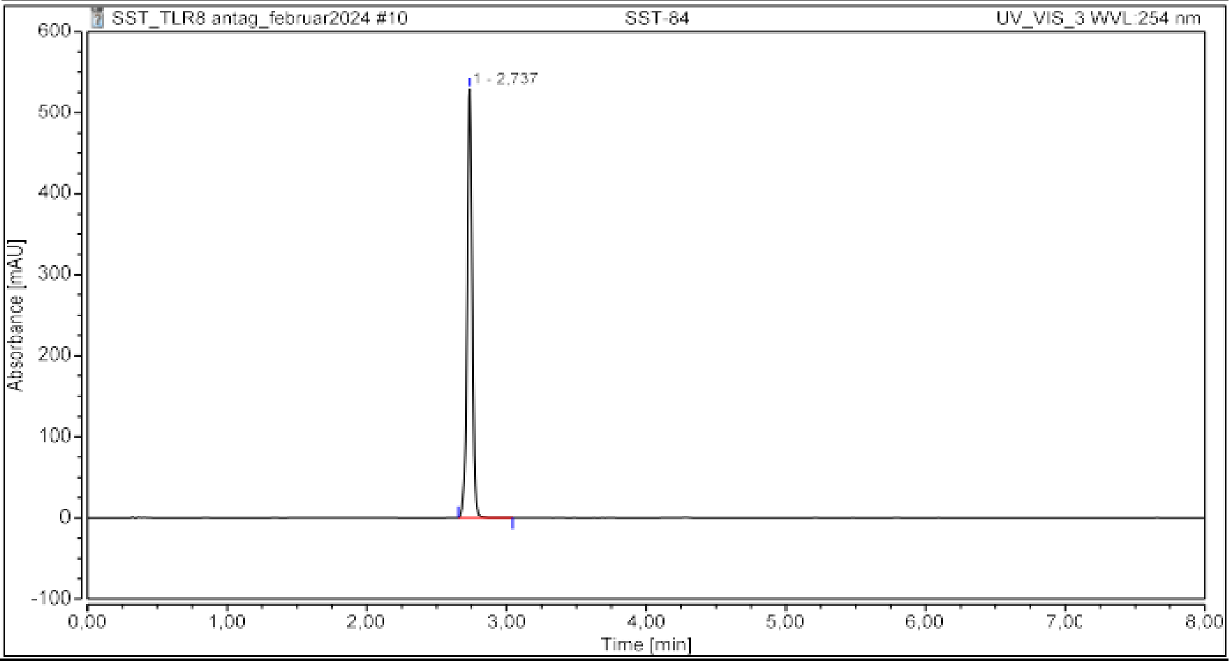

Integration Results

No.	Peak Name	Retention Time min	Area mAU*min	Height mAU	Relative Area %	Relative Height %	Amount n.a.
1		2,737	22,045	528,905	100,00	100,00	n.a.
**Total:**			**22,045**	**528,905**	**100,00**	**100,00**	

HPLC trace for compound **39**.

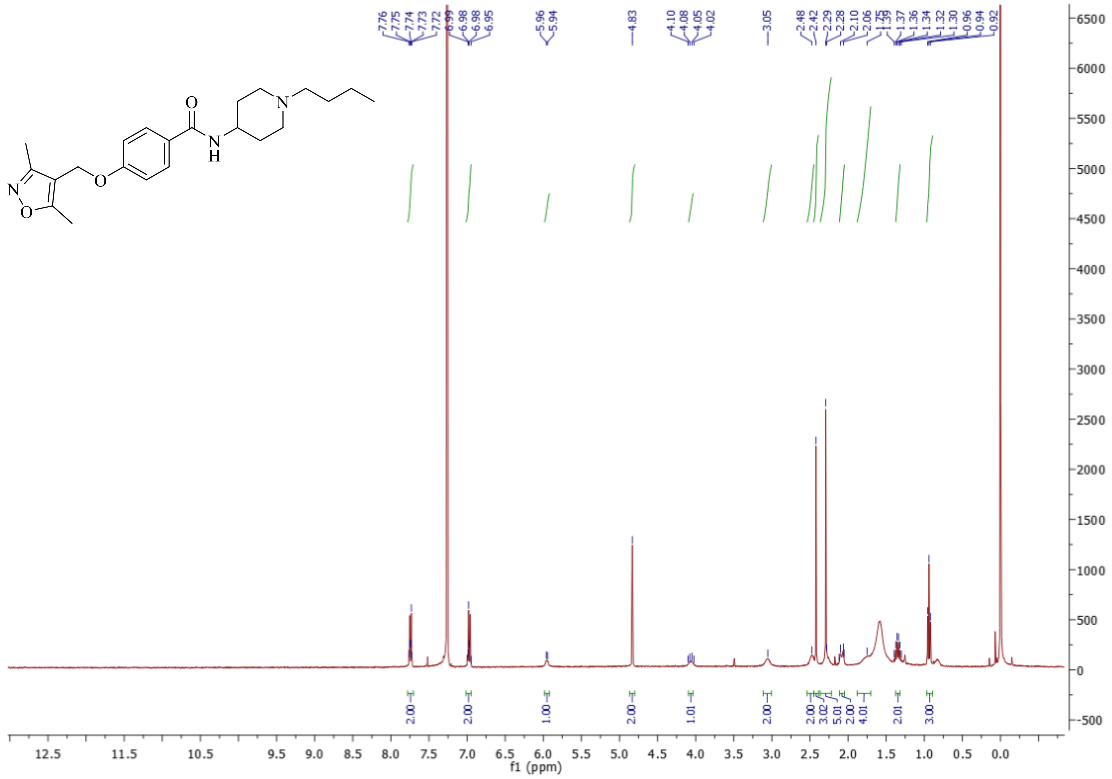

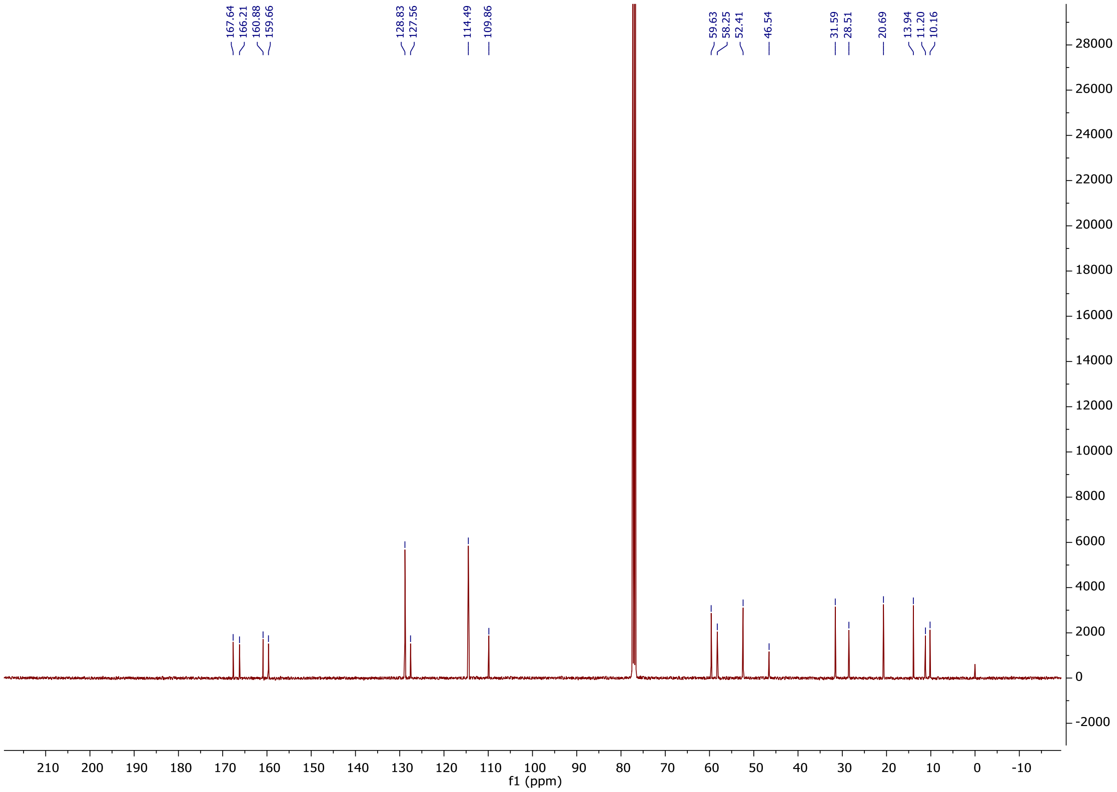

^{1}H and ^{13}C NMR for compound **40**.

Chromatogram

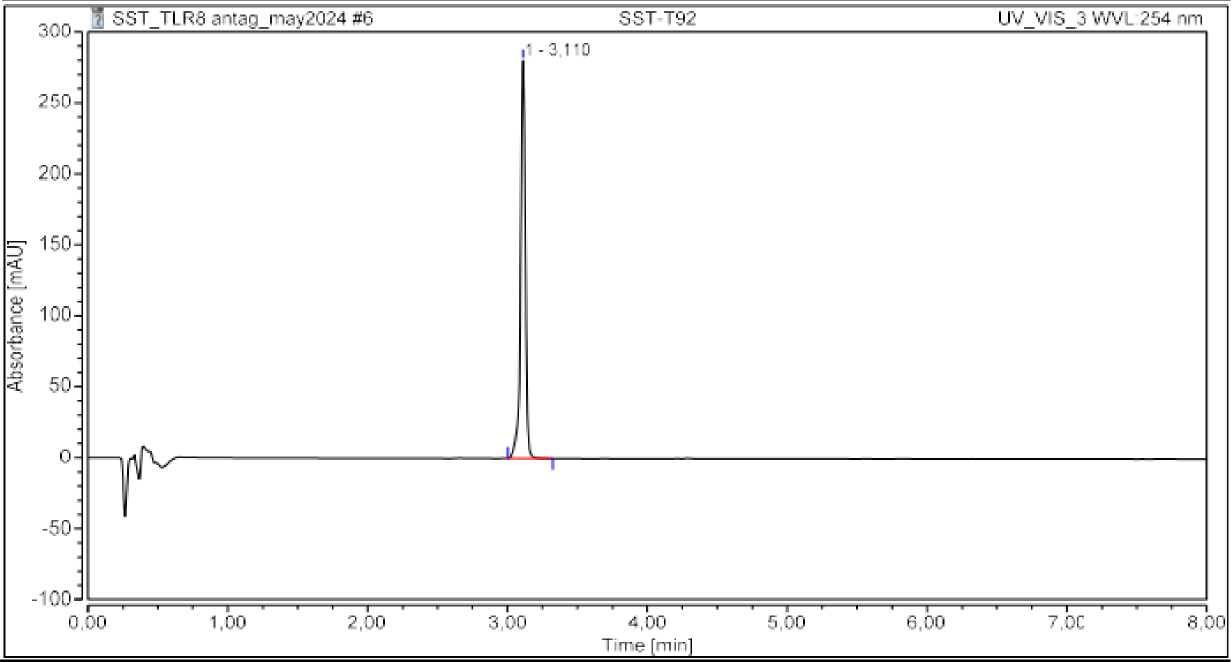

Integration Results

No.	Peak Name	Retention Time min	Area mAU*min	Height mAU	Relative Area %	Relative Height %	Amount n.a.
1		3,110	11,035	280,051	100,00	100,00	n.a.
**Total:**			**11,035**	**280,051**	**100,00**	**100,00**	

HPLC trace for compound **40**.

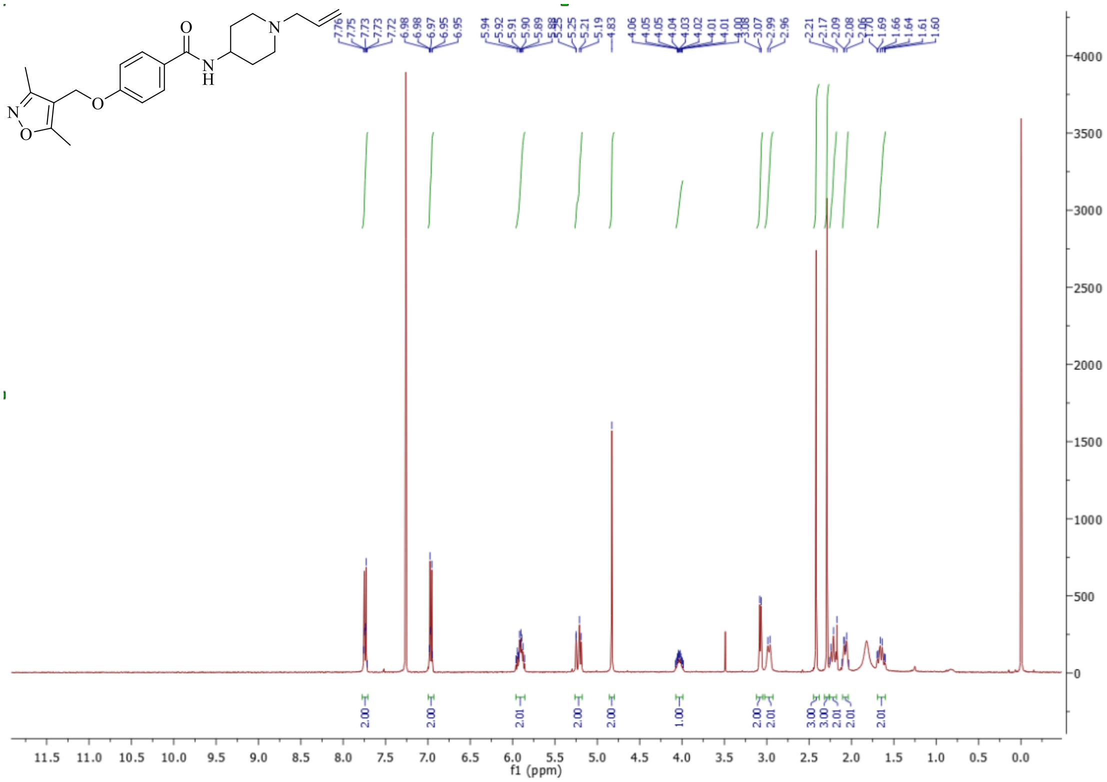

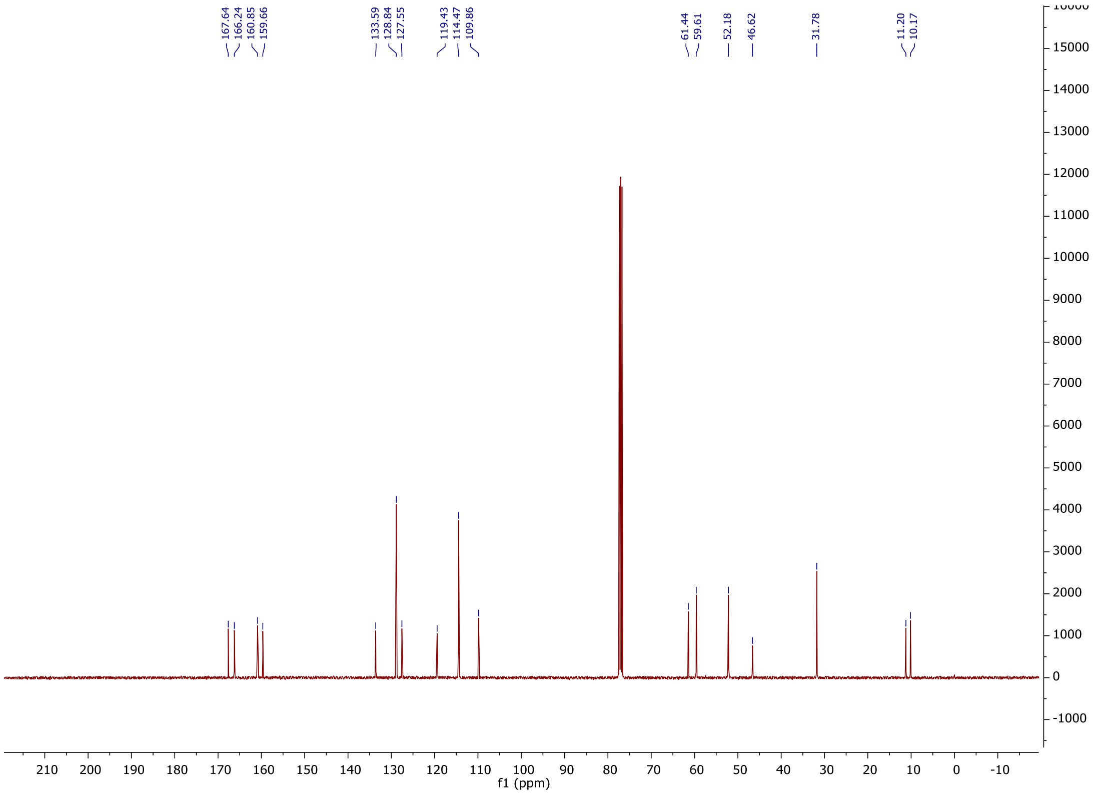

^{1}H and ^{13}C NMR for compound **41**.

Chromatogram

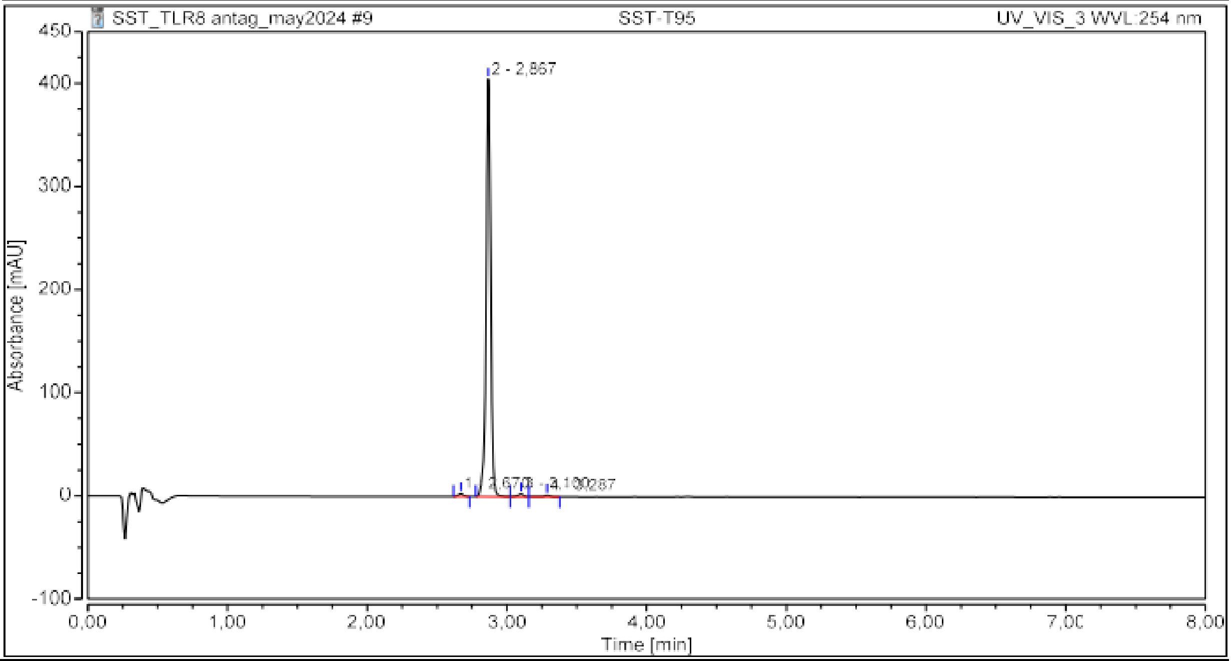

Integration Results

No.	Peak Name	Retention Time<br>min	Area<br>mAU*min	Height<br>mAU	Relative Area<br>%	Relative Height<br>%	Amount<br>n.a.
1		2,670	0,102	2,796	0,65	0,68	n.a.
2		2,867	15,440	404,575	98,08	98,31	n.a.
3		3,100	0,117	2,623	0,74	0,64	n.a.
4		3,287	0,083	1,529	0,53	0,37	n.a.
**Total:**			**15,742**	**411,523**	**100,00**	**100,00**	

HPLC trace for compound **41**.

Table S1. Chemical structures of compounds **1-12** containing the isoxazole scaffold.

Compound	Chemical Structure
1	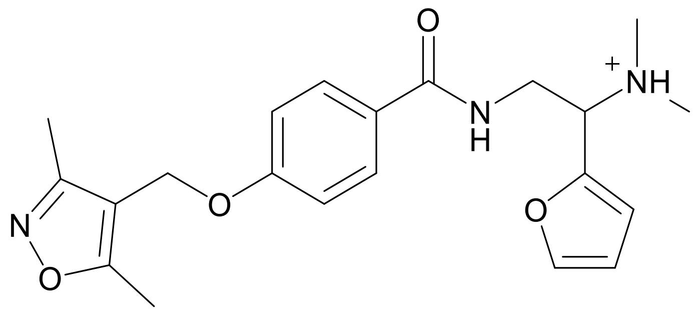
2	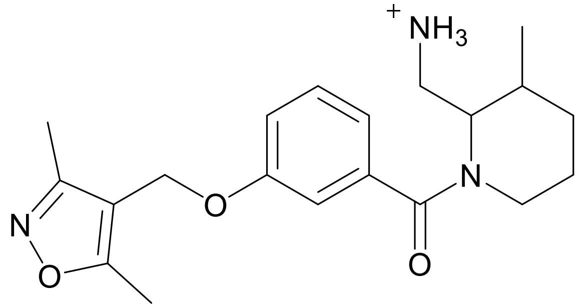
3	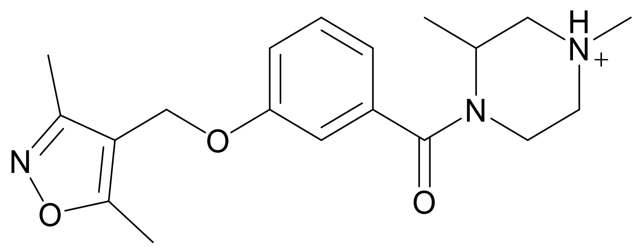
4	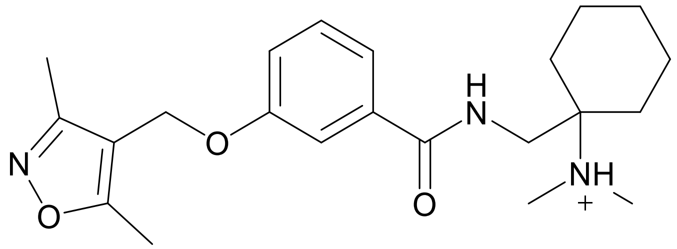

5	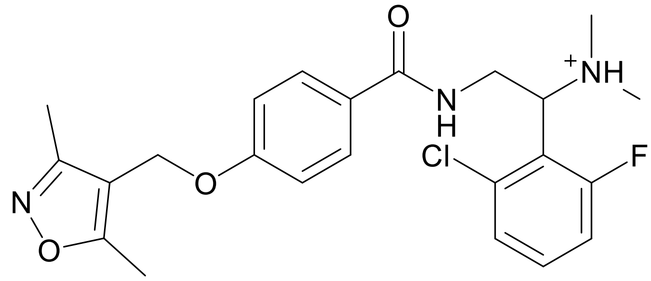
6	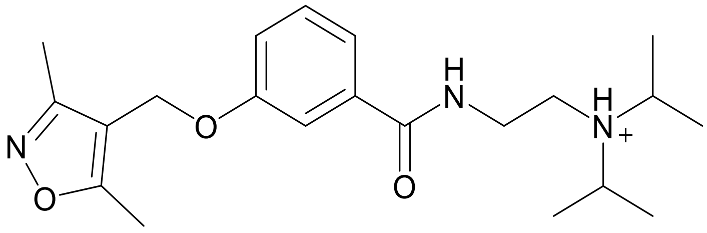
7	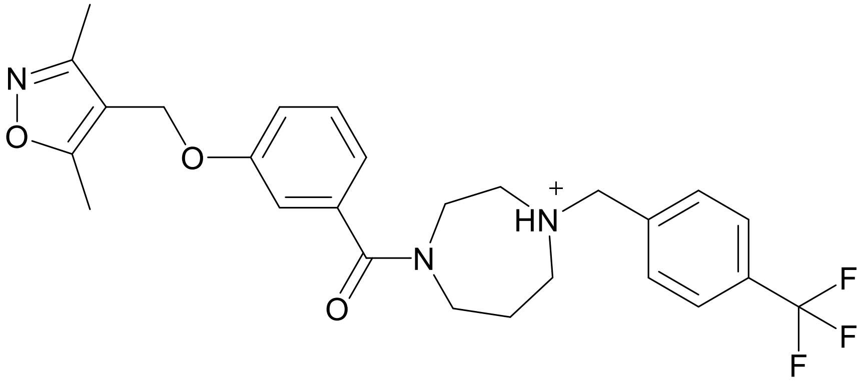
8	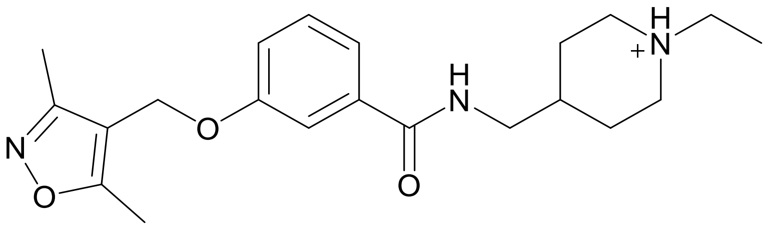
9	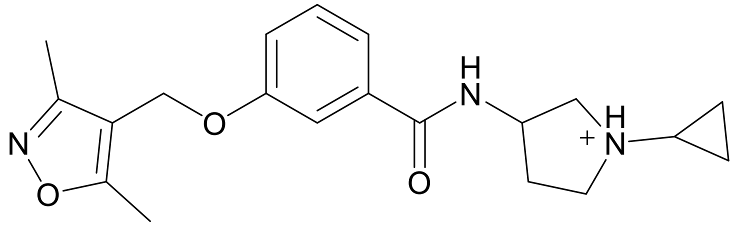

10	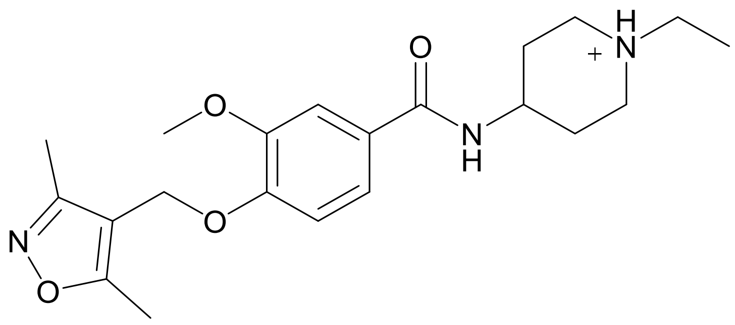
11	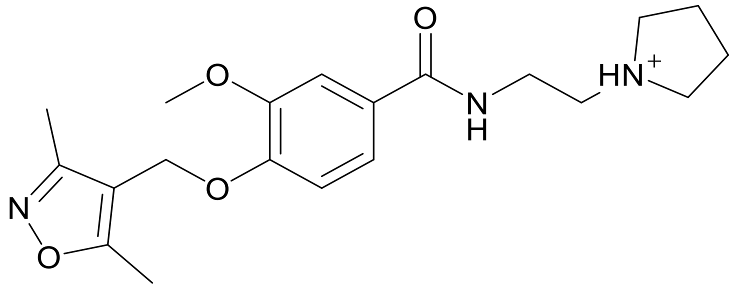
12	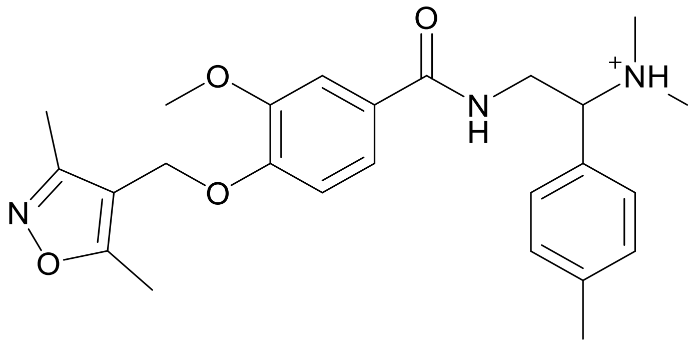

Table S2. Inhibition of NF-κB activity in hTLR8-HEK293 cells by compounds **1-12**.

Data are mean ± SEM of three independent experiments (Figure S3). One-sample *t*-test against 100% NF-κB activity. *P ≤ 0.05, ***P ≤ 0.001, ****P ≤ 0.0001.

**Compound**	**% inhibition at 10 μM ± SEM**
**1**	75 ± 4**
**2**	3 ± 2
**3**	-6 ± 2
**4**	16 ± 4
**5**	37 ± 7*
**6**	9 ± 4
**7**	5 ± 7
**8**	20 ± 4*
**9**	25 ± 9
**10**	98 ± 0.3****
**11**	75 ± 5**
**12**	83 ± 15*
Enpatoran	100 ± 0

Table S3. Inhibition of NF-κB activity in hTLR8-HEK293 cells by compounds **17-41**, **43**.

Data are mean ± SEM of three to four independent experiments (Figure S6). One-sample *t*-test against 100% NF-κB activity. *P ≤ 0.05, **P ≤ 0.01 ***P ≤ 0.001, ****P ≤ 0.0001.

**Compound**	**% inhibition at 10 µM ± SEM**
**17**	52 ± 4***
**18**	61 ± 3***
**19**	89 ± 2****
**20**	63 ± 0.7****
**21**	75 ± 2****
**22**	54 ± 3****
**23**	57 ± 3***
**24**	96 ± 2**
**25**	68 ± 6****
**26**	77 ± 4**
**27**	84 ± 4***
**28**	97 ± 1****
**29**	72 ± 5***
**30**	24 ± 9
**31**	98 ± 0.3****
**32**	77 ± 5***
**33**	94 ± 2 ****
**34**	97 ± 0.9****
**35**	99 ± 0.2****
**36**	26 ± 2***
**37**	80 ± 2****
**38**	100 ± 0.1****
**39**	100 ± 0.2****
**40**	99 ± 0.4****
**41**	99 ± 0.7****
**43**	32 ± 2**
Enpatoran	100 ± 0****

Table S4. Chemical structures of synthesized compounds **17-41**, **43**.

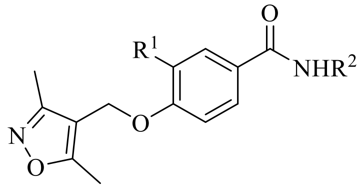

Compound	R^1	Amine (R_2NH_2)
**17**	H	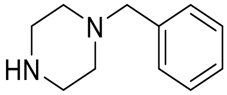
**18**	H	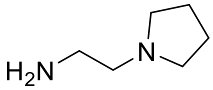
**19**	H	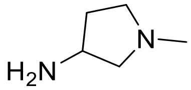
**20**	H	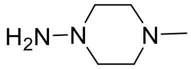
**21**	H	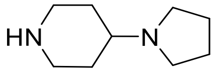
**22**	OMe	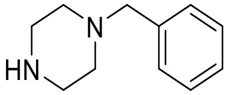
**23**	OMe	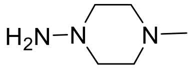
**24**	OMe	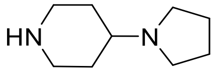
**25**	OMe	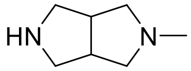
**26**	OMe	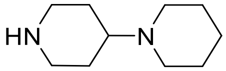
**27**	OMe	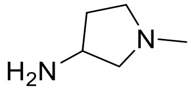
**28**	OMe	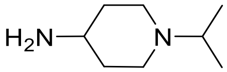
**29**	OMe	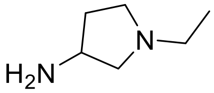
**30**	OMe	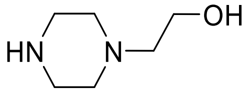
**31**	OMe	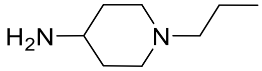
**32**	OMe	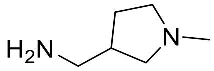

**33**	OMe	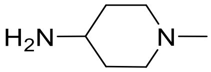
**34**	H	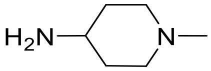
**35**	H	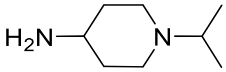
**36**	H	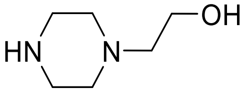
**37**	H	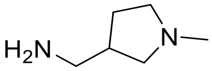
**38**	H	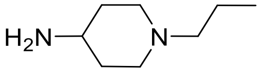
**39**	H	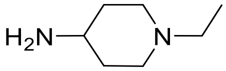
**40**	H	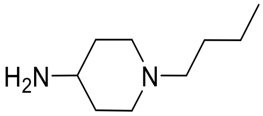
**41**	H	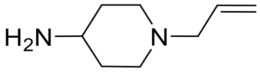
**43**	H	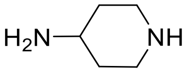

Table S5. Interaction frequencies of **10** with TLR8 homodimer during MD simulation.

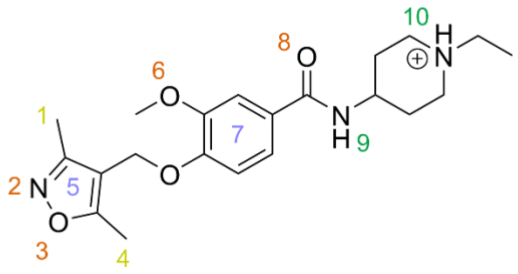

**Ligand Moiety**	**Interaction type**	**Frequency**
Methyl 1	Hydrophobic	100.0%
Isoxazole nitrogen 2	Hydrogen bond acceptor	60.1%
Isoxazole oxygen 3	Hydrogen bond acceptor	80.2%
Methyl 4	Hydrophobic	100.0%
Isoxazole 5	Aromatic	43.5%
Methoxy 6	Hydrogen bond acceptor	7.3%
Phenyl 7	Hydrophobic	100.0%
Carbamide oxygen 8	Hydrogen bond acceptor	20.5%
Carbamide amide 9	Hydrogen bond donor	8.3%
Amine 10	Hydrogen bond donor	84.4%
Amine 10	Positive ionizable	91.6%

Table S6. Pharmacological parameters (EC_{50}, E_{max}) of compound **10.**
Values were calculated from curves shown in Figure 6C.

	**EC_{50} [µM] (95% CI)**	**EC_{max} [%] (95% CI)**
TL8-506	0.19 (0.16-0.22)	97.6 (92.9-104.7)
+ **10** (0.1 µM)	0.27 (0.24-030)	95.42 (91.6-99.8)
+ **10** (0.5 µM)	0.33 (0.29-0.39)	94.8 (89.9-100.4)
+ **10** (1 µM)	0.49 (0.40-0.60)	93.9 (86.9-102.1)
+ **10** (5 µM)	0.93 (0.79-1.16)	94.26 (84.5-111.8)
+ **10** (10 µM)	1.45 (1.39-1.58)	103.7 (95.5-114.2)

References

(1) Schaller, D.; Šribar, D.; Noonan, T.; Deng, L.; Nguyen, T. N.; Pach, S.; Machalz, D.; Bermudez, M.; Wolber, G. Next Generation 3D Pharmacophore Modeling. *WIREs Comput Mol Sci* **2020**, *10* (4), e1468. https://doi.org/10.1002/wcms.1468.

(2) Kutlushina, A.; Khakimova, A.; Madzhidov, T.; Polishchuk, P. Ligand-Based Pharmacophore Modeling Using Novel 3D Pharmacophore Signatures. *Molecules* **2018**, *23* (12), 3094. https://doi.org/10.3390/molecules23123094.

(3) Noonan, T.; Denzinger, K.; Talagayev, V.; Chen, Y.; Puls, K.; Wolf, C. A.; Liu, S.; Nguyen, T. N.; Wolber, G. Mind the Gap—Deciphering GPCR Pharmacology Using 3D Pharmacophores and Artificial Intelligence. *Pharmaceuticals* **2022**, *15* (11), 1304. https://doi.org/10.3390/ph15111304.

(4) Mendez, D.; Gaulton, A.; Bento, A. P.; Chambers, J.; De Veij, M.; Félix, E.; Magariños, M. P.; Mosquera, J. F.; Mutowo, P.; Nowotka, M.; Gordillo-Marañón, M.; Hunter, F.; Junco, L.; Mugumbate, G.; Rodriguez-Lopez, M.; Atkinson, F.; Bosc, N.; Radoux, C. J.; Segura-Cabrera, A.; Hersey, A.; Leach, A. R. ChEMBL: Towards Direct Deposition of Bioassay Data. *Nucleic Acids Research* **2019**, *47* (D1), D930–D940. https://doi.org/10.1093/nar/gky1075.

(5) Gaulton, A.; Hersey, A.; Nowotka, M.; Bento, A. P.; Chambers, J.; Mendez, D.; Mutowo, P.; Atkinson, F.; Bellis, L. J.; Cibrián-Uhalte, E.; Davies, M.; Dedman, N.; Karlsson, A.; Magariños, M. P.; Overington, J. P.; Papadatos, G.; Smit, I.; Leach, A. R. The ChEMBL Database in 2017. *Nucleic Acids Res* **2017**, *45* (D1), D945–D954. https://doi.org/10.1093/nar/gkw1074.

(6) Gaulton, A.; Bellis, L. J.; Bento, A. P.; Chambers, J.; Davies, M.; Hersey, A.; Light, Y.; McGlinchey, S.; Michalovich, D.; Al-Lazikani, B.; Overington, J. P. ChEMBL: A Large-Scale Bioactivity Database for Drug Discovery. *Nucleic Acids Research* **2012**, *40* (D1), D1100–D1107. https://doi.org/10.1093/nar/gkr777.

(7) Mysinger, M. M.; Carchia, M.; Irwin, John. J.; Shoichet, B. K. Directory of Useful Decoys, Enhanced (DUD-E): Better Ligands and Decoys for Better Benchmarking. *J. Med. Chem.* **2012**, *55* (14), 6582–6594. https://doi.org/10.1021/jm300687e.
